# Serum Oxylipin Profiles Identify Potential Biomarkers in Patients with Acute Aortic Dissection

**DOI:** 10.3390/metabo12070587

**Published:** 2022-06-23

**Authors:** Yi Jiang, Xinlong Tang, Yali Wang, Wei Chen, Yunxing Xue, Hailong Cao, Bomin Zhang, Jun Pan, Qing Zhou, Dongjin Wang, Fudong Fan

**Affiliations:** 1 Nanjing Drum Tower Hospital Clinical College of Nanjing Medical University, Nanjing 210008, China; 2 Department of Thoracic and Cardiovascular Surgery, The Affiliated Drum Tower Hospital of Nanjing University Medical School, Nanjing 210008, China; 3 Institute of Cardiothoracic Vascular Disease, Nanjing University, Nanjing 210008, China

**Keywords:** aortic dissection, oxylipin, LC-MS/MS, biomarker

## Abstract

Aortic dissection (AD) is a life-threatening cardiovascular disease with a dismal prognosis. Inflammation plays an important role in AD. Oxylipins are bioactive lipids involved in the modulation of inflammation and may be involved in the pathogenesis and progression of AD. This study aims to identify possible metabolites related to AD. A total of 10 type A Aortic dissection (TAAD) patients, 10 type B Aortic dissection (TBAD) patients and 10 healthy controls were included in this study. Over 100 oxylipin species were identified and quantified by liquid chromatography with tandem mass spectrometry (LC-MS/MS) analysis. Our investigation demonstrated substantial alterations in 91 oxylipins between AD and healthy individuals. Patients with TAAD had 89 entries accessible compared to healthy controls. According to orthogonal partial least squares discriminant analysis (OPLS-DA), fitness (R^2^X = 0.362 and R^2^Y = 0.807, *p* = 0.03) and predictability (Q^2^ = 0.517, *p* = 0.005) are the validation parameters between the two groups. Using multivariate logistic regression, 13-HOTrE and 16(17)-EpDPE were the risk factors in the aortic patients group compared to healthy people (OR = 2.467, 95%CI:1.256–7.245, *p* = 0.035; OR = 0.015, 95%CI:0.0002–0.3240, *p* = 0.016, respectively). In KEGG enrichment of differential metabolites, the arachidonic acid metabolism pathway has the most metabolites involved. We established a diagnostic model in distinguishing between AD and healthy people. The AUC was 0.905. Oxylipins were significantly altered in AD patients, suggesting oxylipin profile is expected to exploit a novel, non-invasive, objective diagnosis for AD.

## 1. Introduction

Aortic dissection (AD) is a lethal cardiovascular disease. The aortic wall comprises three layers: the intima, the media and the adventitia. Blood flowing into the media layer leads to the formation of a true and a false lumen [1,2]. According to the Stanford classification, type A aortic dissection involves the ascending aorta, whereas type B affects the descending wall of the aorta. More than half of the patients died within 48 h if untreated [2,3]. Many patients missed timely treatment because of delayed diagnosis [4]. Due to poor understanding of the mechanism and pathophysiology process of AD, there are still many problems in the exploration of new diagnostic biomarkers.

Extracellular matrix degradation and inflammation are two main mechanisms of medial degeneration in AD, while smooth muscle damage, cardiac stress and thrombosis/fibrinolysis are also important pathophysiology processes during AD formation and development [5,6]. Biomarkers from those categories, such as D-dimer, MMPs, sST2 and cTNT, are all not specific enough for AD [7,8,9]. Inflammation plays a key role in the progression of AD, while common inflammatory biomarkers C-reactive protein (CRP) and Interleukin-6 (IL-6) have poor specificity and efficiency [10,11,12]. Further exploration is needed in inflammation indicators in AD.

Oxylipins are bioactive lipids in the modulation of inflammatory responses [13,14]. Oxylipins were already investigated in inflammatory conditions such as obesity, asthma, and arthritis [15,16,17,18]. They are lipids derived from polyunsaturated fatty acids (PUFAs) including ω-3 PUFAs and ω-6 PUFAs. Arachidonic acid (ARA), linolenic acid (LA), γ-linolenic acid (GLA) and dihomo-γ-linolenic acid (DGLA) are known as ω-6 PUFAs, which are precursors of major pro-inflammatory oxylipins. While ω-3 PUFAs (eicosapentaenoic acid (EPA), docosahexaenoic acid (DHA)) are considered precursors of anti-inflammatory oxylipins and specialized pro-resolving mediators [19].

Oxylipins influence numerous physiological and pathological processes [20,21,22]. Furthermore, the function of oxylipins in acute coronary syndrome (ACS) or other cardiovascular disorders has been reported [23,24,25,26]. Here, we demonstrated altered oxylipins in individuals with AD and healthy individuals. To our knowledge, this is the first study on oxylipin profiles in AD. The study might help researchers find potential biomarkers of AD for early diagnosis.

## 2. Results

### 2.1. Clinical Characteristics of the Studied Population

**Figure 1:**
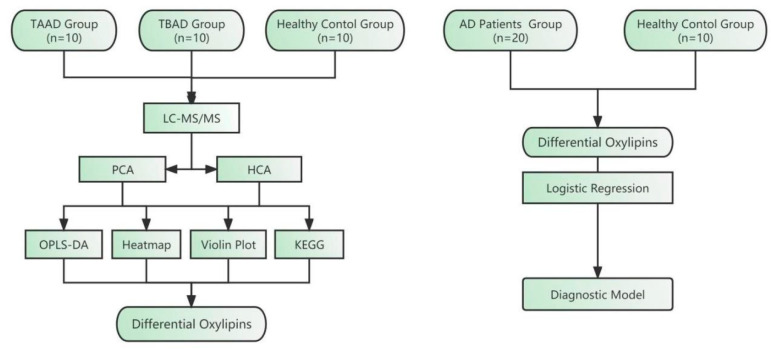
Flow diagram of this study design. Serum samples of aortic dissection (AD) patients and healthy controls were collected to detect the content of oxylipins. Differential oxylipins were screened by a series of methods and a diagnostic model was established. TAAD, type A aortic dissection. TBAD, type B aortic dissection. LC-MS/MS, Liquid chromatography with tandem mass spectrometry. PCA, Principal component analysis. HCA, Hierarchical cluster analysis. OPLS-DA, Orthogonal partial least squares discriminant analysis. KEGG, Kyoto Encyclopedia of Genes and Genomes.

Our study included 20 patients (10 with type A Aortic dissection (TAAD) and 10 with type B Aortic dissection (TBAD)) and 10 controls. The mean age of patients is 47.40 ± 12.77 in Group A, 52.20 ± 13.05 in Group B and 49.10 ± 4.91 in Group C (*p* = 0.790). The average BMI was 28.09 ± 6.26 in Group A, 27.78 ± 9.04 in Group B and 24.81 ± 3.73 in Group C (*p* = 0.491). There were also no significant differences in gender and hypertension history among the three groups (*p* = 0.153 and 0.303). There were also significant differences in triglycerides (*p* = 0.007), cholesterol (*p* = 0.001), L-cholesterol (*p* = 0.006), albumin (*p* < 0.001), neutrophil percentage (*p* < 0.001) and total protein (*p* < 0.001) (Table 1). A flow diagram of this study is shown in Figure 1.

### 2.2. Data Preprocessing and PCA Analysis of Oxylipins

Total ion chromatogram (TIC) and extraction ion chromatogram (XIC) of samples are presented in Supplementary Figure S1A. Sample quantitative analysis integral correction results are presented in Supplementary Figure S1B. In total, we examined 120 oxylipin standards, including 8 of LA-derived oxylipin, 14 of ARA-derived oxylipin, 10 of EPA-derived oxylipin, and 11 of DHA-derived oxylipin.

**Figure 2:**
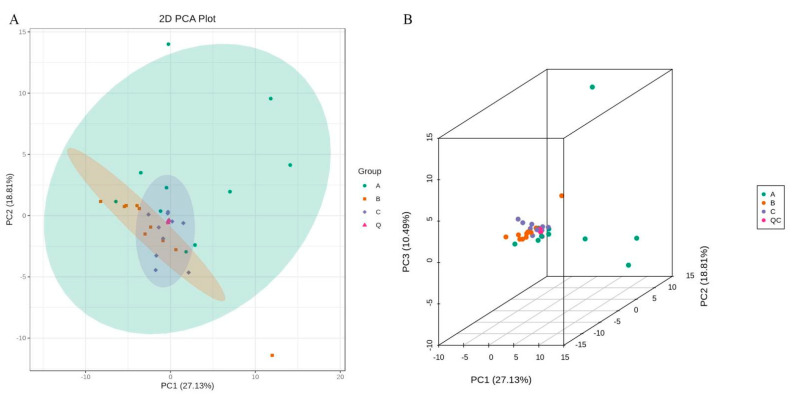
Oxylipin profile is different in AD patients and healthy controls. (**A**) PLS-DA score plot of AD patients with TAAD (*n* = 10) and TBAD (*n* = 10) versus healthy controls (*n* = 10). The explained variances for each component are shown in brackets. (**B**) PLS-DA 3D score plot.

The samples were subjected to principal component analysis (PCA) in order to gain a preliminary understanding of the overall metabolic differences and the degree of variation among the three groups. The PCA score plot demonstrated the separation trend of oxylipins within each group, indicating whether there were differences in oxylipins between sample groups (Figure 2). Supplementary Figure S1C shows the explicable variation of PCA.

**Figure 3:**
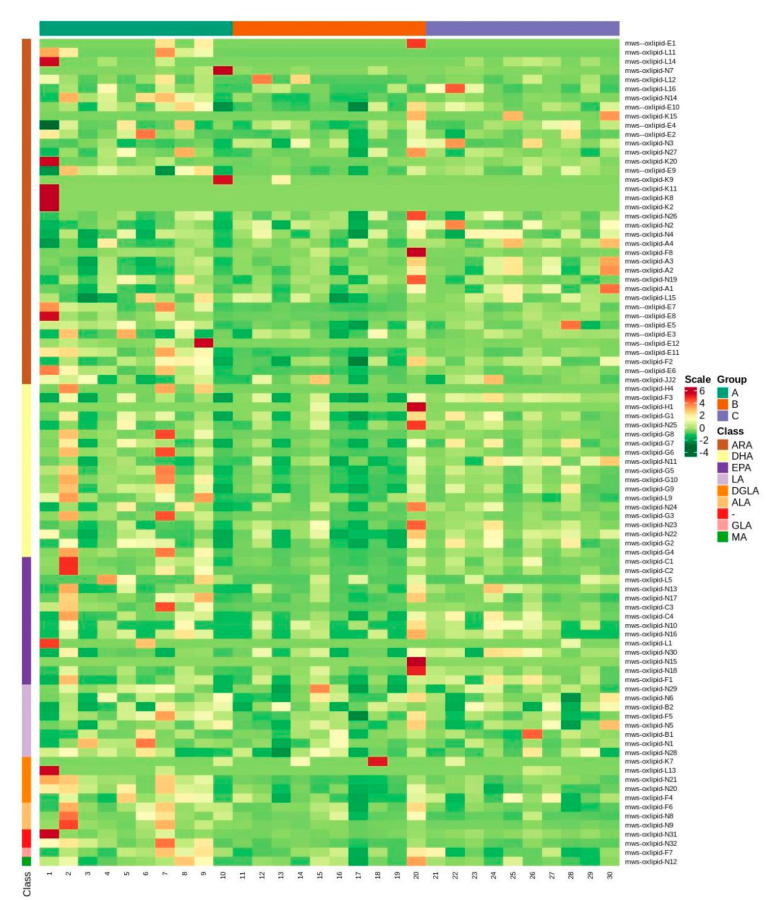
Heatmap of cluster analysis which shows different patterns among the three groups.

For metabolite accumulation patterns among different samples, the metabolite content data were normalized using unit variance scaling and hierarchical cluster analysis (HCA). The data was standardized, and a cluster heat-map analysis was carried out (Figure 3 and Supplementary Figure S2).

### 2.3. Differential Metabolite Screening

**Figure 4:**
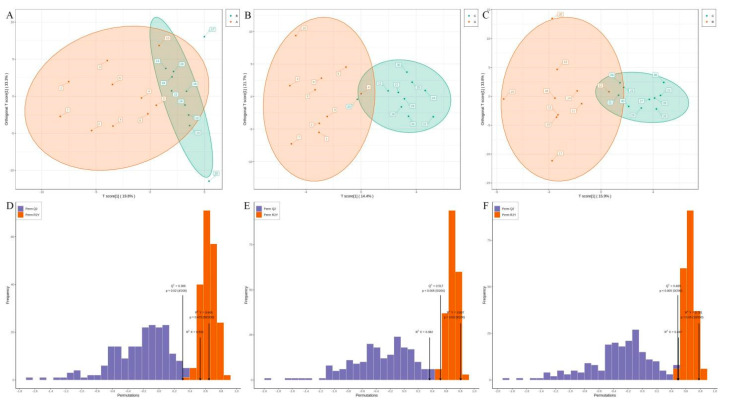
Summary and validation of the OPLS-DA models. (**A**–**C**) OPLS-DA score plots for Group A versus Group B (**A**), Group A versus Group C (**B**), and Group B versus Group C (**C**). The abscissa represents the predicted principal component and the ordinate represents the orthogonal principal component. (**D**–**F**) OPLS-DA permutation test plot for Group A versus Group B (**D**), Group A versus Group C (**E**), and Group B versus Group C (**F**). The abscissa represents model accuracy, and the ordinate represents the frequency of model classification effect.

The orthogonal partial least squares discriminant analysis (OPLS-DA) revealed that the three groups shared comparable common metabolites. OPLS-DA can improve group distinction and make the search for differential metabolites easier. Patients with TAAD had 89 entries accessible compared to healthy controls. The metabolite content of (±)16,17-epoxy-4Z,7Z,10Z,13Z,19Z-docosapentaenoic acid (16(17)-EpDPE) and (±)-14(15)-Epoxy-5Z,8Z,11Z,17Z-Eicosatetraenoic Acid ((±)14(15)-EpETE) differed among the three groups. The OPLS-DA score plot was 19.8%, 14.4% and 15.9% in three pairwise comparisons, respectively (Figure 4A–C). Fitness (R^2^X = 0.362 and R^2^Y = 0.807, *p* = 0.030) and predictability (Q^2^ = 0.517, *p* = 0.005) validation parameters in Group C contrast with those in Group A. However, the validation metrics of fitness (R^2^X = 0.531 and R^2^Y = 0.645, *p* = 0.475) and predictability (Q^2^ = 0.306, *p* = 0.020) in Group B are less differentiated in models than in Group A (Figure 4D–F). In addition, Q^2^ = 0.517 with *p* < 0.005 was measured for a single OPLS-DA model, particularly group A against group C. An OPLS-DA S-Plot is used to demonstrate the difference between principal components and metabolites while looking for the lipids that most significantly contributed to class separation. The red dots show that the variable importance in projection (VIP) methodology values for these metabolites are greater than or equal to 1. In all, 38 lipids had a VIP score greater than one; the fold changes for these lipids are presented in Supplementary Figure S3.

**Figure 5:**
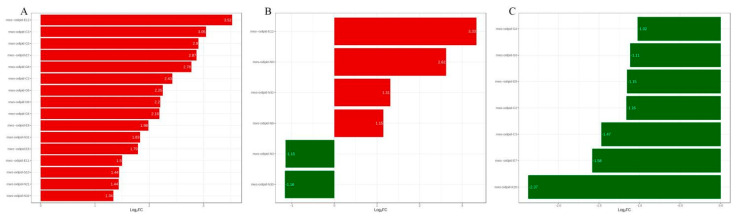
Bar charts of differential metabolites for Group A and Group B (**A**), Group A versus Group C (**B**), and Group B versus Group C (**C**).

After logarithmic processing, the fold changes of metabolites were displayed in a bar chart. Patients with TAAD exhibit 15 distinct metabolite species compared to Group B. The most up-regulated and down-regulated metabolites were 8S-hydroxy-4Z,6E,10Z-hexadecatrienoic acid (tetranor-12(S)-HETE) and (±)-12-hydroxy-5Z,8Z,10E,14Z,17Z-eicosapentaenoic acid ((±)12-HEPE), with differential multiples of 3.52 and 3.05 in Group B versus Group A, respectively. Tetranor-12(S)-HETE demonstrated a greater multiple of difference between Groups B and C than Group A. However, the differential multiples in 12-Hydroxy-5,8,10-heptadecatrienoic acid (12-HHT) between Group B and Group C is −2.37 (Figure 5).

**Figure 6:**
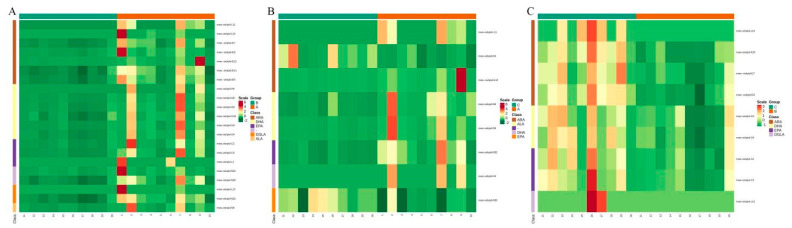
Cluster heatmaps of differential metabolites for Group A versus Group B (**A**), Group A versus Group C (**B**), and Group B versus Group C (**C**).

The original contents of the differential metabolites were standardized using unit variance scaling, and heat-map analysis was used to establish that patients with TAAD and TBAD grouped (Figure 6). The violin plots showed the top 50 divergent metabolites with the highest VIP value (Supplementary Figure S4). The difference in oxylipin species between TAAD and TBAD was significantly greater than that between AD and control.

### 2.4. KEGG Enrichment of Oxylipins

**Figure 7:**
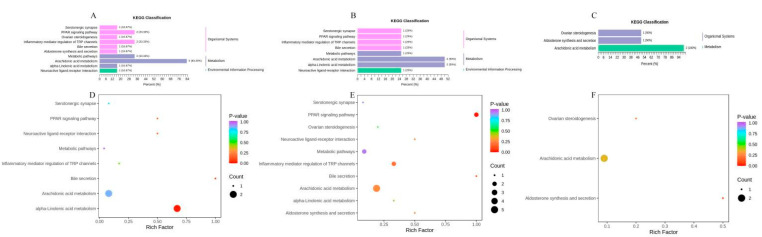
(**A**–**C**) The classification map was created using KEGG annotation findings for a metabolite that differed significantly. KEGG classification map of differential metabolites for Group A versus Group B (**A**), Group A versus Group C (**B**), and Group B versus Group C (**C**). (**D**,**E**) KEGG enrichment of differential metabolites is shown in bubble plots. The abscissa represents the rich factor corresponding to each pathway, the ordinate represents the pathway name, and the color of the point is the *p* value. Bubble plot for Group A and Group B (**D**), Group A versus Group C (**E**), and Group B versus Group C (**F**).

According to the KEGG classification, arachidonic acid metabolism was a significant pathway in all three contrasts (Figure 7A–C). AD patients, particularly TAAD patients, have more distinct oxylipin metabolites involved in arachidonic acid metabolism than healthy people. KEGG enrichment of oxylipins between TAAD and TBAD revealed several routes. The peroxisome proliferators-activated receptors (PPARs) signaling pathway metabolites and arachidonic acid metabolism are likely linked to the difference in metabolic pathways between TAAD and TBAD (Figure 7D–F). There were changes in arachidonic acid metabolism in all three groups, which may play a role in the onset or progression of AD.

According to multivariate logistic regression, 13S-hydroxy-9Z,11E,15Z-octadecatrienoic acid (13-HOTrE) and (±)16,17-epoxy-4Z,7Z,10Z,13Z,19Z-docosapentaenoic acid (16(17)-EpDPE) were influencing factors in the aortic patients group compared to healthy people (OR = 2.467, 95%CI:1.256–7.245, *p* = 0.035; OR = 0.015, 95%CI:0.0002–0.3240, *p* = 0.016, respectively) (Table 2).

### 2.5. ROC Analysis of Oxylipins

**Figure 8:**
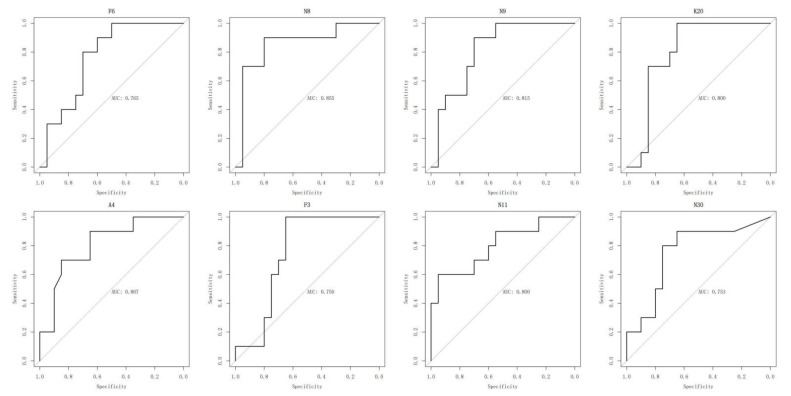
Typical receiver operating characteristic curve (ROC) for prediction of AD patients.

The possible biomarkers were also estimated using receiver operating characteristic (ROC) curve analysis. The area under the ROC curve (AUC), specificity and sensitivity values of some target oxylipins were calculated and are displayed in Figure 8. According to the ROC curves, 9S-hydroxy-10E,12Z,15Z-octadecatrienoic acid (9-HOTrE) has an AUC of 0.855, while eight indicators have an AUC of more than 0.750. ROC curves and AUC values of other indicators are shown in Supplementary Figure S5. The predictive model is established using (±)14(15)-EpETE, 16(17)-EpDPE, DHA, 9-HOTrE and 13-HOTrE, and the AUC is 0.905 (Supplementary Figure S6). Correlation analysis was also conducted and is shown in Supplementary Figure S7.

## 3. Discussion

Aortic dissection is a life-threatening cardiovascular condition that is associated with high mortality and morbidity [27,28]. According to the data, untreated AD has a mortality rate of 1% to 2% per hour from onset. Over half of these patients died within 48 h. Early diagnosis can be problematic in some circumstances, with a 50% probability of misdiagnosis [2,29].

Despite the mechanism and pathophysiology of AD remaining unclear, extracellular matrix degradation and inflammation are key mechanisms of media degeneration [1]. Smooth muscle damage, cardiac stress/damage and thrombosis/fibrinolysis also play important roles in AD [7,30,31]. The smooth muscle myosin heavy chain and calponin can be employed in the identification of AD but cannot provide information about the risk or prognosis of AD [32,33]. Cardiac troponins may distinguish AD from myocardial infarction, but not with high specificity [34]. D-dimer is widely used in clinical practice as an auxiliary diagnosis biomarker, with a sensitivity of 93.5–98% and a specificity of 54–63.2% [4,7]. MMPs reflecting extracellular matrix damage may be beneficial for identifying people at risk of AD [35,36,37]. Current inflammatory biomarkers (C-reactive protein and IL-6) are all not specific for AD diagnosis, while the AUC was 0.700 and 0.750 [38,39,40]. The diagnostic efficiency of these biomarkers is not satisfactory. Although existing inflammatory biomarkers underperform, CRP can predict the prognosis of AD to some extent [41]. As a major factor in the occurrence of AD, it is possible to find more valuable biomarkers in inflammatory response.

Oxylipins play an important role in inflammatory pathways in arthritis, obesity, atherosclerosis and other fields [42,43,44]. Inflammation is one of the key mechanisms of AD. We speculate oxylipins may be potential biomarkers for AD. Here, we established oxylipin profiles that indicated differences in serum oxylipins between healthy individuals and AD patients. Meanwhile, certain altered oxylipins are awaiting additional investigation. To our knowledge, oxylipin profiles in AD have not been reported before our research.

Recent studies have reported various omics in AD serum. Huang et al. reported serum lipidomics in AD and 278 of 439 identified lipid species were significantly altered in two groups [45]. They finally identified lysophosphatidylcholines as excellent potential biomarkers in the diagnosis and treatment of AD. Zhou et al. reported serum metabolomic profiles in acute aortic dissection and identified lysophosphatidylcholines and sphingolipids as new biomarkers [46]. Wang et al. also reported a serum amino acid profile in acute and chronic AD [47]. Serum aminograms were significantly altered in patients with AD compared to coronary heart disease (CHD), especially in acute AD, suggesting the amino acid profile may be a novel, non-invasive, objective diagnosis for AD [48,49,50]. However, the biomarkers used in the current investigation had poor diagnostic efficiency.

Oxylipins, also known as eicosanoids, are signaling molecules derived from enzymatic or non-enzymatic oxidation of ARA or other PUFAs with 20 carbons. Cyclooxygenases enzymes (COX), lipoxygenases enzymes (LOX) or cytochrome P450 epoxygenase (CYP450) are usually the oxidation enzymes for those lipids [51,52]. Different abundances of ARA, LA, ALA, DHA, EPA and DGLA were found in the three groups of our study. The contrast between patients and healthy people was obvious in some of them.

EPA, also called icosapentaenoic acid, is an ω-3 fatty acid. In structure, EPA is a 20-carbon chain carboxylic acid with five cis double bonds [53]. EPA-rich fish oil reduced elastin fiber breakdown in nicotine-administered mice, as well as MMP-12 protein levels, macrophage infiltration, and oxidative stress in the vascular wall. Another study found that EPA can attenuate the increased advancement of abdominal aortic aneurysms (AAA) in Opg-KO mice in a CaCl2-induced AAA model. By activating Gpr-120/Ffar-4, EPA suppresses the Tak-1-JNK pathway, resulting in a reduction in AAA formation [54]. The serum EPA/ARA ratio was 0.44 ± 0.22 in the AAA group, which was lower than that of healthy subjects but equal to that of patients with coronary artery disease [55,56]. The greatest AAA diameter was adversely linked with serum EPA levels and the EPA/AA ratio. Furthermore, EPA levels were found to be a major independent factor in determining the maximum AAA diameter. Takaki et al. reported that EPA may reduce oxidative stress and hence limit the progression of arterial stiffness more effectively than statin-only treatment in individuals with dyslipidemia and coronary artery disease [57]. In our study, a kind of EPA called (±)14(15)-EpETE was significantly lower in the TAAD group compared to healthy individuals. The AUC of (±)14(15)-EpETE, which distinguishes AD from healthy individuals, is 0.855. Additionally, two other types of EPA were shown to be differentially abundant in the TBAD group compared to the TAAD group and the healthy group. These findings suggest that EPA may play an important role in AD.

ARA is a polyunsaturated ω-6 fatty acid with a 20-carbon chain and four cis double bonds in its chemical structure. ARA is found in the phospholipids of cell membranes. In the human body, ARA is always degraded from phospholipids by phospholipase A2 (PLA2) [58]. ARA is converted to both pro-inflammatory and anti-inflammatory eicosanoids during and after an inflammatory response, such as injury, atherosclerosis or ischemia [59]. ARA is also linked to aorta physiology and various aortic diseases. Monika et al. discovered that adipose triglyceride lipase (ATGL) is required for the efficacy of stimulus-induced ARA release and prostacyclin secretion in vascular endothelial cells [60]. A clinical study found that ARA levels in AAA patients were greater than in healthy controls (*p* < 0.001), and those with high ARA levels at baseline had a higher risk of developing AAA [61]. Patients with high ARA levels at baseline showed a 54% increased probability of requiring surgical repair during follow-up. Previous research concentrated on aortic endothelial cells and aortic smooth muscle cells [62,63]. Despite the focus on aortic cells, there are very few studies about aortic dissection or aneurysm. In our investigation, about 38 kinds of ARA were detected in AD and healthy individuals. In the KEGG enrichment bubble diagram, the arachidonic acid metabolism pathway was selected as a high risk factor, showing ARA may play an important role in AD development. The AUC values of 5,6-epoxy-8Z,11Z,14Z-eicosatrienoic acid (5,6-EET) and 12-Hydroxy-5,8,10-heptadecatrienoic acid (12-HHT) are both greater than 0.800.

Docosahexaenoic acid (DHA) is a 22-carbon chain ω-3 fatty acid containing six cis double bonds [64]. DHA is the most abundant fatty acid found in brain phospholipids and the retina. DHA is being studied for its possible benefit in cardiovascular disease [65]. However, few studies have focused on aortic disease. Intermediate conductance Ca^2+^—activated K+ channels (IKCa) and large conductance Ca^2+^—activated K+ channels (BKCa) are implicated in DHA-induced relaxation in the aorta and mesenteric artery of rats, but not in EPA-induced relaxation in the mesenteric artery of rats, according to Limbu et al. [66]. EPA metabolites generated from CYP450 may potentially be implicated in BKCa-dependent relaxation. In another study, Otsuka, K. discovered that DHA inhibits TXA2 receptor-mediated vascular contraction more selectively than α-adrenoceptor-mediated response in the aorta of guinea-pigs [67]. Boivin et al. reported that DHA infusion improved artery contractile response to phenylephrine through nitric oxide pathway inhibition. In septic shock-induced arterial dysfunction, DHA can also considerably lower vascular oxidative stress and vasodilative prostaglandin synthesis. Yoshihara et al. discovered that dietary EPA and DHA consumption can prevent AAA formation by inhibiting aortic and macrophage-mediated inflammation [68]. According to recent studies, replenishment with EPA and DHA can significantly reduce the expression of TNF-α, TGF-β, MCP-1, MMP-2, MMP-9 and VCAM-1 in the aorta [69,70]. These studies suggest that DHA has a protective function in several types of aortic disease, as well as therapeutic promise in AD. In our study, DHA was significantly different between the AD groups and healthy controls with an AUC of 0.750.

## 4. Materials and Methods

### 4.1. Patients

The study comprised 10 patients with TAAD (Group A) and 10 patients with TBAD (Group B) who were treated at the Department of Thoracic and Cardiovascular Surgery, at the Affiliated Drum Tower Hospital of Nanjing University Medical School. A total of 10 healthy controls were gathered from the medical examination facility. People with the following conditions were excluded: atherosclerotic diseases, aneurysms, valvular diseases, malignant tumors, pregnancy, immune diseases, acute illness, end-stage organ failure and any other condition that may affect the results. All patients were diagnosed by computed tomography angiography (CTA). The Ethical Review Committee of Nanjing Drum Tower Hospital (2020-073) approved the study and all patients submitted written informed consent.

### 4.2. Sample Collection and Preparation for Oxylipin Analysis

We collected a 3.5 mL blood sample in BD Vacutainer Collection Tubes (Melbourne, Australia) containing spray-coated silica and a polymer gel for serum separation then immediately placed them on ice. After centrifuging the tubes at 3000 rpm for 5 min at 4 °C in a chilled centrifuge, the serum was aliquoted into 1.5 mL Eppendorf tubes (Shanghai, China) and stored at −80°C until analysis. Keeping the serum away from light throughout the collecting and transfer procedure is crucial.

### 4.3. Detection of Oxylipins

We established a liquid chromatography with tandem mass spectrometry (LC-MS/MS) platform for the quantitative detection of oxylipins, including downstream oxidized metabolites from α-linolenic acid (ALA), ARA, DHA, EPA and DGLA. Corresponding abbreviations are shown in Supplementary Table S1.

Due to low concentration, the large number of isomers and the ease of oxidation, a thorough analytical approach for oxylipins consists of three steps: extraction, enrichment, and detection. Briefly, 100 μL of sample was spiked with 200 μL of oxylipin extract, vortexed for 5 min, and the protein was then precipitated at −20 °C. The samples were then spiked with 20 μL of 1 μmol/L internal standard mixture and vortexed for 10 min, centrifuged at 5000 rpm for 10 min at 4 °C, and the supernatants were then combined. Poly-Sery MAX SPE columns were used to extract eicosanoids from supernatants. The eluent was vacuum-dried before being redissolved in 100 μL of methanol/water (1:1, *v*/*v*) for UPLC/MS/MS analysis. The mass spectrometry data were processed using Analyst 1.6.3 and MultiQuant 3.0.3 software.

### 4.4. Data Acquisition and Processing

The statistics function prcomp in the “stats” package (version 4.1.2) was used to perform unsupervised PCA in R software (version 4.1.2). Before unsupervised PCA, the data was unit variance scaled. HCA was performed using the R package “pheatmap” (version 1.0.12) to display heatmaps with dendrograms, while the Pearson correlation coefficients (PCC) between samples were obtained using the cor function and displayed as heatmaps. Normalized metabolite signal intensities are displayed as a color spectrum for HCA. Variable importance (VIP) in projection was used to assess which metabolites were significantly altered between groups. In order to avoid over-fitting, a permutation test (200 permutations) was performed. Correlation was created using the R packages “Hmisc” (version 4.6-0) and “corrplot” (version 0.92). Violin plot was created using the R package “Vioplot” (version 0.3.7).

The KEGG compound database was used to annotate the identified metabolites, and the annotated metabolites were then linked to the KEGG Pathway database. Metabolite sets enrichment analysis (MSEA) was then loaded into the pathways with substantially regulated metabolites.

### 4.5. Statistical Analysis

All data were presented using n (%) as categorical variables and mean±standard deviation as a continuous variable with a normal distribution. The Kolmogorov–Smirnov test was used to determine the normality distribution. Analysis of variance (ANOVA) or Student’s t-test was used for variables with normally distributed variables. The categorical variables were properly examined using the Fisher’s exact test. Table 1 for basic statistics was created using the R package “tableone” (version 0.13.0). Predictive quality was evaluated using the receiver operating characteristic curve (ROC) and the area under the ROC curve (AUC) using the “pROC” package (version 1.18.0). Statistical significance was determined when *p* values were less than 0.05.

### 4.6. Limitations of the Study

This study still has some limitations. First, it was a retrospective study at a single center with a bias of disease type or region. Second, each set of research has a small sample size, and it is a cross-sectional study which might be unduly conservative in clinical significance interpretation.

## 5. Conclusions

To our knowledge, this is the first study of oxylipins in AD. We discovered that oxylipins were significantly altered in blood samples from AD patients and healthy controls. Our investigation demonstrated substantial alterations in DHA, EPA and ARA. The oxylipin profiles may provide new information and assistance for the diagnosis and prognosis of AD. Finally, we established an early diagnostic model with an auc of 0.905.

## Figures and Tables

**Table 1:** Demographic characteristics and baseline data of patients with aortic dissection and healthy controls.

	TAAD (*n* = 10)	TBAD (*n* = 10)	CONTROL (*n* = 10)	
	Group A	Group B	Group C	*p*
Age (years)	47.40 ± 12.77	52.20 ± 13.05	49.10 ± 4.91	0.790
Gender (%)				
Male (*n*)	10 (100.0)	7 (70.0)	7 (70.0)	0.153
Female (*n*)	0 (0.0)	3 (30.0)	3 (30.0)	
BMI (kg/m2)	28.09 ± 6.26	27.78 ± 9.04	24.81 ± 3.73	0.491
Hypertension (%)	6 (60.0)	7 (70.0)	9 (90.0)	0.303
Diabetes (%)	0 (0.0)	2 (20.0)	1 (10.0)	0.329
WBC (109/L)	9.90 [7.50, 15.68]	11.85 [7.82, 13.38]	6.05 [5.45, 6.47]	0.003
NEU percentage (%)	86.65 [80.00, 88.35]	85.40 [81.55, 91.10]	59.50 [56.60, 64.20]	<0.001
RBC (1012/L)	4.31 [3.47, 4.68]	4.78 [3.97, 5.05]	4.90 [4.65, 5.12]	0.046
Hemoglobin (g/L)	139.50 [109.25, 144.00]	146.00 [117.75, 147.00]	156.50 [149.25, 163.25]	0.010
Platelet (109/L)	153.00 [111.50, 184.00]	155.50 [120.00, 235.25]	237.50 [198.25, 266.75]	0.010
GPT (U/L)	24.80 [21.00, 28.00]	15.25 [14.03, 19.75]	26.30 [18.38, 32.27]	0.159
GOT (U/L)	28.00 [21.00, 41.40]	21.15 [17.80, 31.42]	22.95 [19.73, 24.80]	0.342
ALP (U/L)	62.60 [55.48, 67.03]	76.10 [65.10, 88.80]	62.40 [54.15, 74.53]	0.173
LDH (U/L)	262.00 [194.00, 366.00]	251.00 [187.00, 270.25]	179.00 [175.25, 186.00]	0.025
Total bilirubin (μmol/L)	17.15 [9.27, 25.25]	14.50 [10.50, 16.65]	12.75 [10.15, 16.52]	0.753
Direct bilirubin (μmol/L)	3.05 [2.72, 4.47]	3.70 [3.10, 4.20]	2.10 [1.75, 2.80]	0.009
Cholinesterase (KU/L)	7.20 [6.07, 8.27]	7.40 [6.10, 8.90]	9.85 [9.50, 10.83]	0.004
Total protein (g/L)	65.90 [47.40, 67.50]	70.55 [65.80, 72.95]	75.70 [73.95, 77.85]	<0.001
Albumin (g/L)	38.60 [31.70, 41.10]	42.35 [41.85, 43.38]	45.80 [45.12, 46.65]	<0.001
Globulin (g/L)	24.80 [19.30, 27.80]	27.75 [24.70, 31.63]	29.80 [28.33, 32.73]	0.012
A/G Rate	1.39 [1.36, 1.73]	1.50 [1.32, 1.72]	1.52 [1.39, 1.62]	0.993
Urea (mmol/L)	5.81 [5.15, 6.40]	6.40 [5.00, 7.83]	4.90 [4.12, 5.47]	0.166
Creatinine (μmol/L)	77.95 [63.25, 90.17]	71.50 [59.40, 85.00]	65.50 [60.00, 74.50]	0.253
Uric acid (μmol/L)	396.00 [319.00, 445.25]	322.50 [265.25, 466.50]	389.50 [318.50, 443.50]	0.830
Triglycerides (mmol/L)	1.07 [0.81, 1.65]	0.56 [0.44, 1.14]	2.08 [1.52, 2.89]	0.007
cholesterol (mmol/L)	3.81 [3.38, 4.02]	4.48 [3.94, 4.89]	5.79 [5.41, 6.69]	0.001
H-cholesterol (mmol/L)	1.00 [0.95, 1.04]	1.14 [0.95, 1.56]	1.06 [1.02, 1.55]	0.612
L-cholesterol (mmol/L)	2.18 [1.89, 2.30]	2.74 [2.51, 2.94]	3.49 [3.42, 4.31]	0.006
Apo AI (g/L)	0.98 [0.86, 1.04]	1.03 [0.93, 1.15]	1.08 [1.07, 1.09]	0.372
Apo B (g/L)	0.70 [0.61, 0.72]	0.81 [0.78, 0.92]	0.76 [0.58, 0.93]	0.335
eGFR	77.00 [51.80, 102.20]	106.60 [75.00, 119.30]	108.35 [99.12, 125.17]	0.369
CRP (mg/L)	7.50 [2.47, 31.00]	6.70 [3.90, 17.40]	1.55 [1.52, 1.58]	0.302
CK (U/L)	116.00 [82.00, 267.00]	76.00 [43.00, 111.00]	—	0.093
CKMB (U/L)	8.00 [3.00, 17.00]	6.00 [4.00, 12.00]	—	0.789
cTNT (ug/L)	0.03 [0.01, 0.04]	0.01 [0.01, 0.02]	—	0.289
D-Dimer (μg/mL)	4.14 [3.62, 8.04]	4.22 [1.67, 8.08]	—	0.929
BNP (pg/mL)	35.90 [23.60, 56.70]	65.10 [27.77, 115.70]	—	0.431
PCT (ng/mL)	0.70 [0.37, 22.24]	0.04 [0.04, 0.05]	—	0.248

BMI, Body Mass Index; WBC, white blood cell; Neu, Neutrophil count; RBC, red blood cell; GPT, glutamic-pyruvic transaminase; ALP, alkaline phosphatase; Apo, apolipoprotein; eGFR, estimated glomerular filtration rate; CRP, C-reactive protein; CK, creatine kinase; cTNT, cardiac troponin T; BNP, Brain natriuretic peptide; PCT, procalcitonin.

**Table 2:** Multivariate logistic regression screened characteristic variables.

Variable Name	OR	2.5% CI	97.5% CI	B	Wald	*p* Value
N9	2.467	1.256	7.245	0.903	4.454	0.035
A4	0.872	0.734	0.974	−0.137	4.001	0.045
F3	1.000	1.000	1.000	0.000	4.764	0.029
N11	0.015	0.000	0.324	−4.231	5.761	0.016
N30	0.003	0.000	0.613	−5.986	3.943	0.047

OR, odd ratio; CI, confidence interval; B, regression coefficient.

## Data Availability

The data presented in this study are available on request from the corresponding author. The data are not publicly available due to ethical reasons.

## Supplementary Materials

The following supporting information can be downloaded at: https://www.mdpi.com/article/10.3390/metabo12070587/s1, Figure S1: Data preprocessing and evaluation; Figure S2: Diverse patterns of Heatmap; Figure S3: OPLS-DA S-plots; Figure S4: Violin plots; Figure S5: ROC curve of other oxylipins; Figure S6: The AUC of the five-index model was 0.905; Figure S7: Correlation analysis between different oxylipins; Table S1: Table of abbreviations and full names of oxylipins.

## Abbreviations

AAA	Abdominal Aortic Aneurysm;
ACS	Acute Coronary Syndromes;
AD	Aortic Dissection;
ALA	α-Linolenic Acid;
ANOVA	Analysis of Variance;
ARA	Arachidonic Acid;
ATGL	Adipose Triglyceride Lipase;
AUC	Area under the ROC Curve;
BKCa	Large conductance Ca^2+^—activated K+ channels;
BMI	Body Mass Index;
CHD	Coronary Heart Disease;
COX	Cyclooxygenase;
CT	Computed Tomography;
CTA	Computed Tomography Angiography;
CYP450	Cytochrome P450;
DGLA	Dihomo-γ-Linolenic Acid;
DHA	Docosahexaenoic Acid;
EPA	Eicosapentaenoic Acid;
GLA	γ-Linolenic Acid;
HCA	Hierarchical Cluster Analysis;
IKCa	Intermediate conductance Ca^2+^—activated K+ channels;
IL-6	Interleukin-6;
KEGG	Kyoto Encyclopedia of Genes and Genomes;
LA	Linolenic Acid;
LC-MS/MS	Liquid Chromatography Linked to Tandem Mass Spectrometry;
LOX	Lipoxygenase;
OPLS-DA	Orthogonal Partial Least Squares-Discriminant Analysis;
PCA	Principal Component Analysis;
PPARs	Peroxisome Proliferators-Activated Receptors;
PUFA	Polyunsaturated Fatty Acid;
ROC	Receiver Operating Characteristic Curve;
TAAD	Type A Aortic Dissection;
TBAD	Type B Aortic Dissection;
TIC	Total Ion Chromatogram;
VIP	Variable Importance in Projection;
XIC	Extracted Ion Chromatogram.
