# Supplementary material for: Serum Oxylipin Profiles Identify Potential Biomarkers in Patients with Acute Aortic Dissection
Source: Metabolites. 2022 Jun 23;12(7):587. doi: 10.3390/metabo12070587 (PMC9324768; doi:10.3390/metabo12070587)
Supplement: Supplementary file 1 [file metabolites-12-00587-s001.zip › Supplementary Figure S4.pdf]

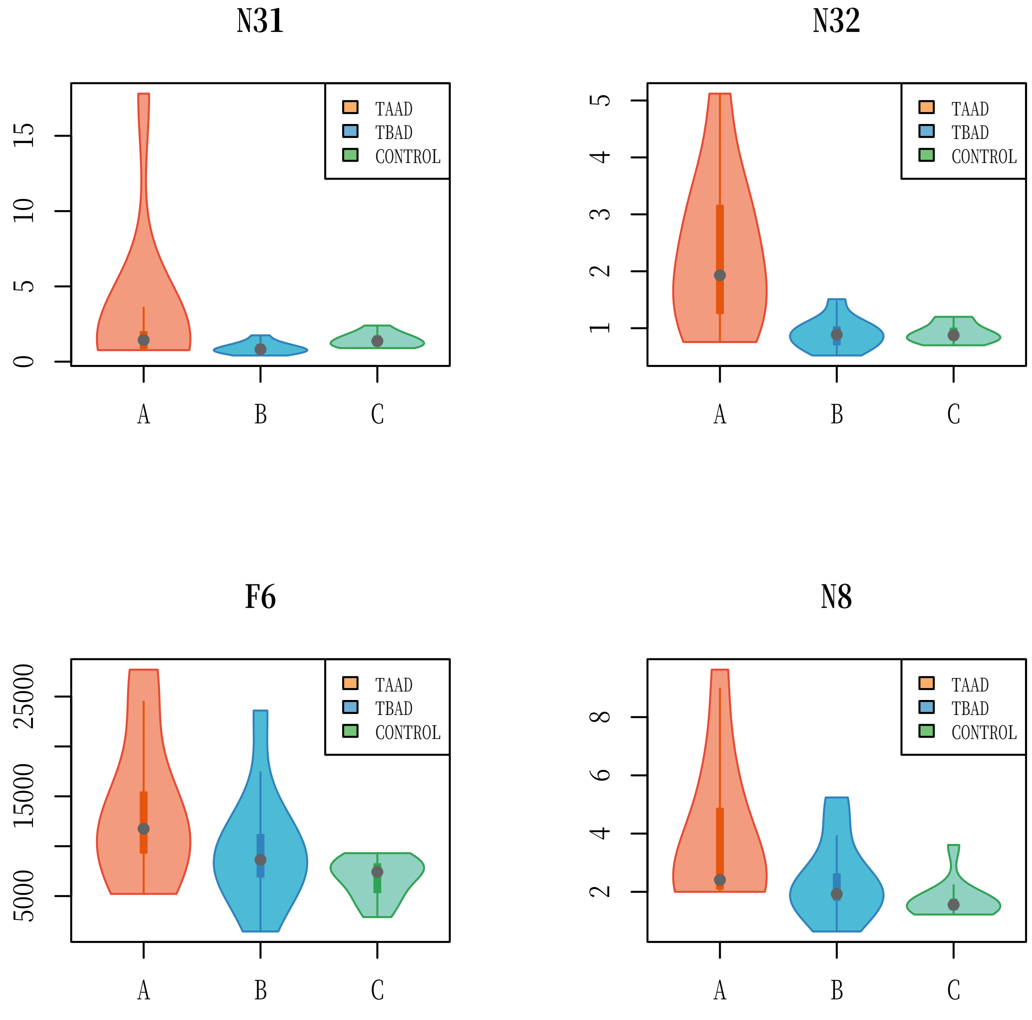

N31
N32
F6
N8
TAAD
TBAD
CONTROL
A
B
C
0
5
10
15
1
2
3
4
5
5000
15000
25000
2
4
6
8

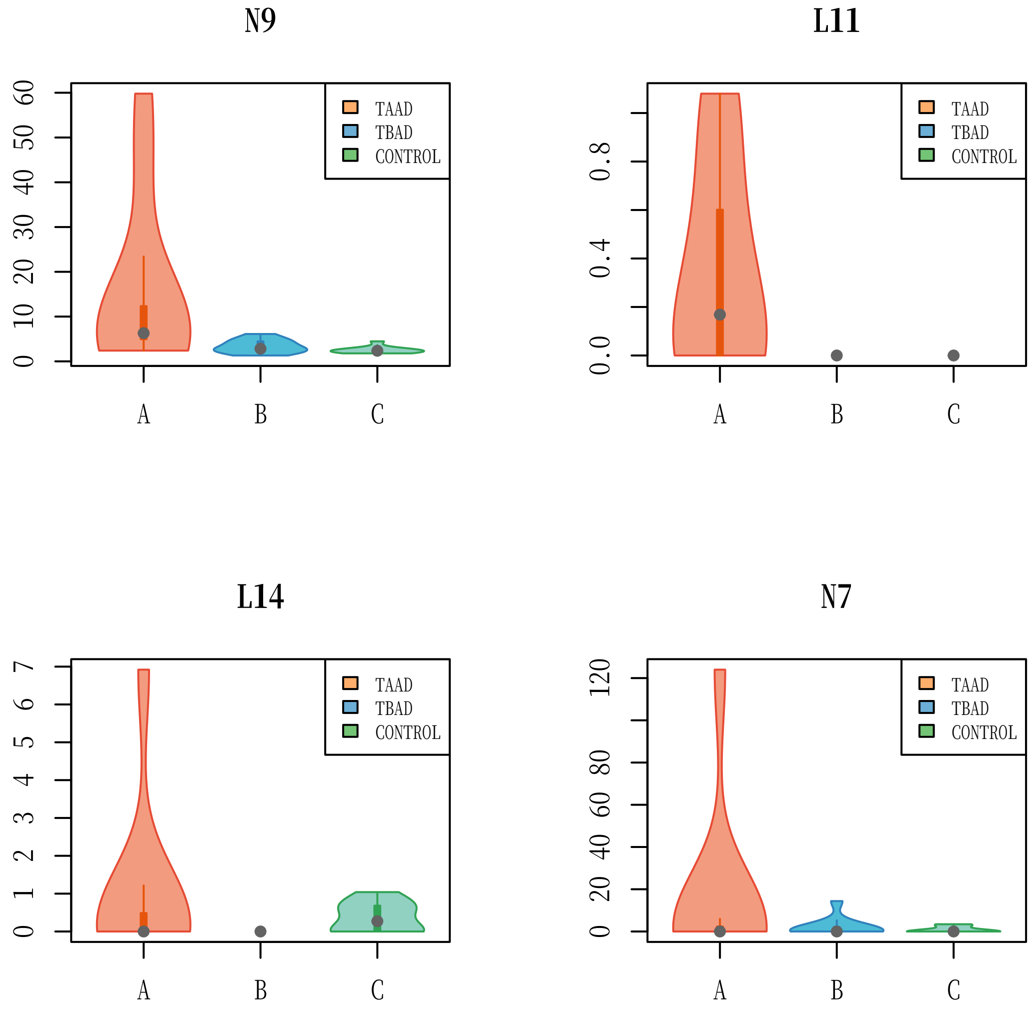
N9
L11
L14
N7
TAAD
TBAD
CONTROL
A
B
C
0
10
20
30
40
50
60
0.0
0.4
0.8
1
2
3
4
5
6
7
20
40
60
80
120

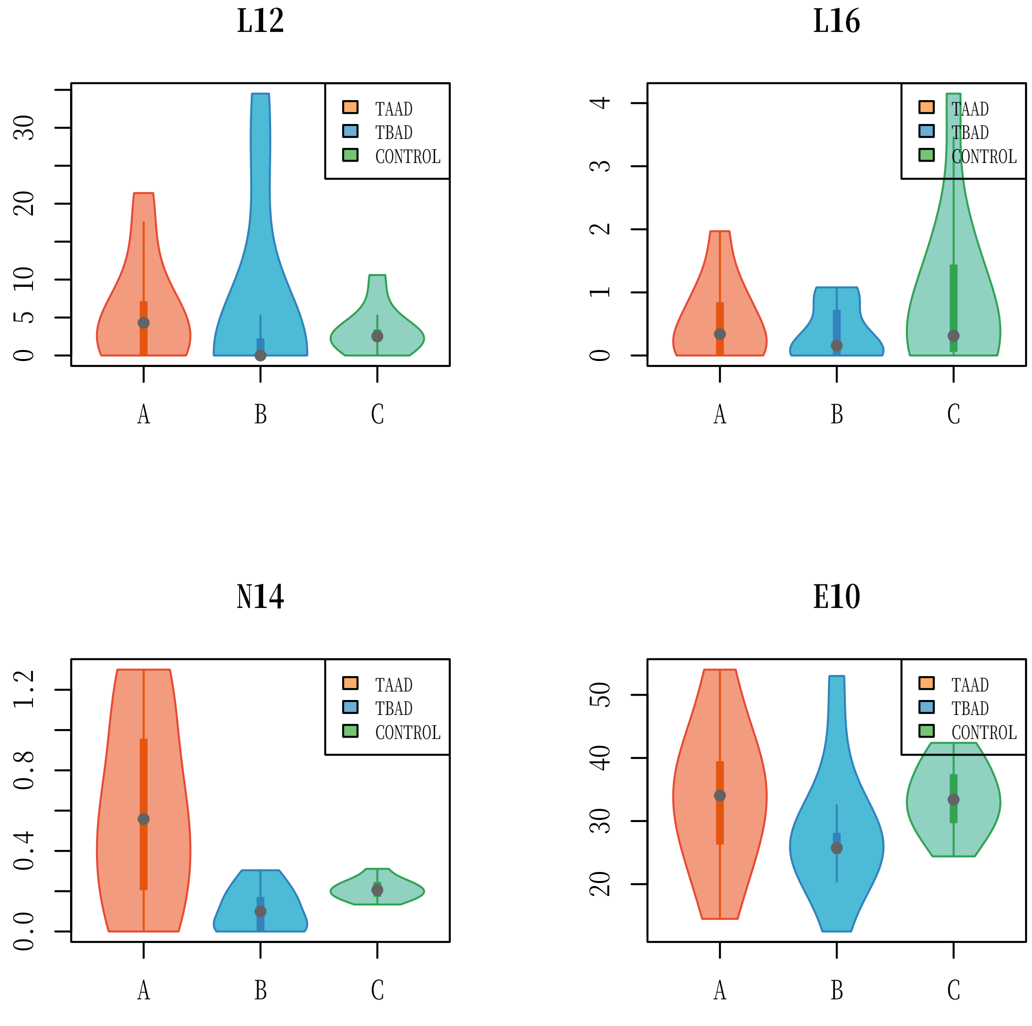
L12
L16
N14
E10
TAAD
TBAD
CONTROL
A
B
C
0
5
10
20
30
1
2
3
4
0.0
0.4
0.8
1.2
40
50

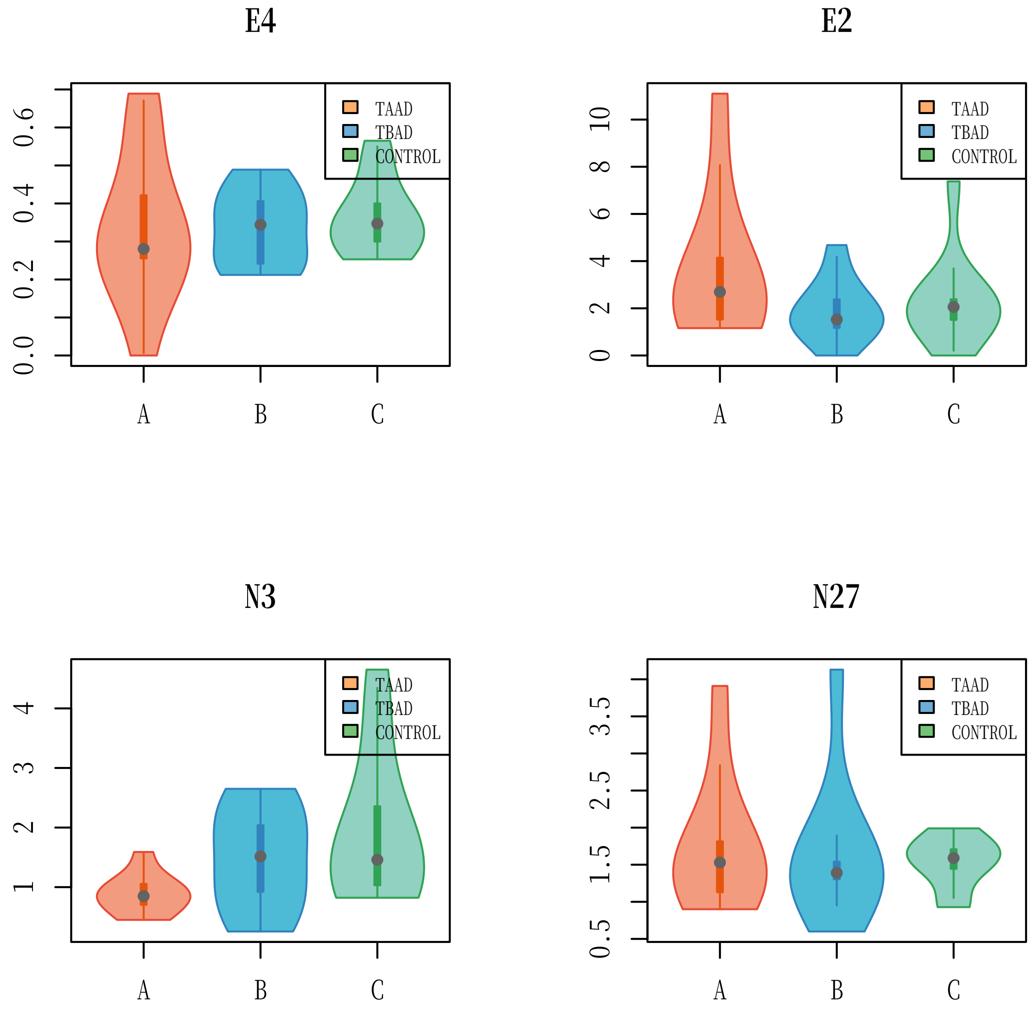
E4
E2
N3
N27
TAAD
TBAD
CONTROL
A
B
C
0.0
0.2
0.4
0.6
0
2
4
6
8
10
1
2
3
4
0.5
1.5
2.5
3.5

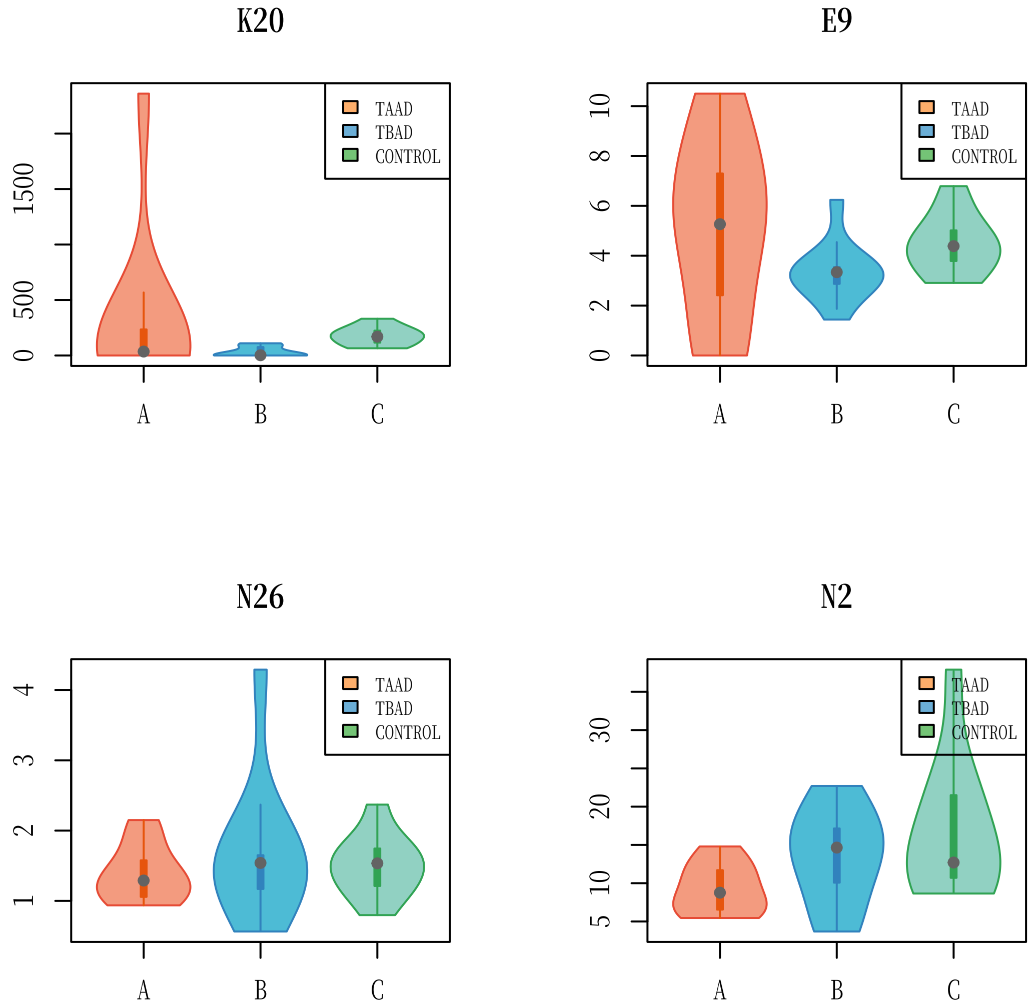
K20
1500
500
0
TAAD
TBAD
CONTROL
A
B
C
E9
10
8
6
4
2
0
TAAD
TBAD
CONTROL
A
B
C
N26
4
3
2
1
TAAD
TBAD
CONTROL
A
B
C
N2
30
20
10
5
TAAD
TBAD
CONTROL
A
B
C

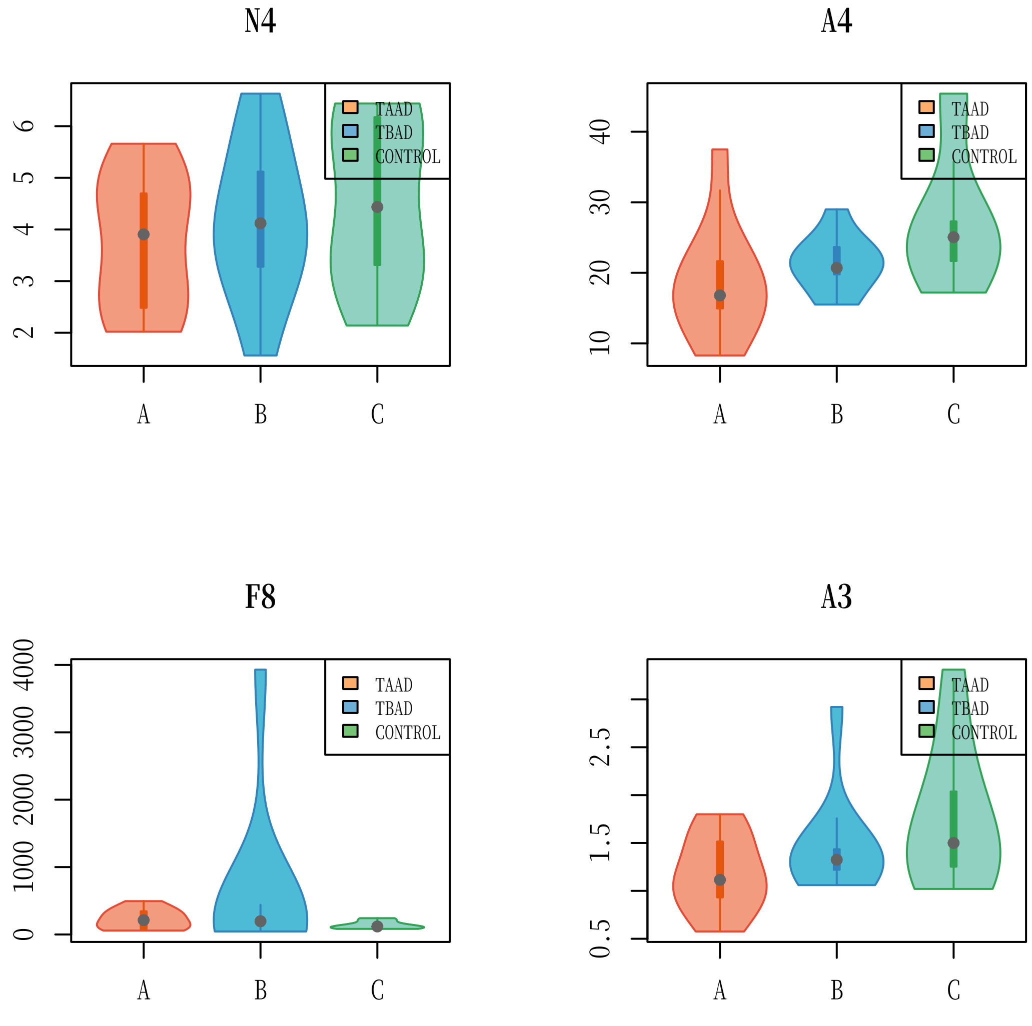
N4
A4
F8
A3
TAAD
TBAD
CONTROL
A
B
C
2
3
4
5
6
10
20
30
40
0
1000
2000
3000
4000
0.5
1.5
2.5

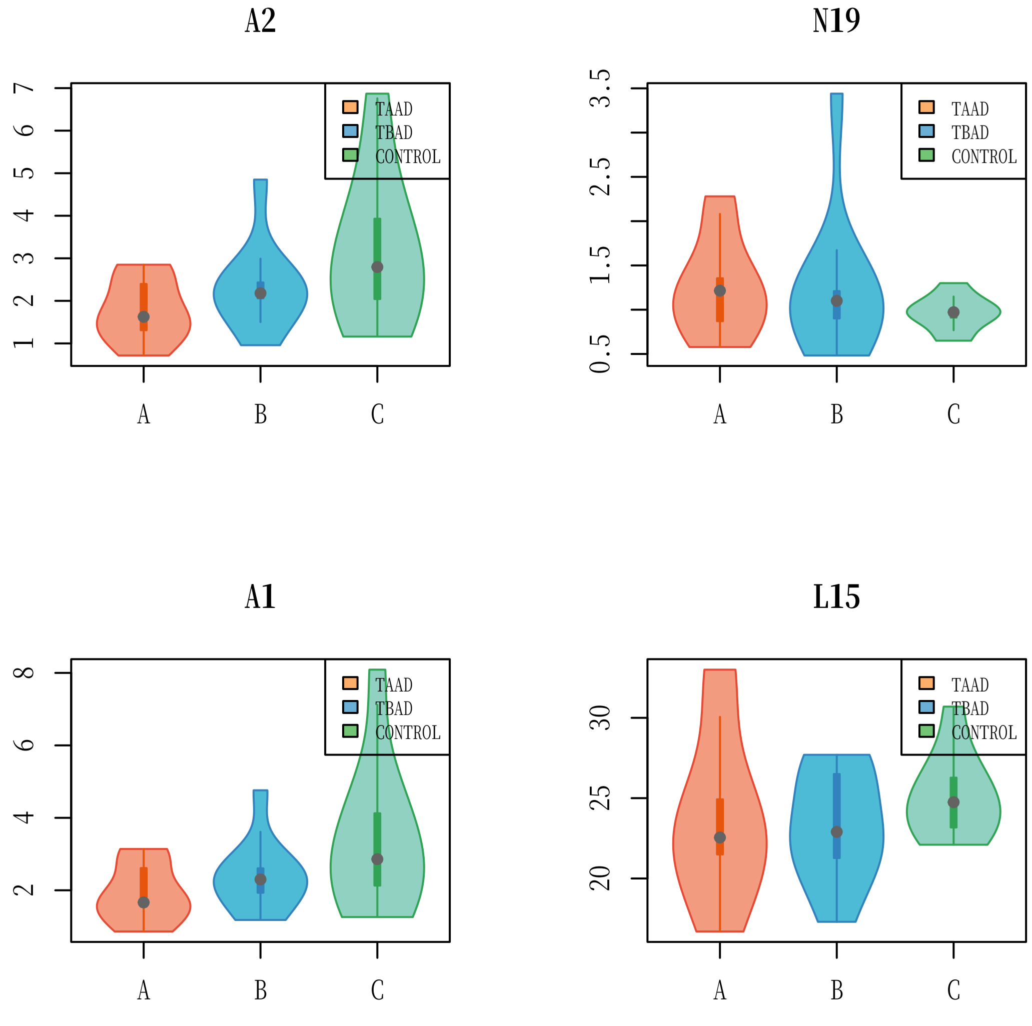
A2
N19
A1
L15
TAAD
TBAD
CONTROL
A
B
C
1
2
3
4
5
6
7
0.5
1.5
2.5
3.5
8
20
25
30

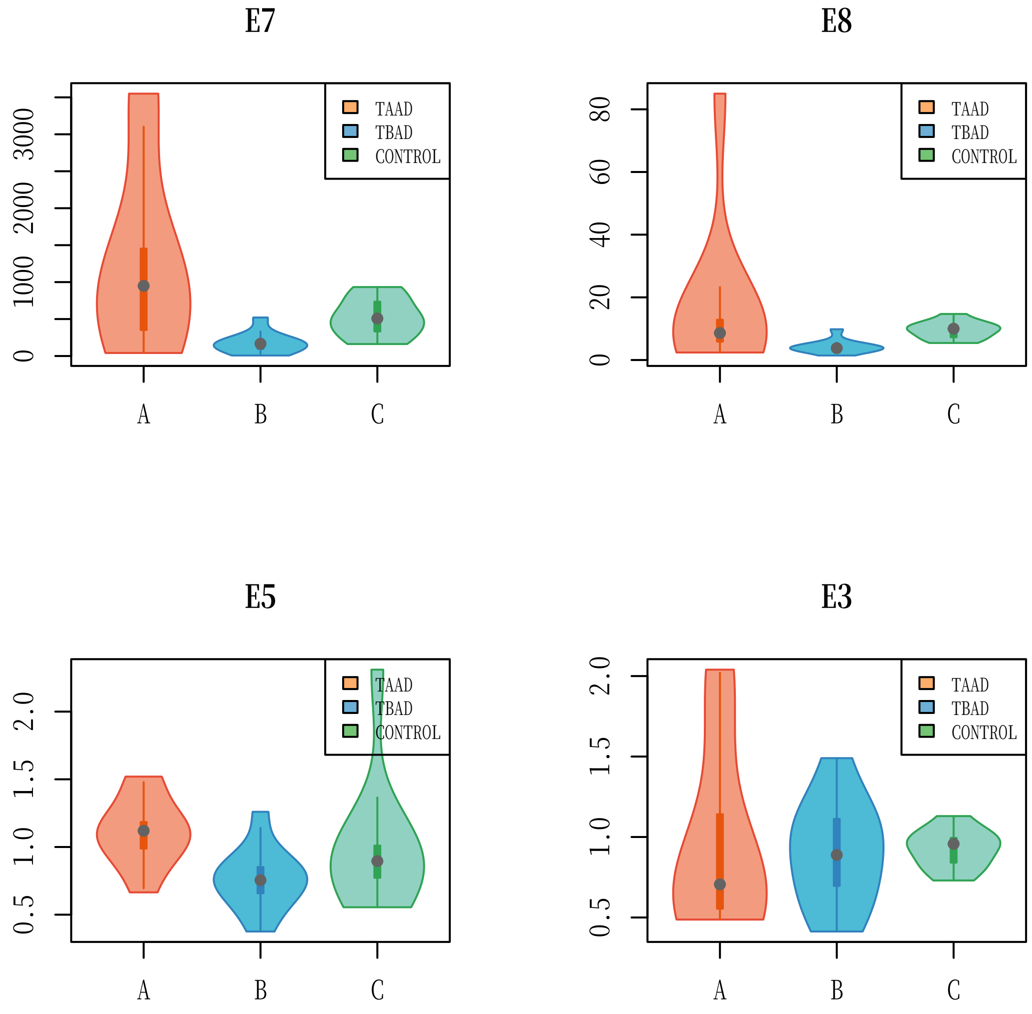
E7
0
1000
2000
3000
TAAD
TBAD
CONTROL
A
B
C
E8
0
20
40
60
80
TAAD
TBAD
CONTROL
A
B
C
E5
0.5
1.0
1.5
2.0
TAAD
TBAD
CONTROL
A
B
C
E3
0.5
1.0
1.5
2.0
TAAD
TBAD
CONTROL
A
B
C

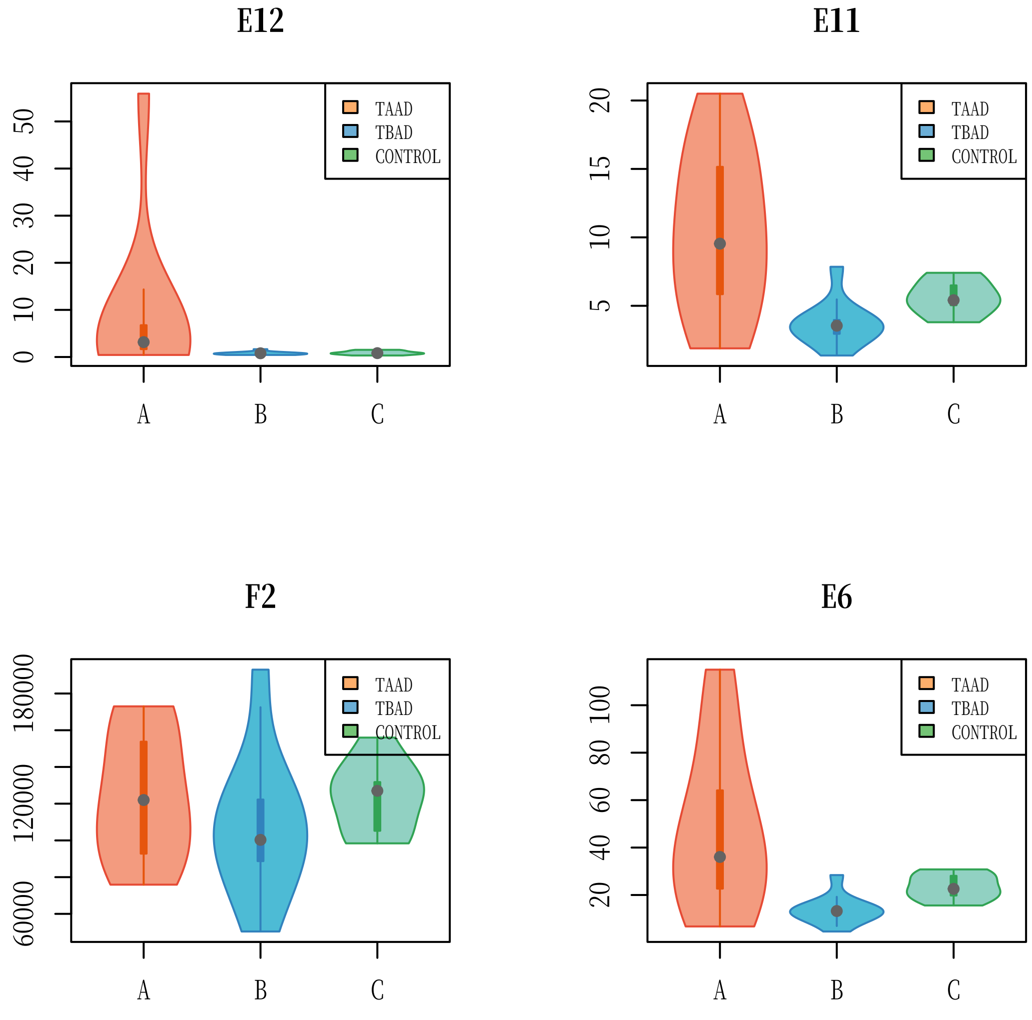
E12
0
10
20
30
40
50
A
B
C
TAAD
TBAD
CONTROL
E11
5
10
15
20
A
B
C
TAAD
TBAD
CONTROL
F2
60000
120000
180000
A
B
C
TAAD
TBAD
CONTROL
E6
20
40
60
80
100
A
B
C
TAAD
TBAD
CONTROL

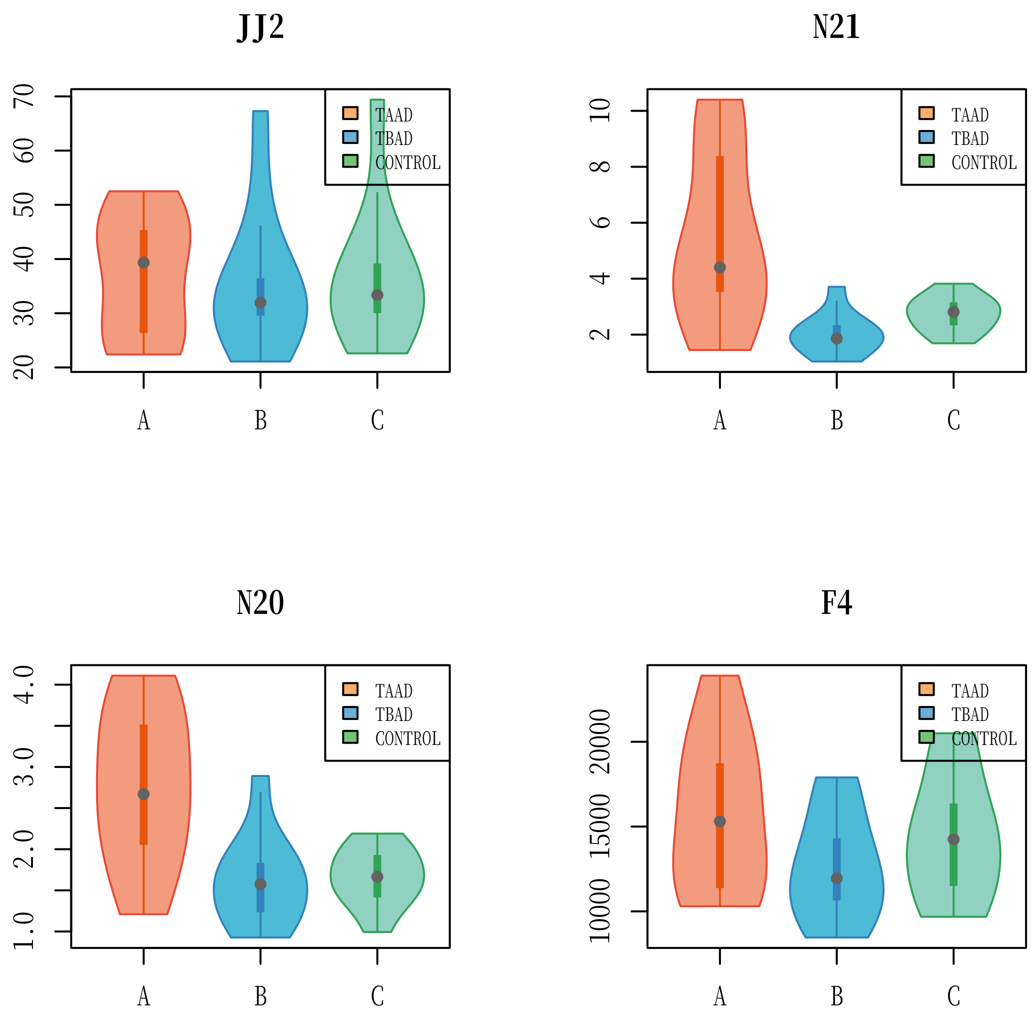
JJ2
N21
N20
F4
TAAD
TBAD
CONTROL
A
B
C
20
30
40
50
60
70
2
4
6
8
10
1.0
2.0
3.0
4.0
10000
15000
20000

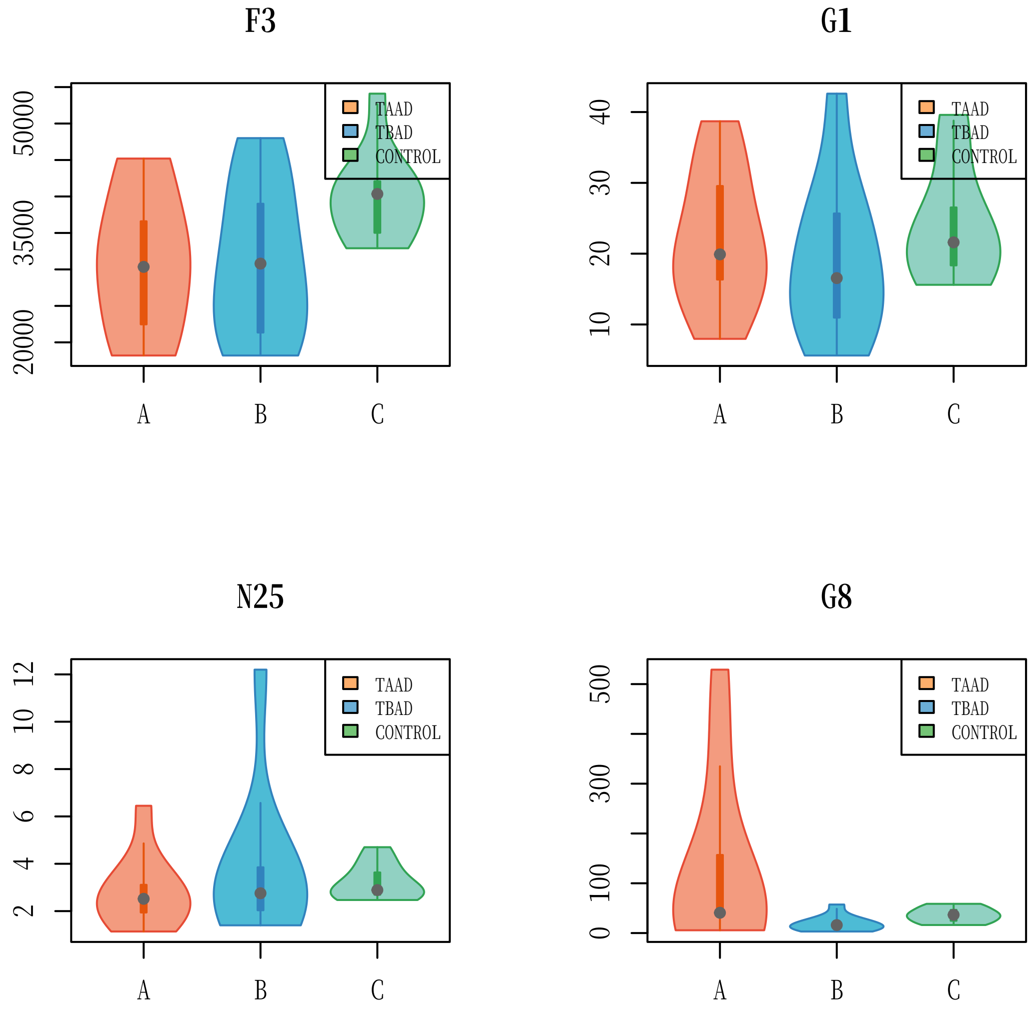
F3
G1
N25
G8
TAAD
TBAD
CONTROL
A
B
C
20000
35000
50000
10
20
30
40
2
4
6
8
10
12
0
100
300
500

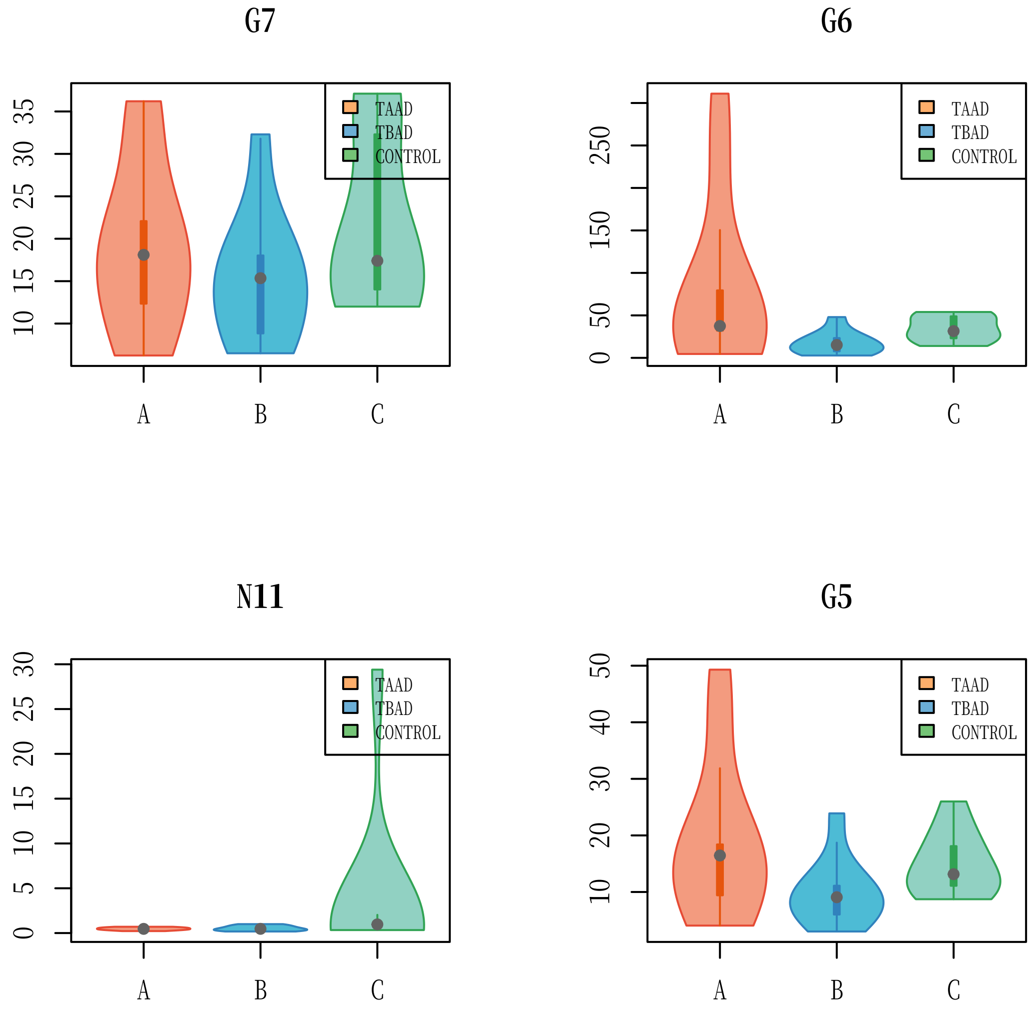
G7
G6
N11
G5
TAAD
TBAD
CONTROL
A
B
C
0
5
10
15
20
25
30
35
40
50
150
250

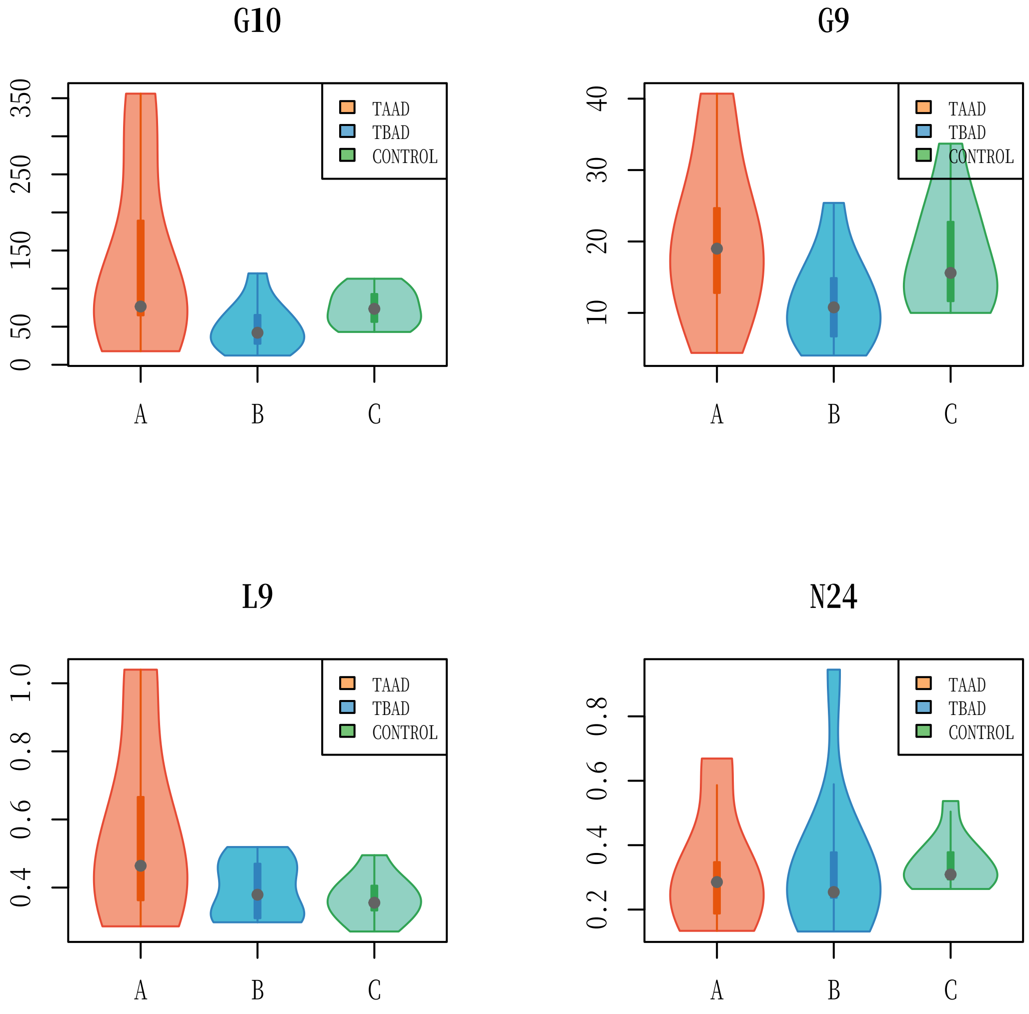
G10
G9
L9
N24
TAAD
TBAD
CONTROL
A
B
C
0
50
150
250
350
10
20
30
40
0.4
0.6
0.8
1.0
0.2

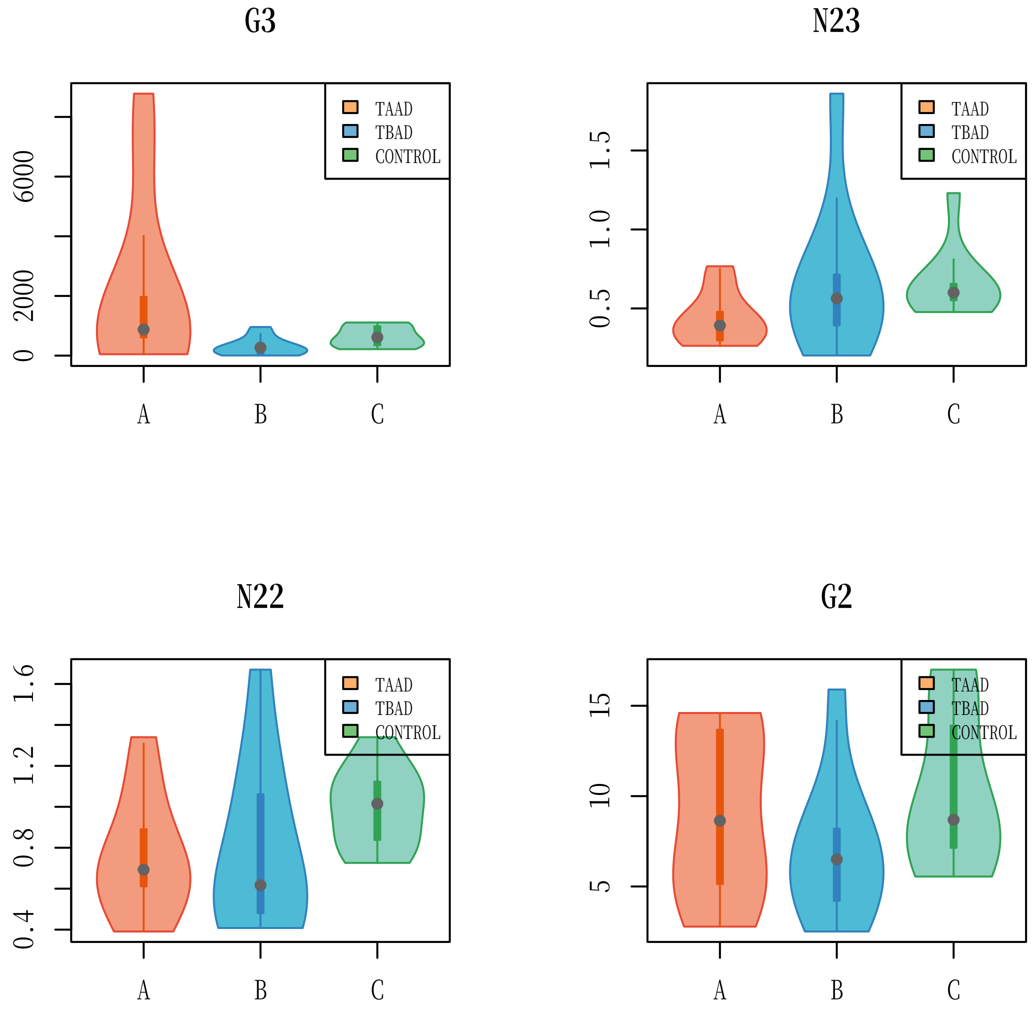
G3
N23
N22
G2
TAAD
TBAD
CONTROL
A
B
C
0
2000
6000
0.5
1.0
1.5
0.4
0.8
1.2
1.6
5
10
15

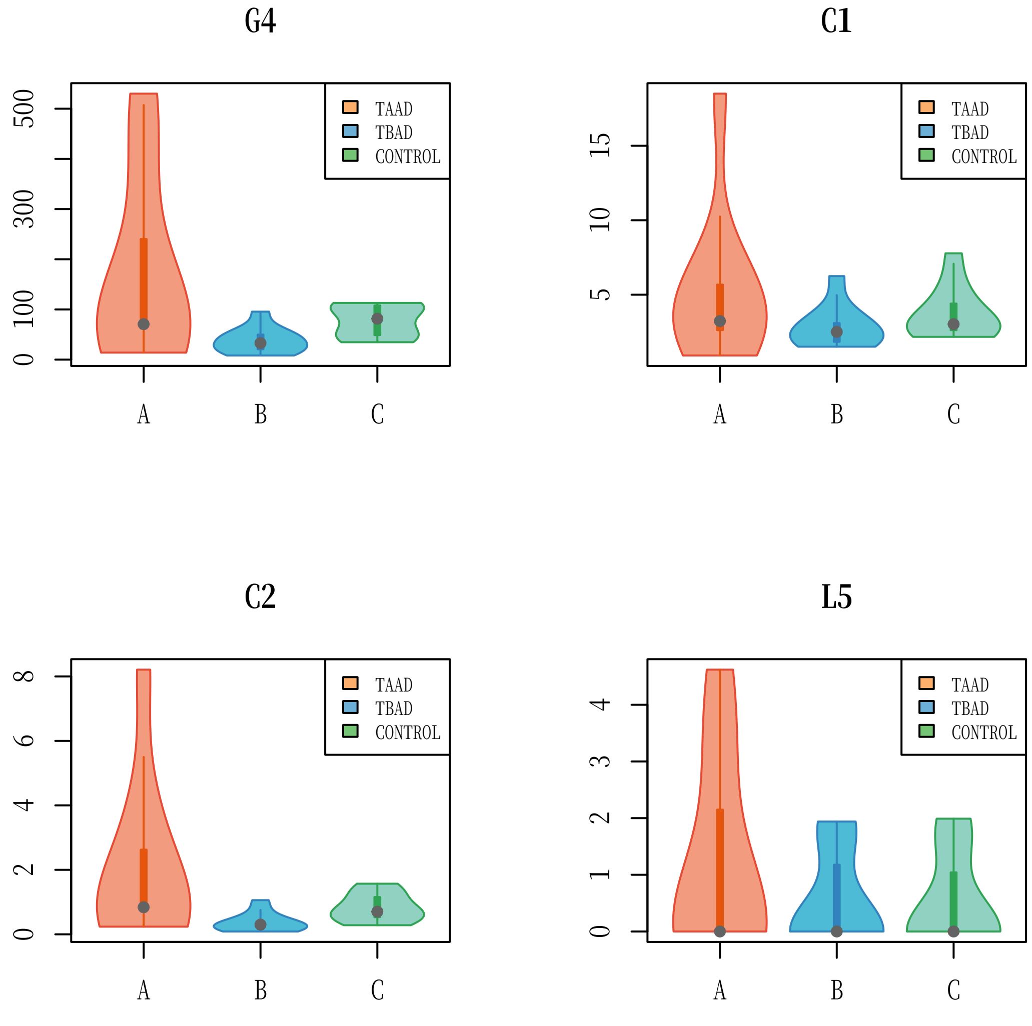

G4
C1
C2
L5
TAAD
TBAD
CONTROL
A
B
C
0
100
300
500
5
10
15
2
4
6
8
1
3

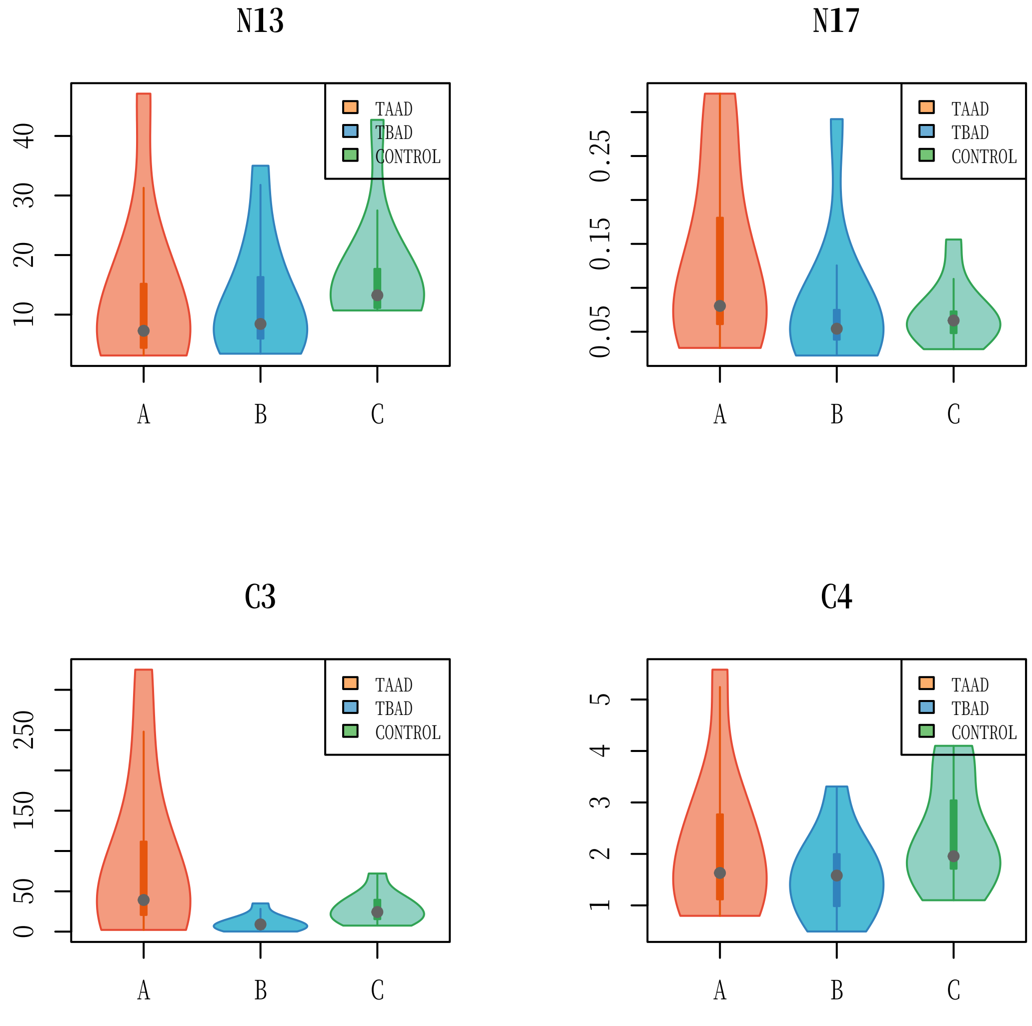
N13
N17
C3
C4
TAAD
TBAD
CONTROL
A
B
C
10
20
30
40
0.05
0.15
0.25
0
50
150
250
1
2
3
4
5

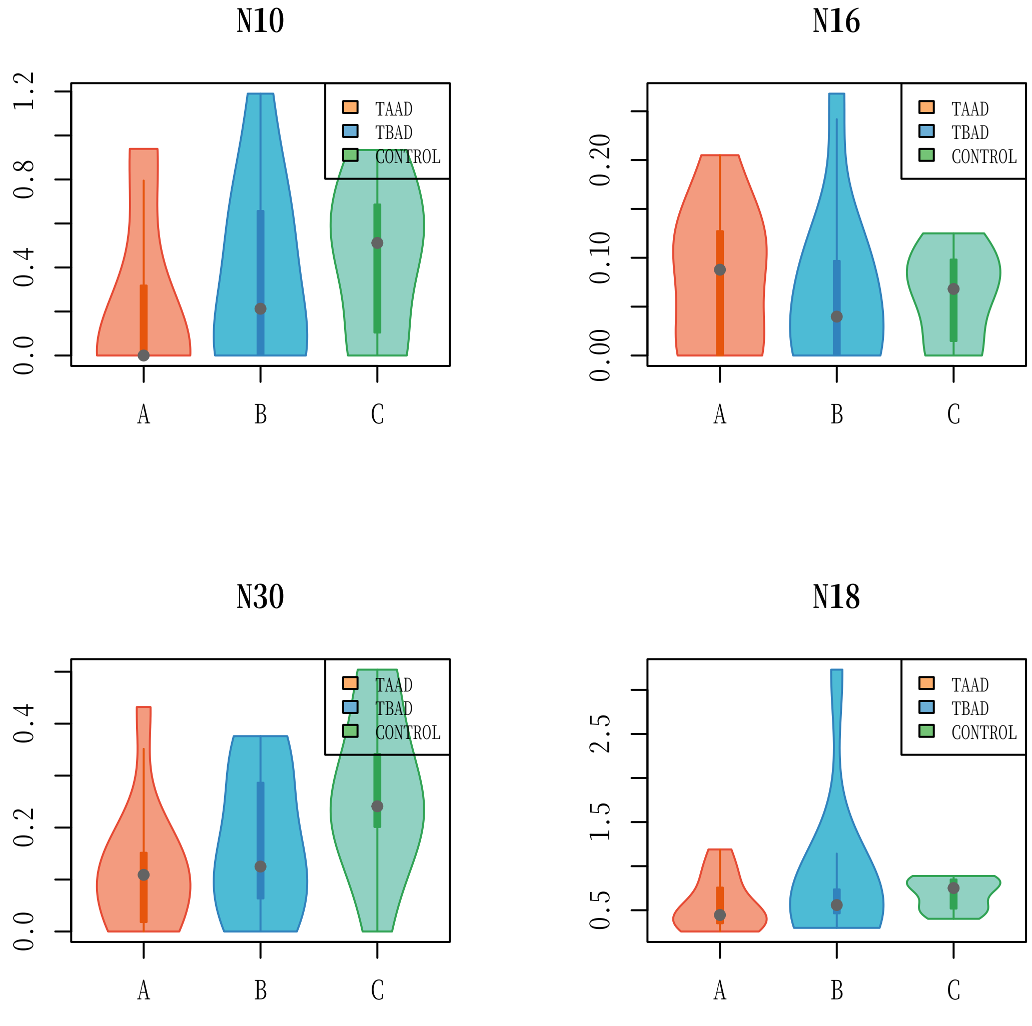
N10
TAAD
TBAD
CONTROL
0.0
0.4
0.8
1.2
A
B
C
N16
TAAD
TBAD
CONTROL
0.00
0.10
0.20
A
B
C
N30
TAAD
TBAD
CONTROL
0.0
0.2
0.4
A
B
C
N18
TAAD
TBAD
CONTROL
0.5
1.5
2.5
A
B
C

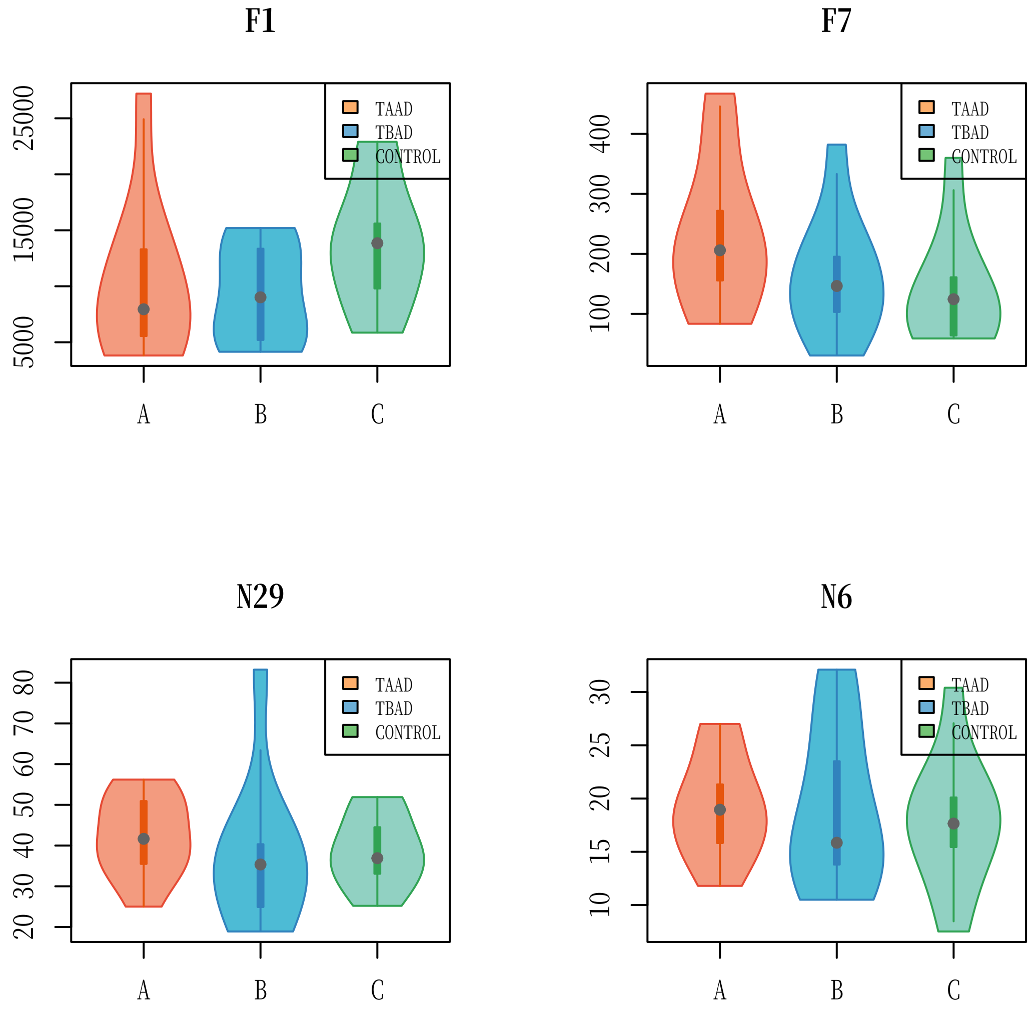
F1
25000
15000
5000
TAAD
TBAD
CONTROL
A
B
C
F7
400
300
200
100
TAAD
TBAD
CONTROL
A
B
C
N29
80
70
60
50
40
30
20
TAAD
TBAD
CONTROL
A
B
C
N6
30
25
20
15
10
TAAD
TBAD
CONTROL
A
B
C

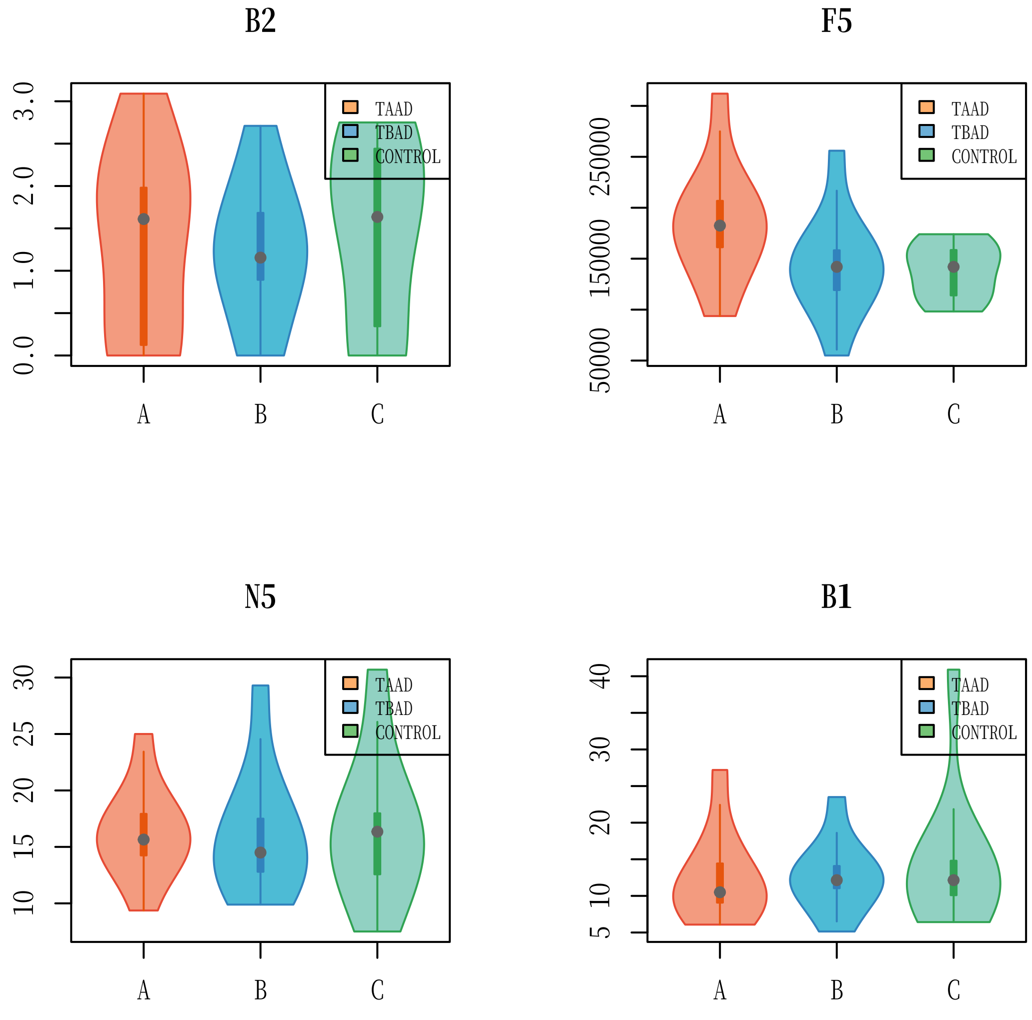
B2
TAAD
TBAD
CONTROL
0.0
1.0
2.0
3.0
A
B
C
F5
TAAD
TBAD
CONTROL
50000
150000
250000
A
B
C
N5
TAAD
TBAD
CONTROL
10
15
20
25
30
A
B
C
B1
TAAD
TBAD
CONTROL
5
10
20
30
40
A
B
C

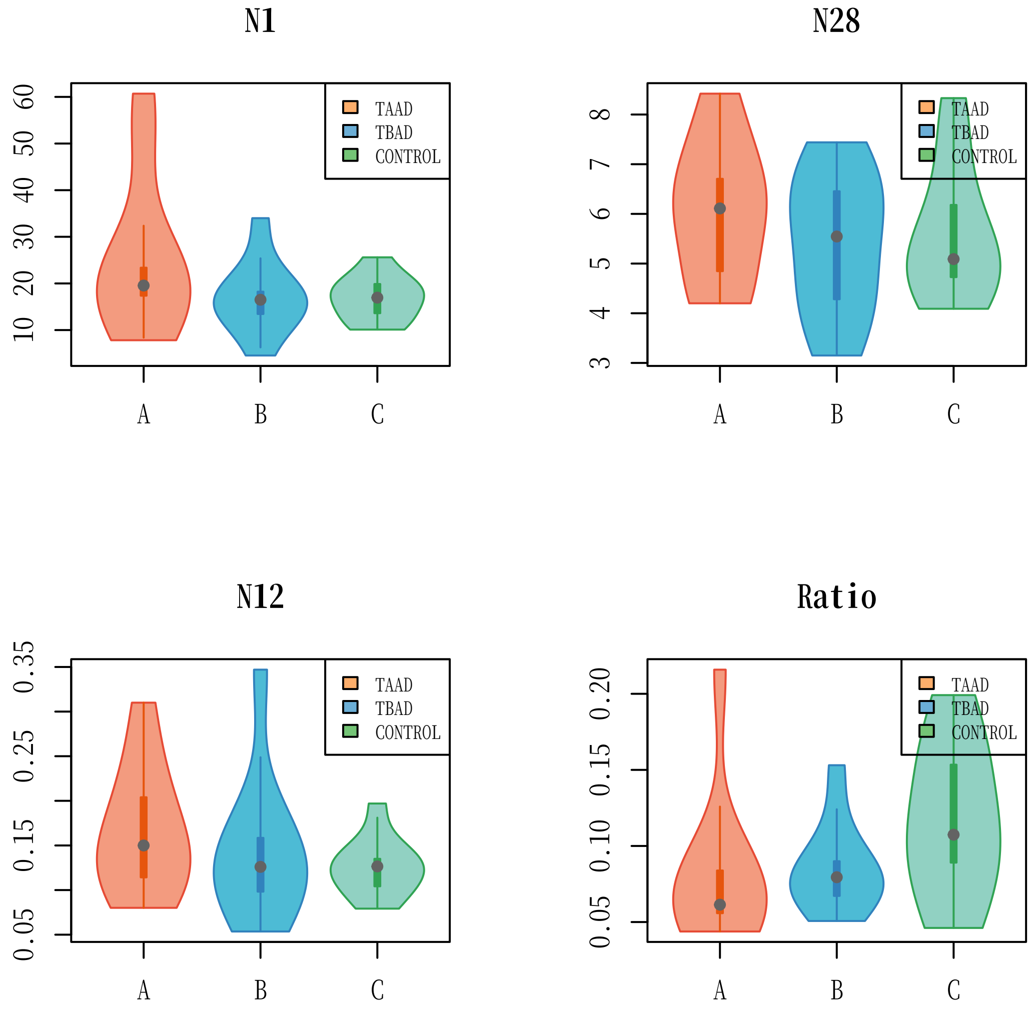

N1
N28
N12
Ratio
TAAD
TBAD
CONTROL
A
B
C
10
20
30
40
50
60
3
4
5
6
7
8
0.05
0.15
0.25
0.35
0.05
0.10
0.15
0.20
