# Supplementary material for: Serum Oxylipin Profiles Identify Potential Biomarkers in Patients with Acute Aortic Dissection
Source: Metabolites. 2022 Jun 23;12(7):587. doi: 10.3390/metabo12070587 (PMC9324768; doi:10.3390/metabo12070587)
Supplement: Supplementary file 1 [file metabolites-12-00587-s001.zip › Supplementary.pdf]

metabolites

MDPI

Supplementary

Supplementary Figure S1

A Total ion flow chromatograms (TIC) and extraction ion flow chromatograms (XIC) of samples, The abscissa is the retention Time of detection (Time, min), and the ordinate is the Intensity of ion flow (Intensity, cps) of ion detection.

B The quantitative analysis integral correction results of a substance randomly selected in different samples. The abscissa is the retention Time (Time, min) of detection, and the ordinate is the ion current Intensity (Intensity, cps) of ion detection.

C Principal component analysis explicable variation map. The abscissa represents each principal component, the ordinate represents the explicable variation. The left picture depicts the total explainable variance and the right figure depicts the explainable variation of each primary component.

Supplementary Figure S2

Heatmap depicts diverse patterns in three categories of substance classification, based on the salient features discovered using PLS-DA.

Supplementary Figure S3

The abscissa represents the covariance of principal component and metabolite, and the ordinate represents the correlation coefficient of principal component and metabolite. Red dot indicates that the VIP value of metabolites greater than or equal to 1, while green dot indicates that the VIP value of metabolites less than 1.

A-C OPLS-DA S-plot for Group A versus Group B (3A), Group A versus Group C (3B), Group B versus Group C (3C).

Supplementary Figure S4

Violin plots of the top 50 divergent metabolites with the highest VIP value.

Supplementary Figure S5

ROC curve of other oxylipins.

Supplementary Figure S6

The area under the curve (AUC) of the five-index model was 0.905.

Supplementary Figure S7

Correlation analysis between different oxylipins. Shape size and color are corresponding correlation coefficient and significative degree.

Supplementary Table S1

Table of abbreviations and full names of oxylipins.
