# Supplementary figures and images for: Serum Oxylipin Profiles Identify Potential Biomarkers in Patients with Acute Aortic Dissection
Source: Metabolites. 2022 Jun 23;12(7):587. doi: 10.3390/metabo12070587 (PMC9324768; doi:10.3390/metabo12070587)

N31

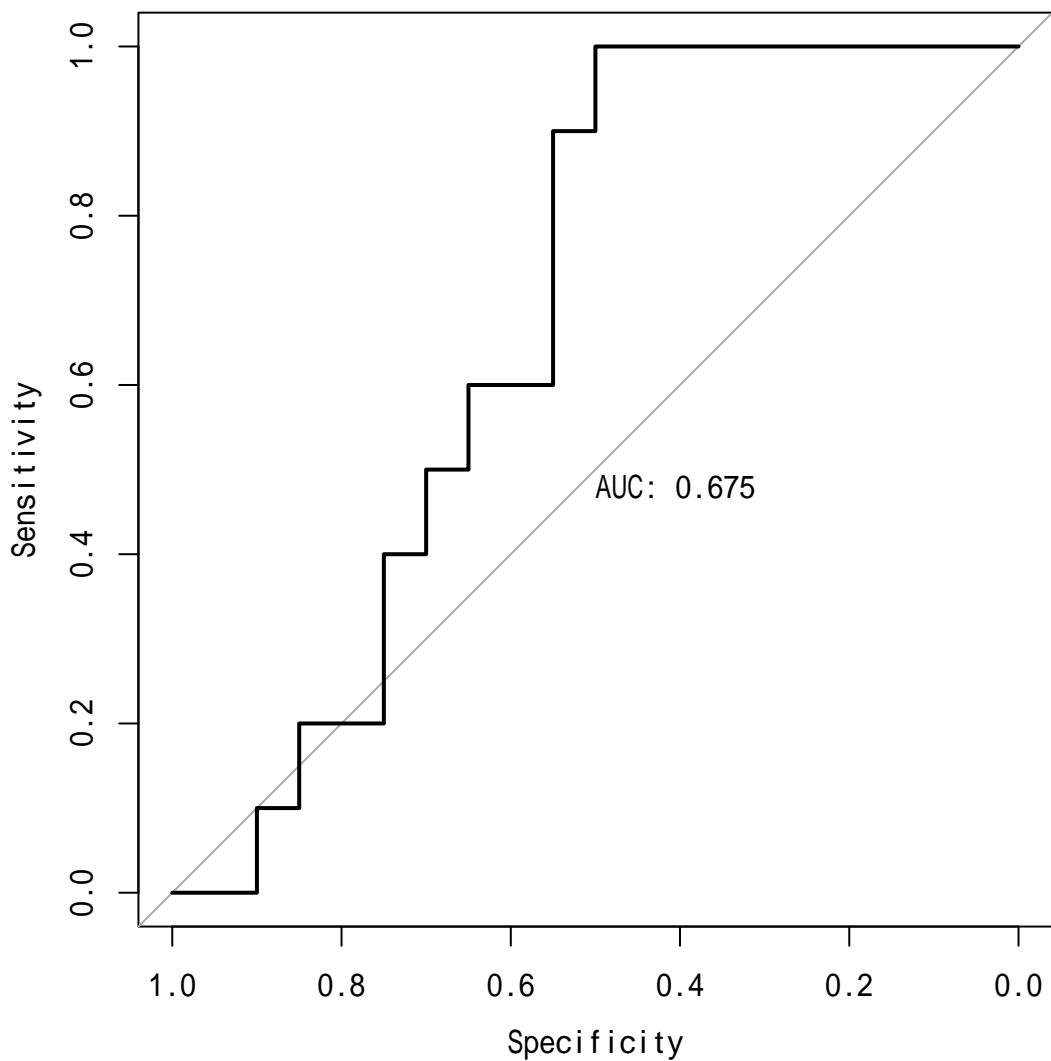

N32

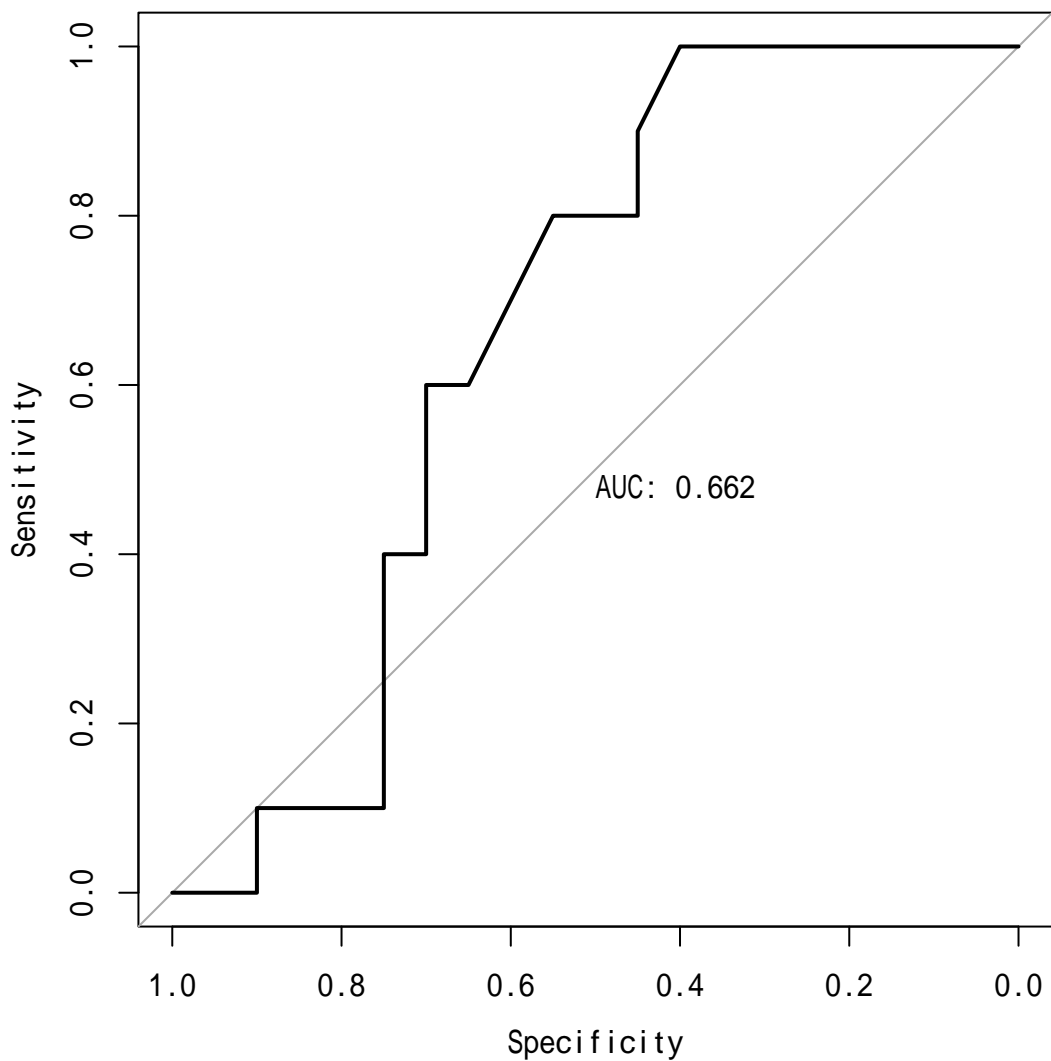

N14

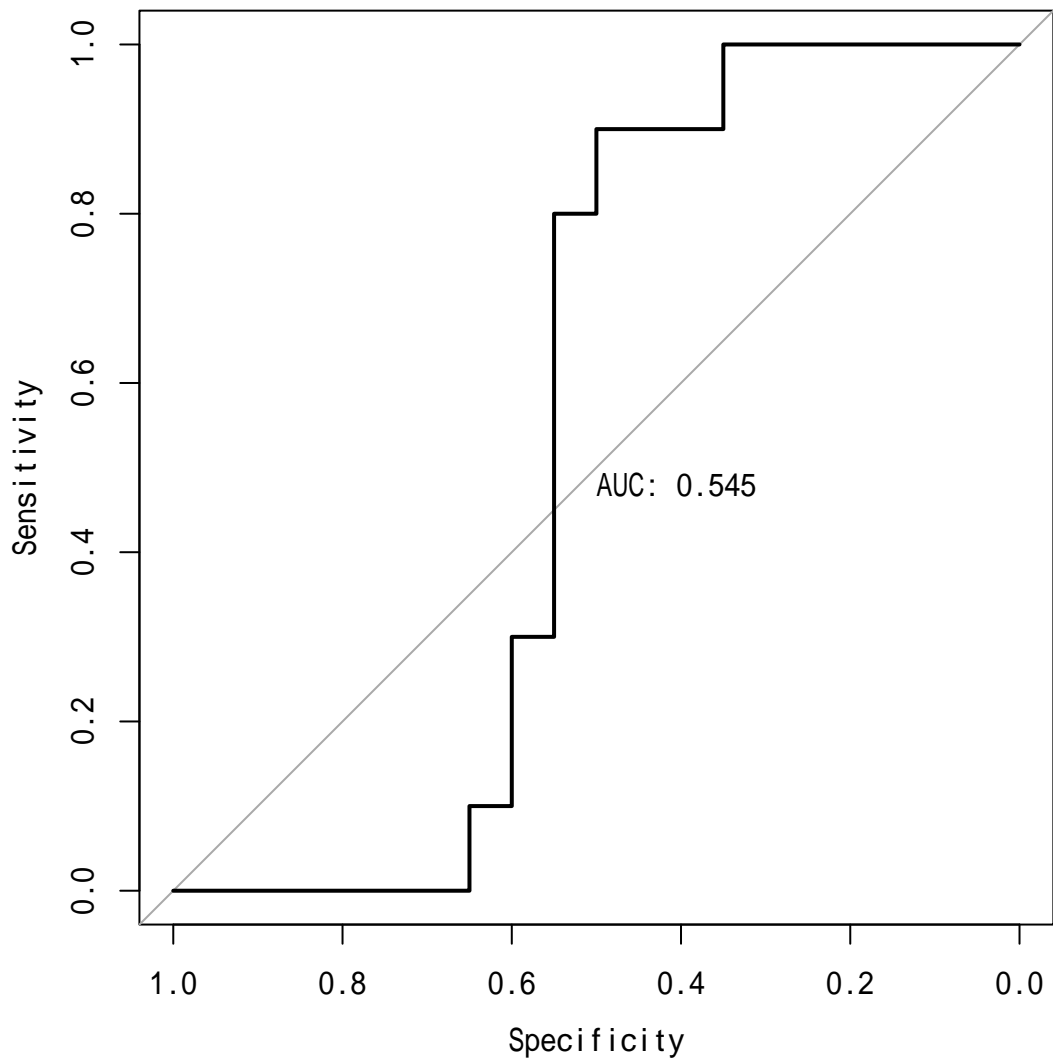

E10

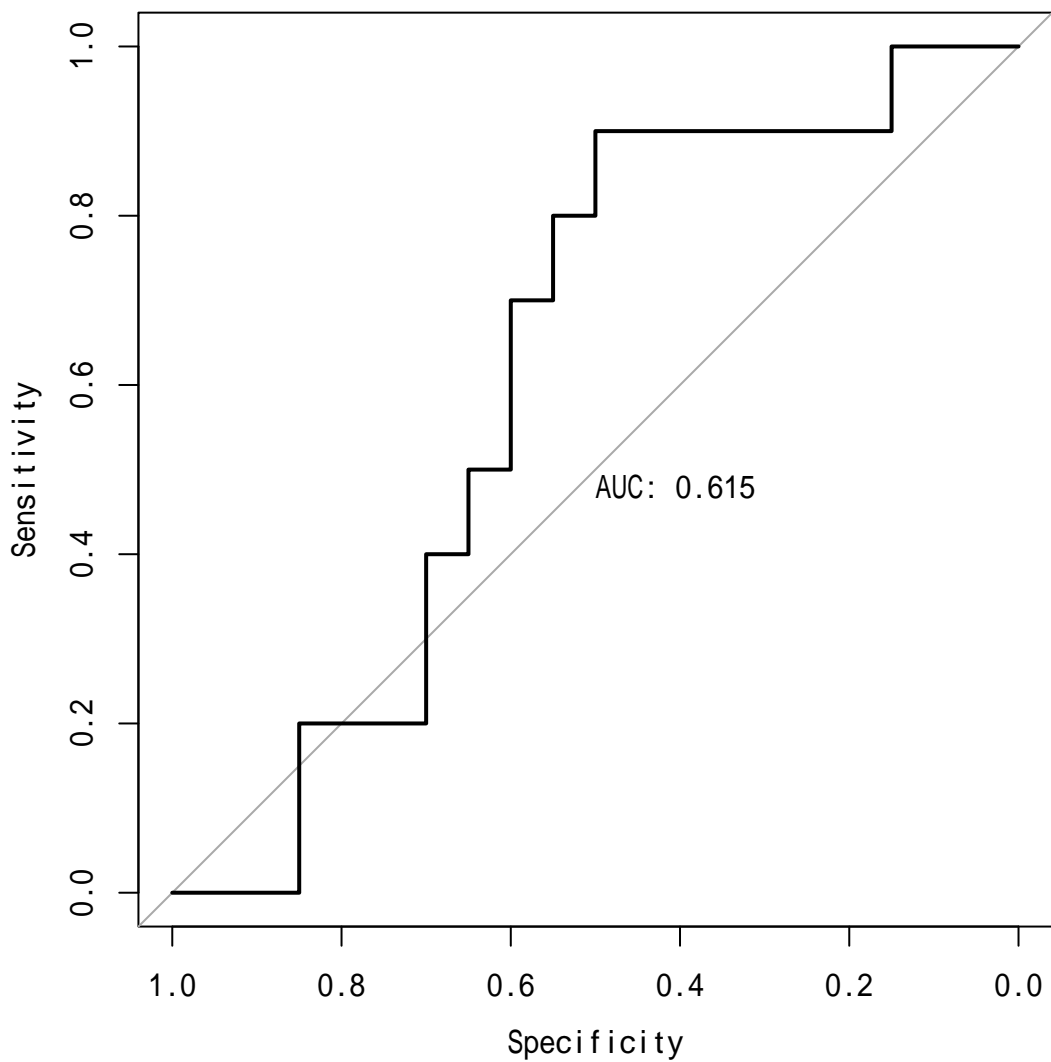

E4

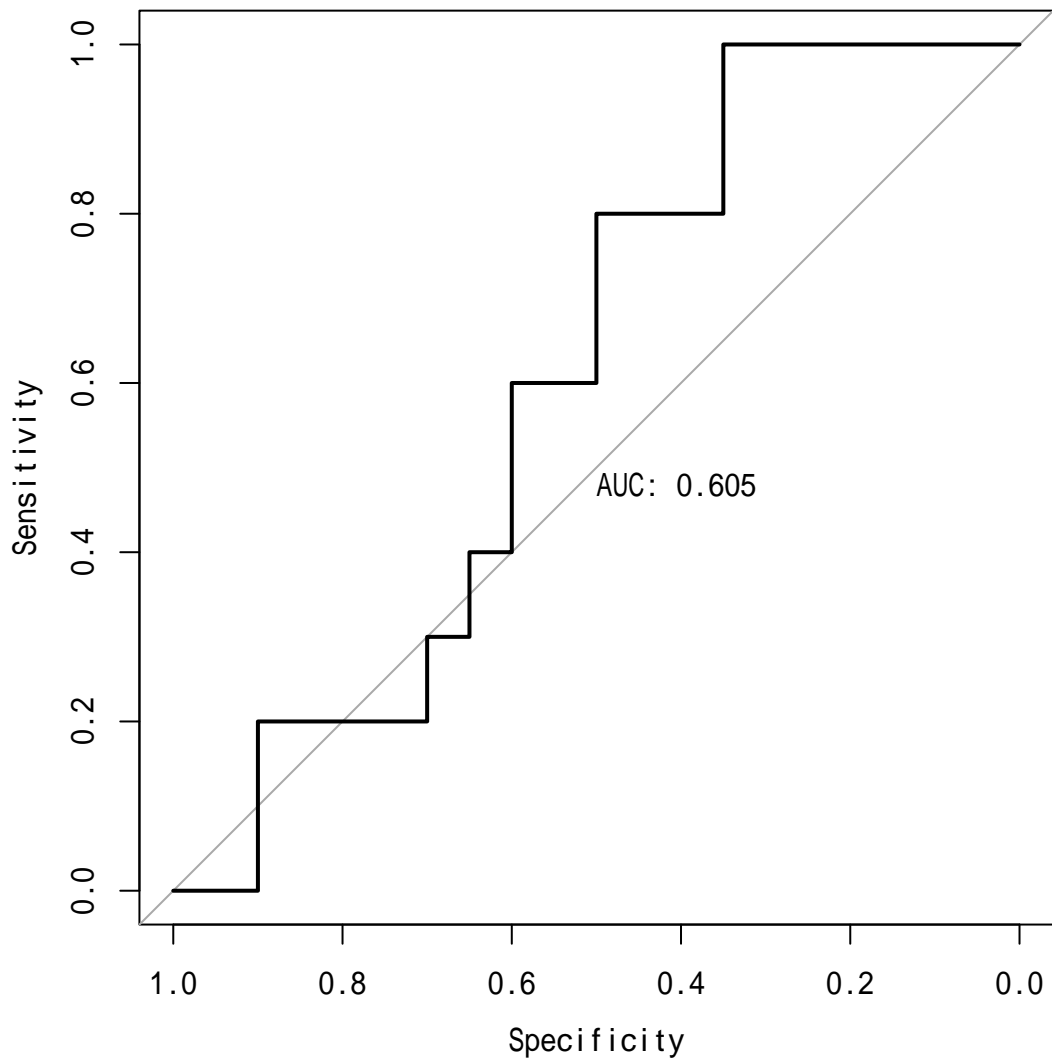

E2

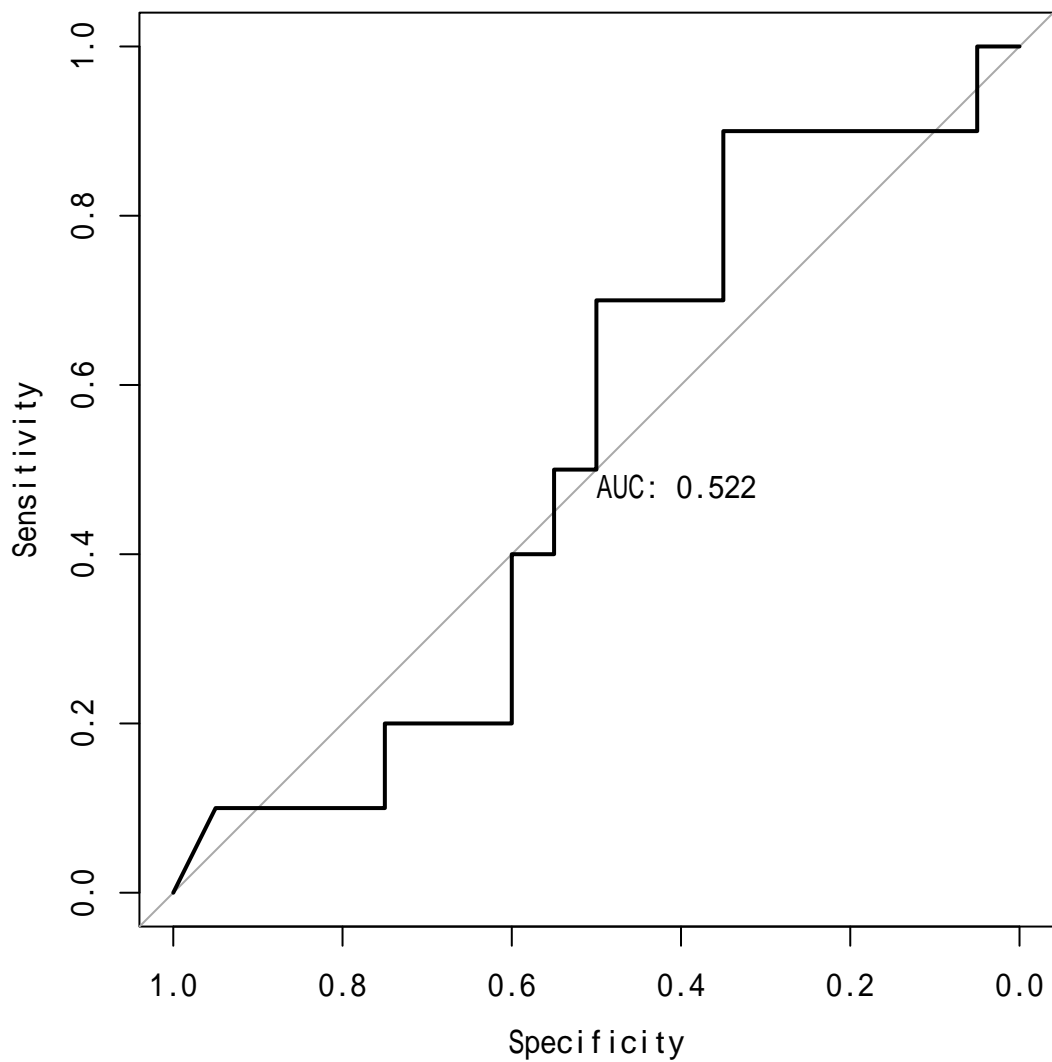

N3

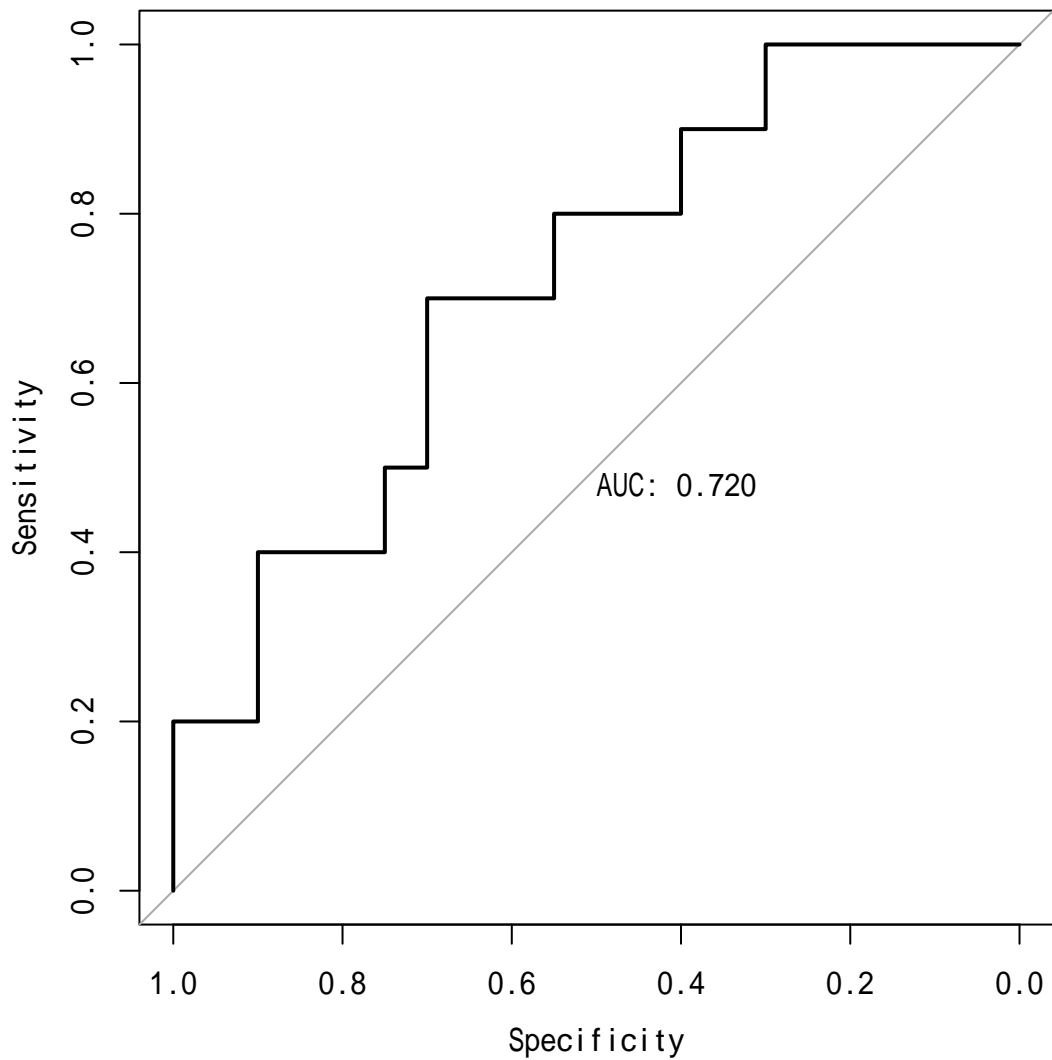

N27

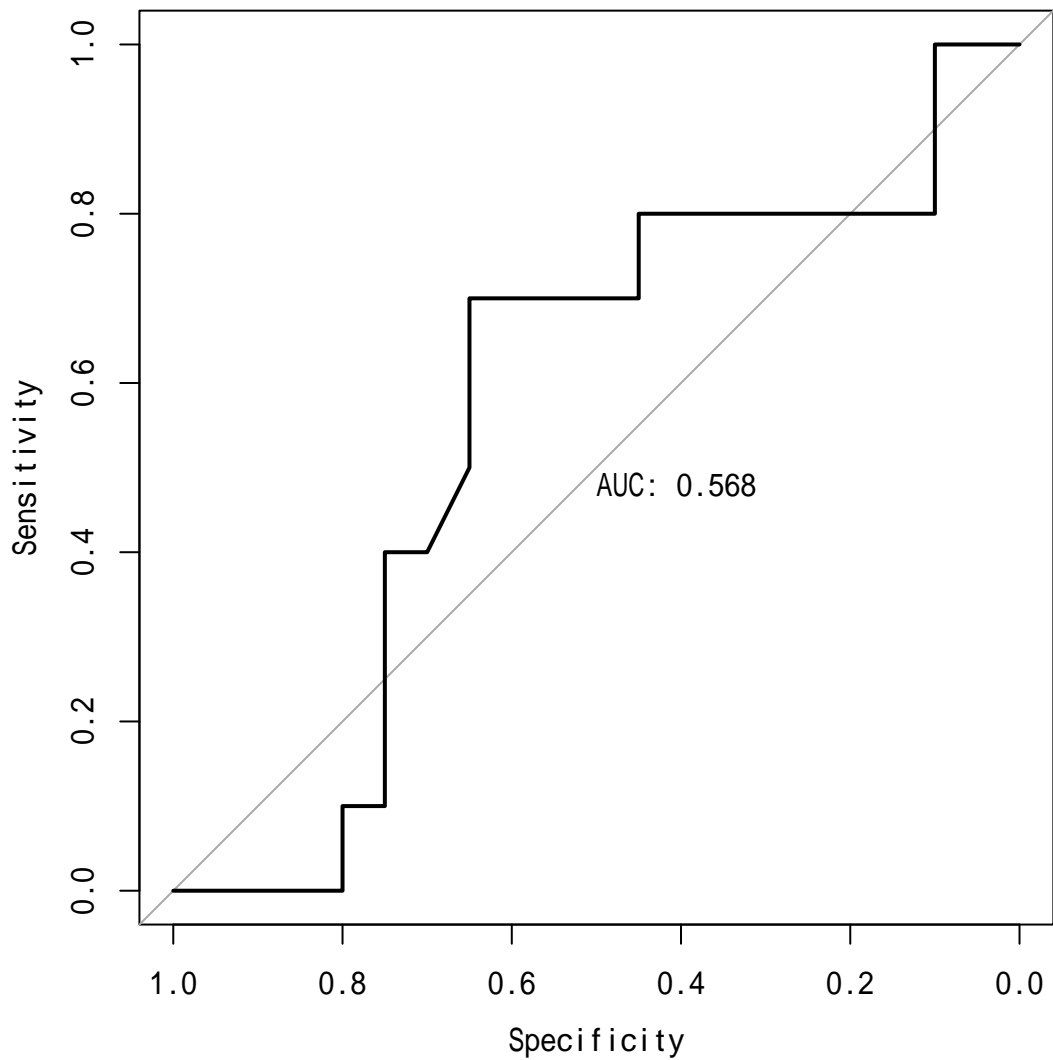

E9

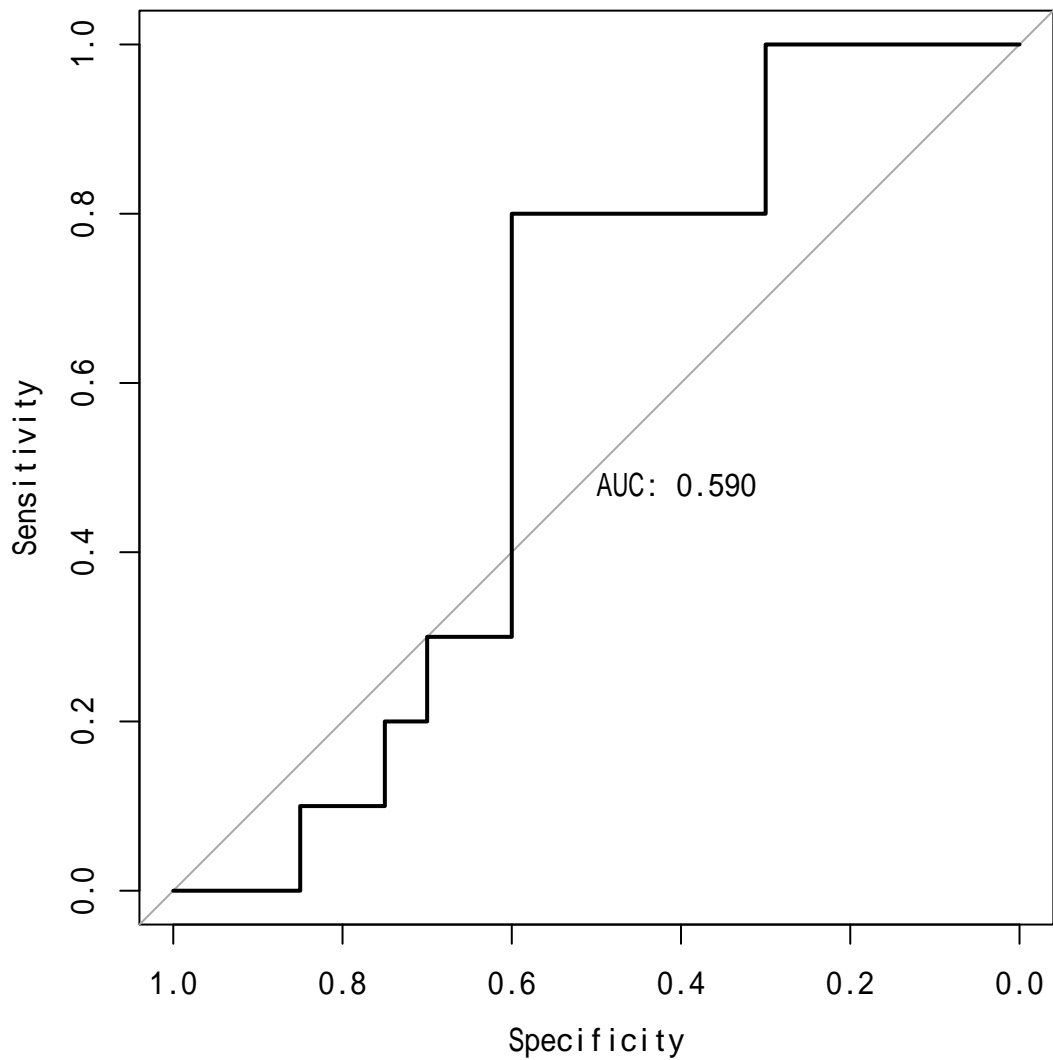

N26

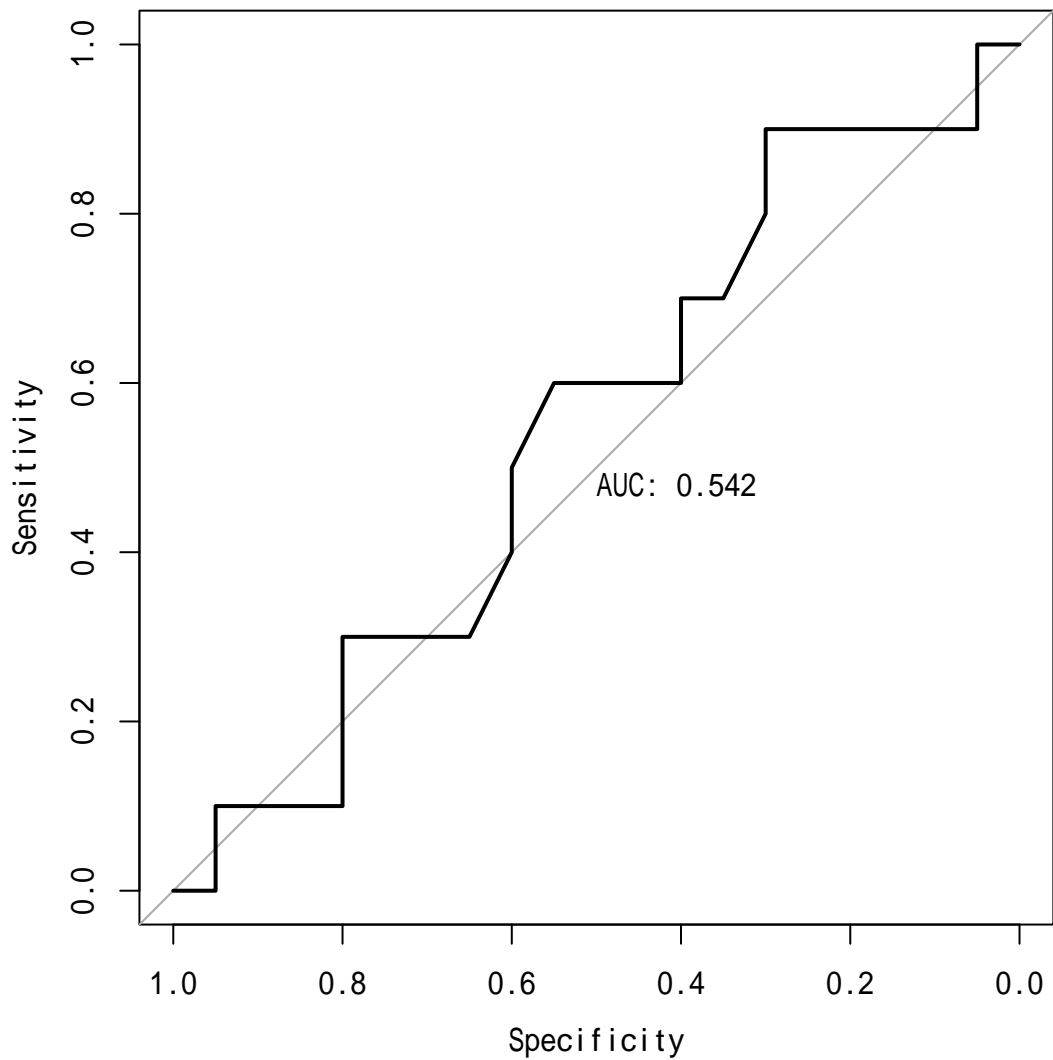

N2

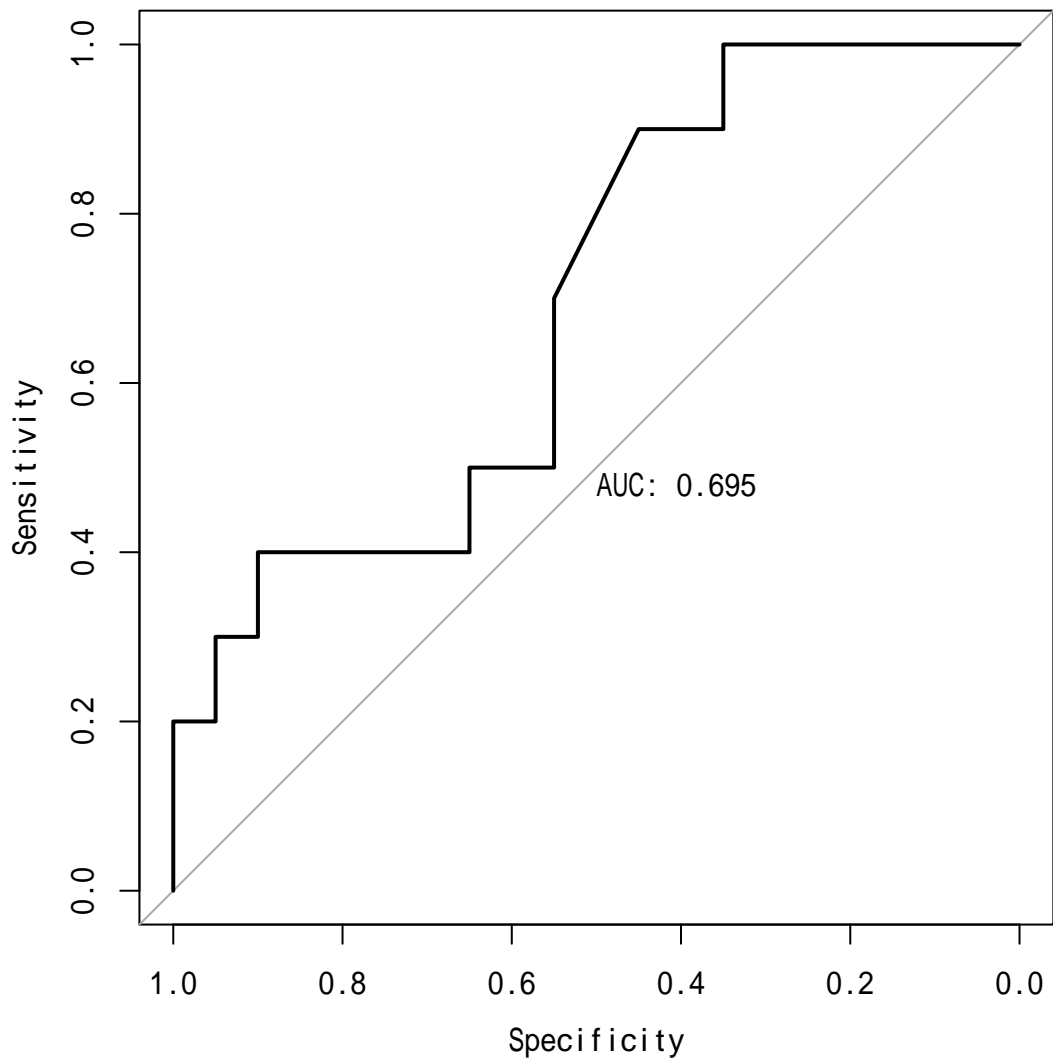

N4

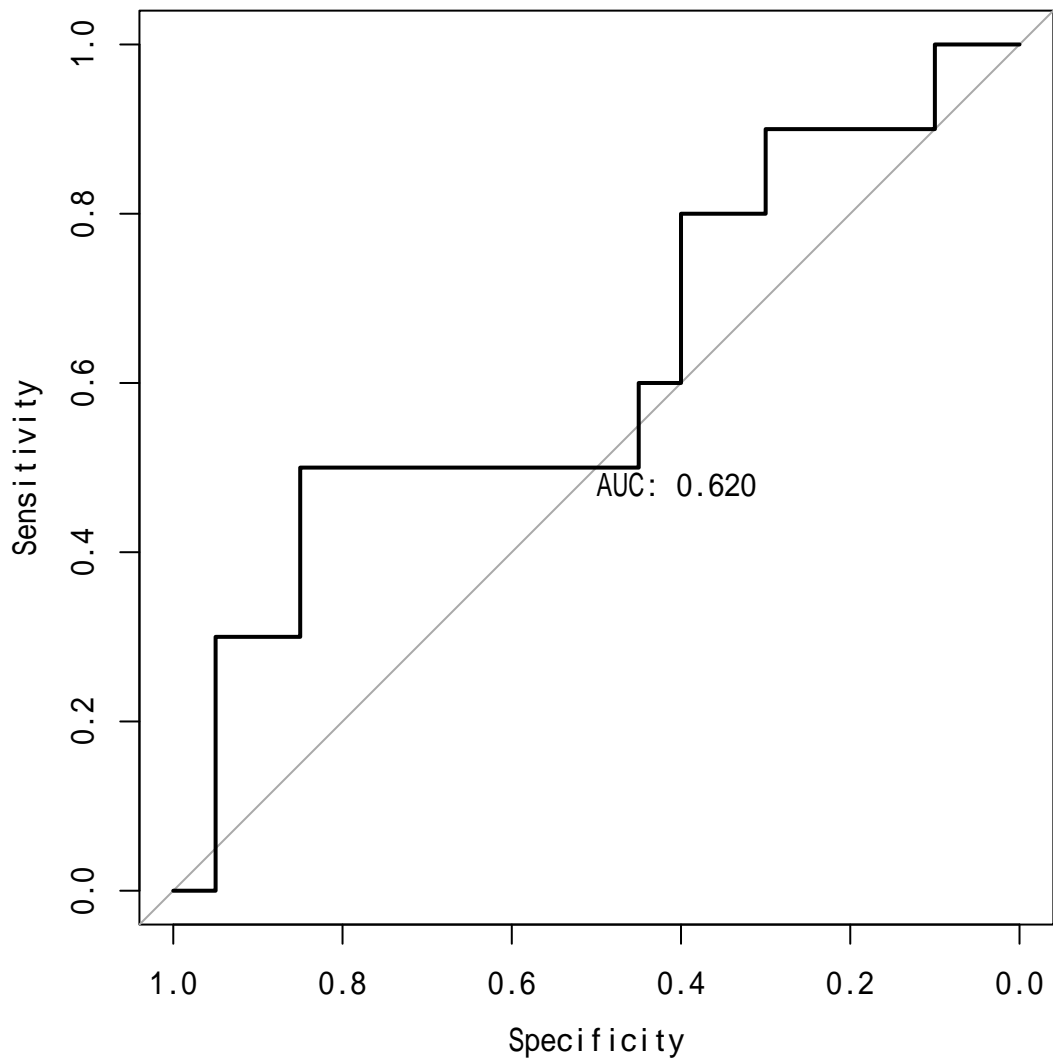

F8

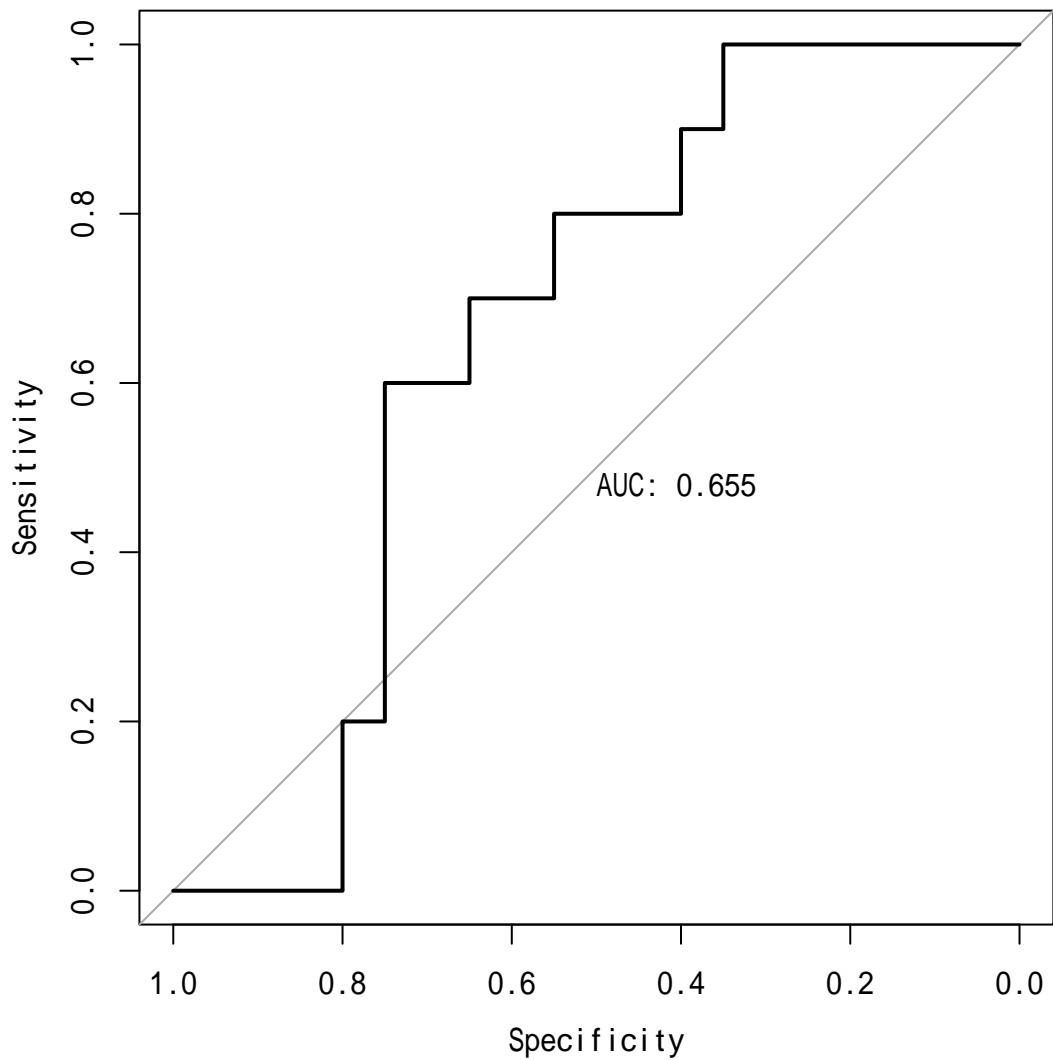

A3

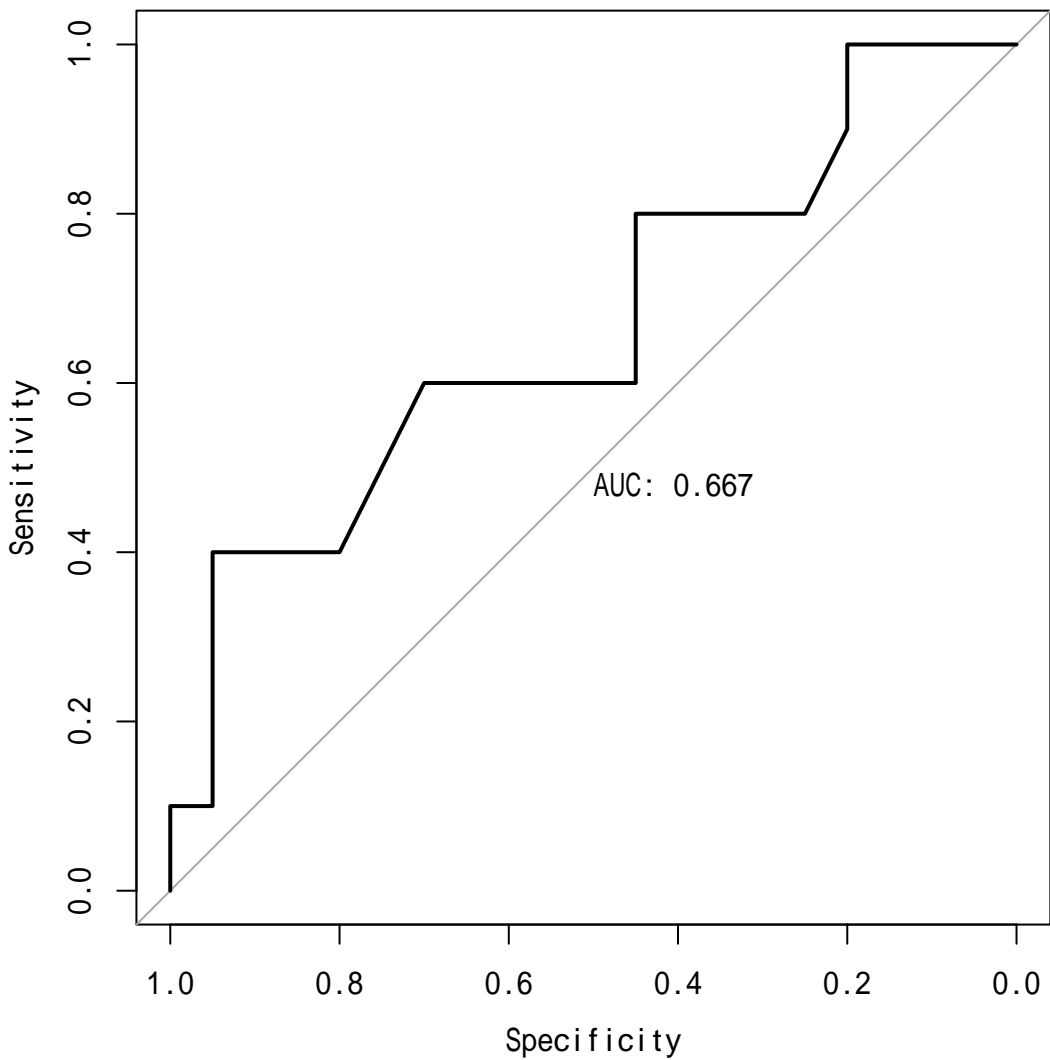

A2

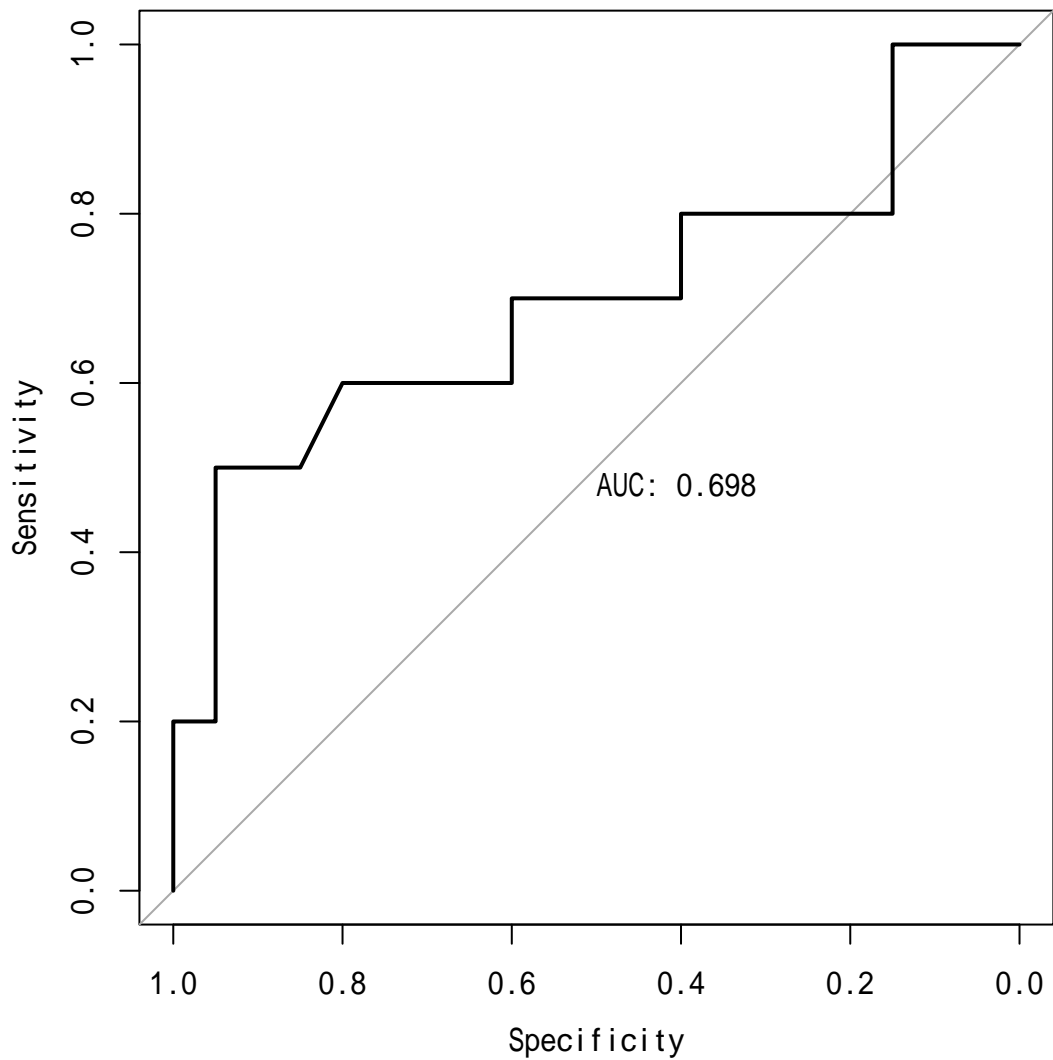

N19

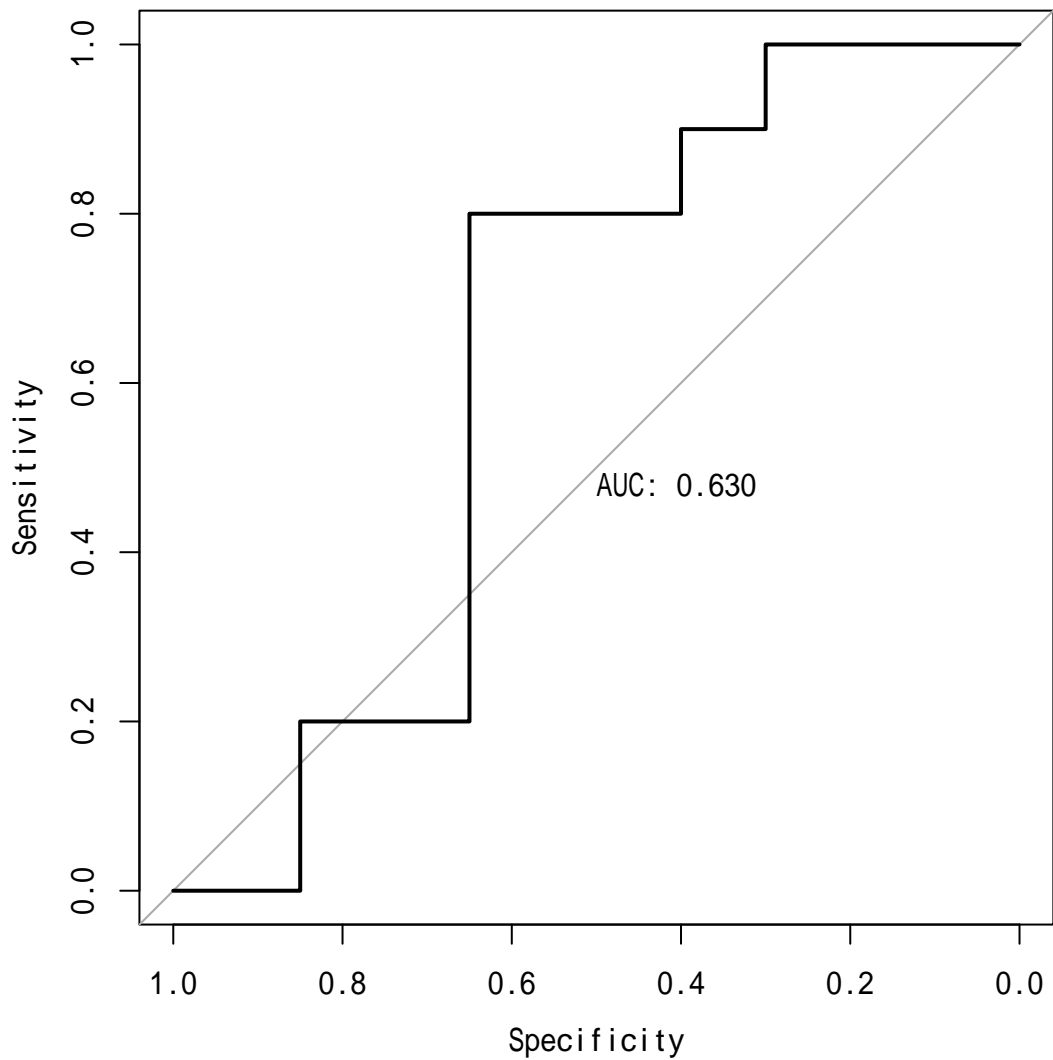

A1

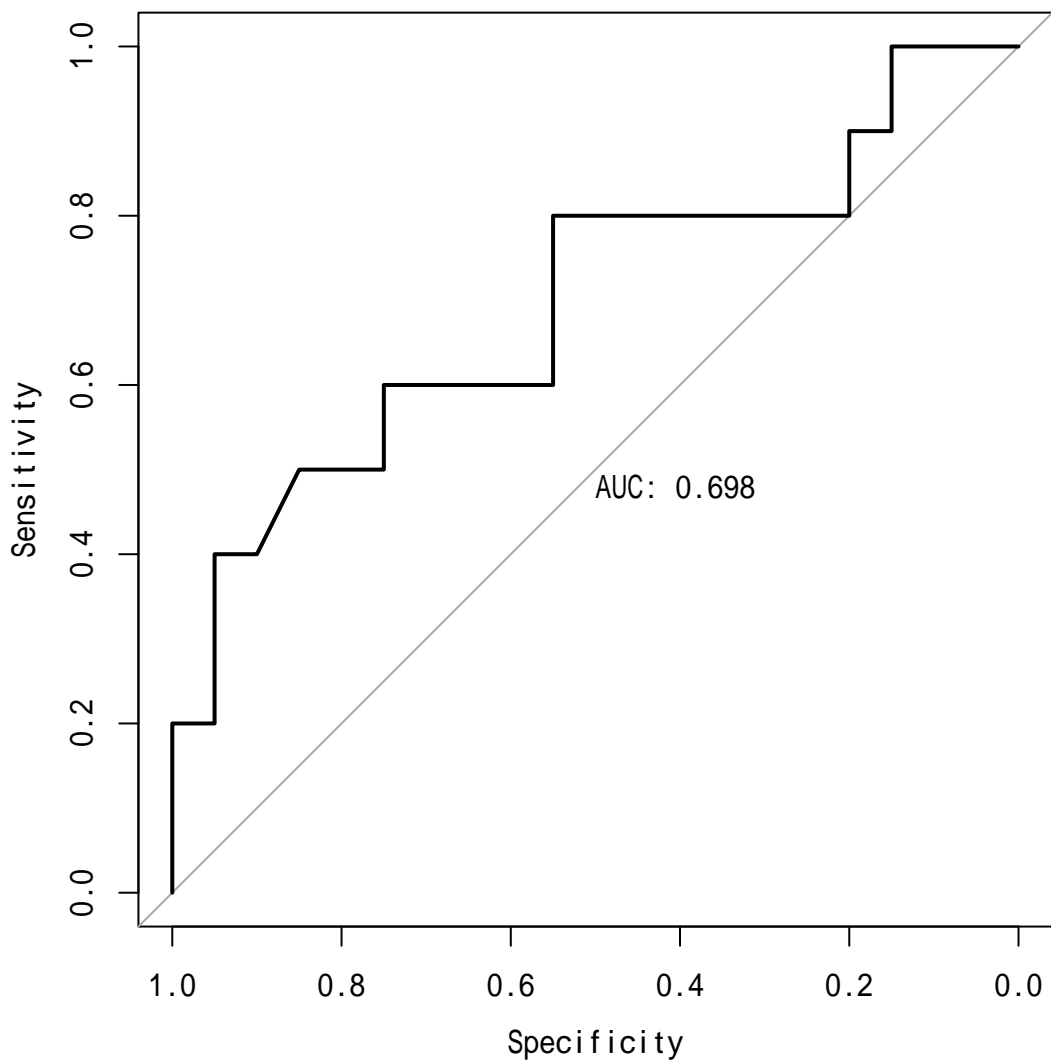

L15

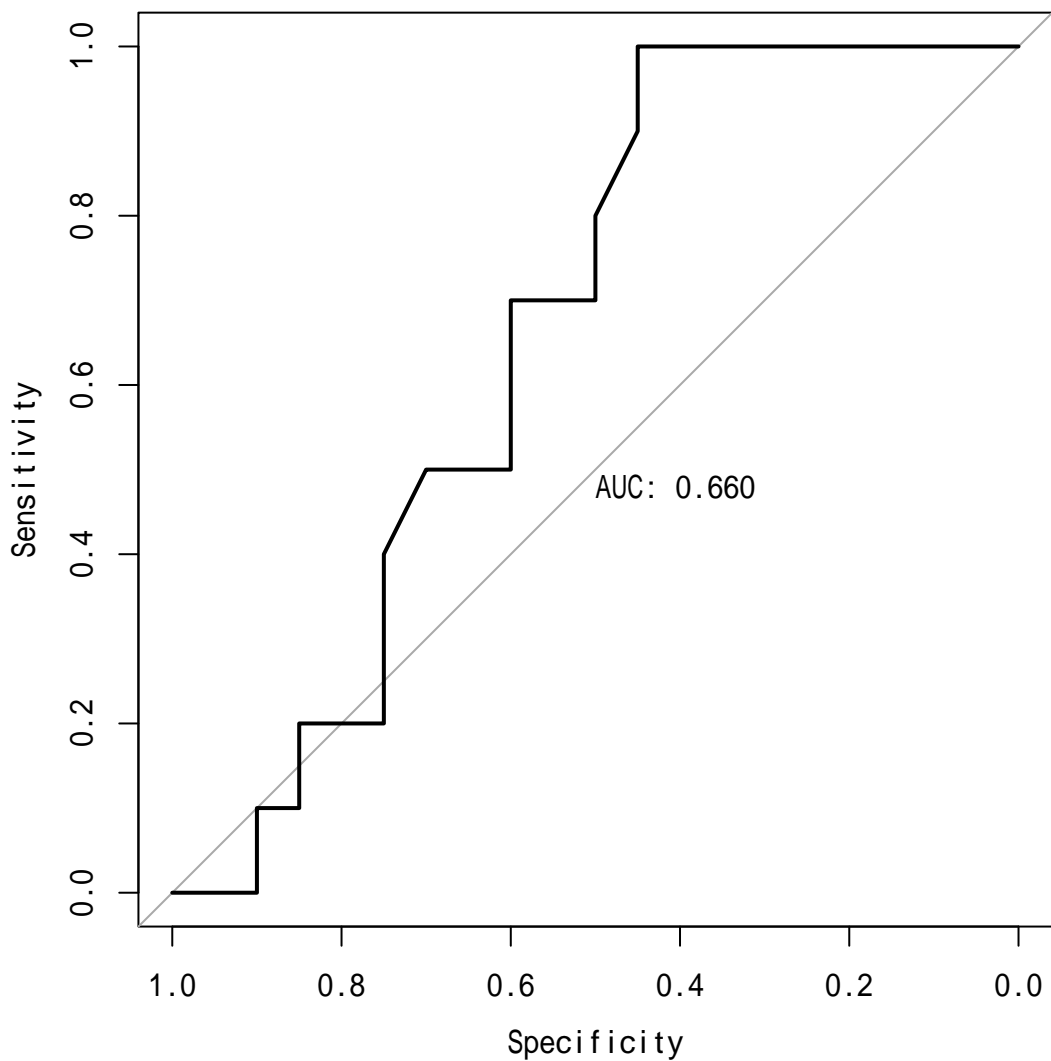

E7

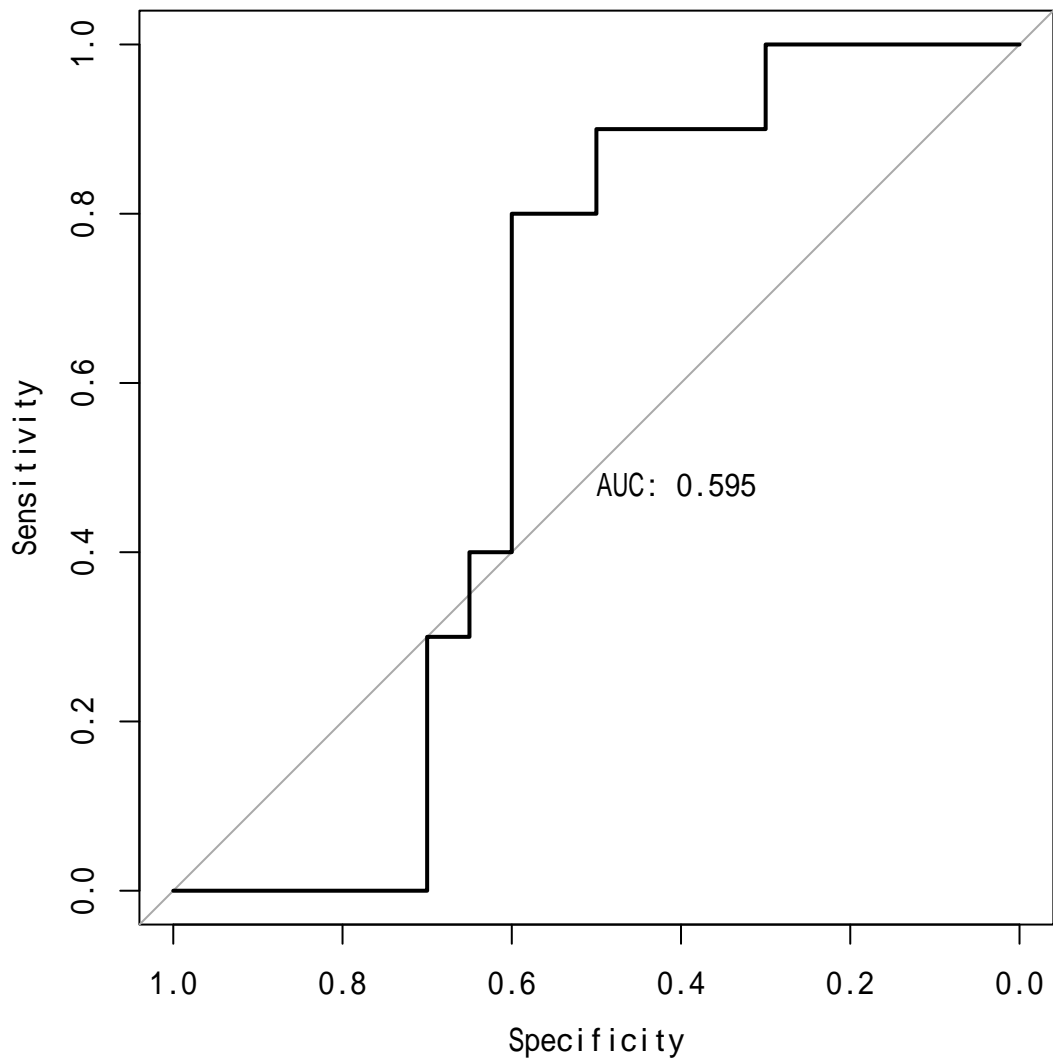

E8

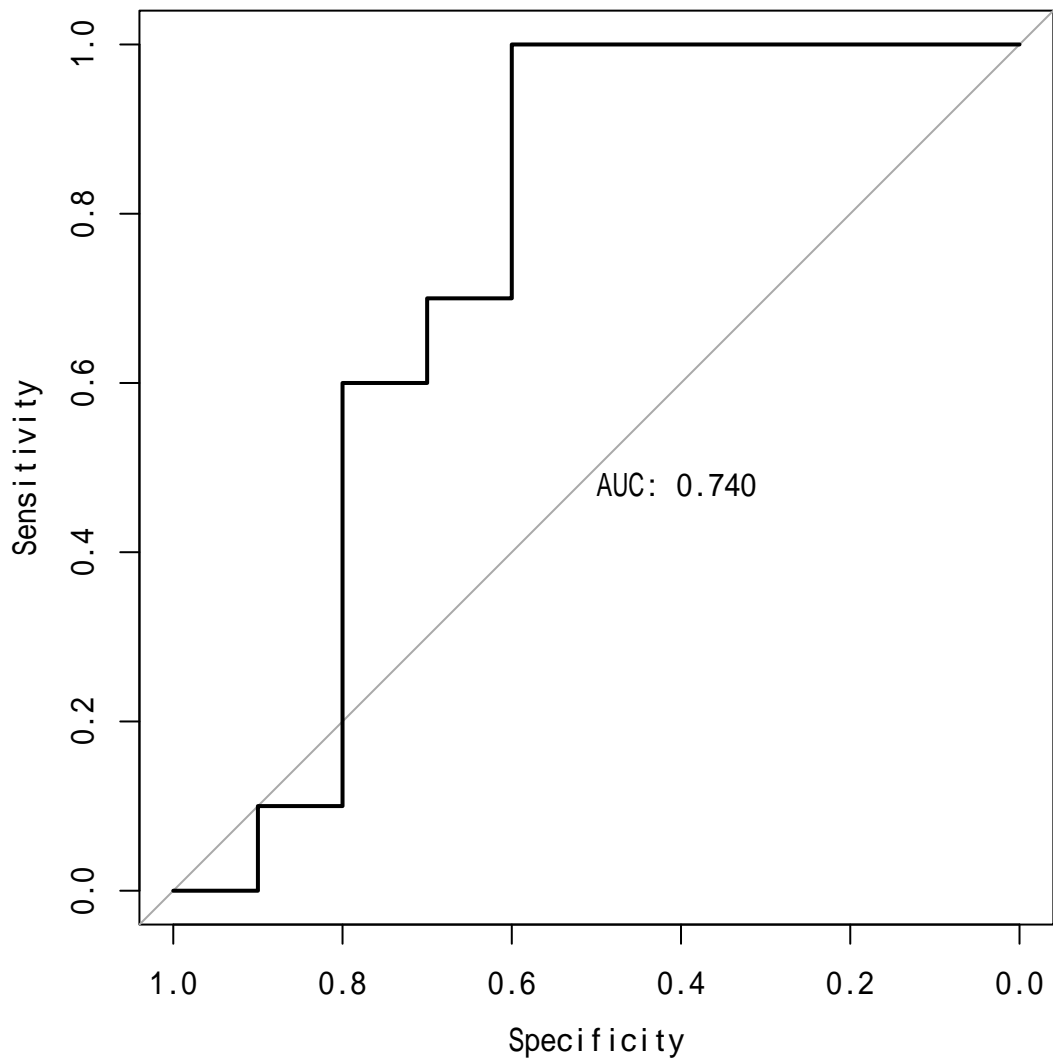

E5

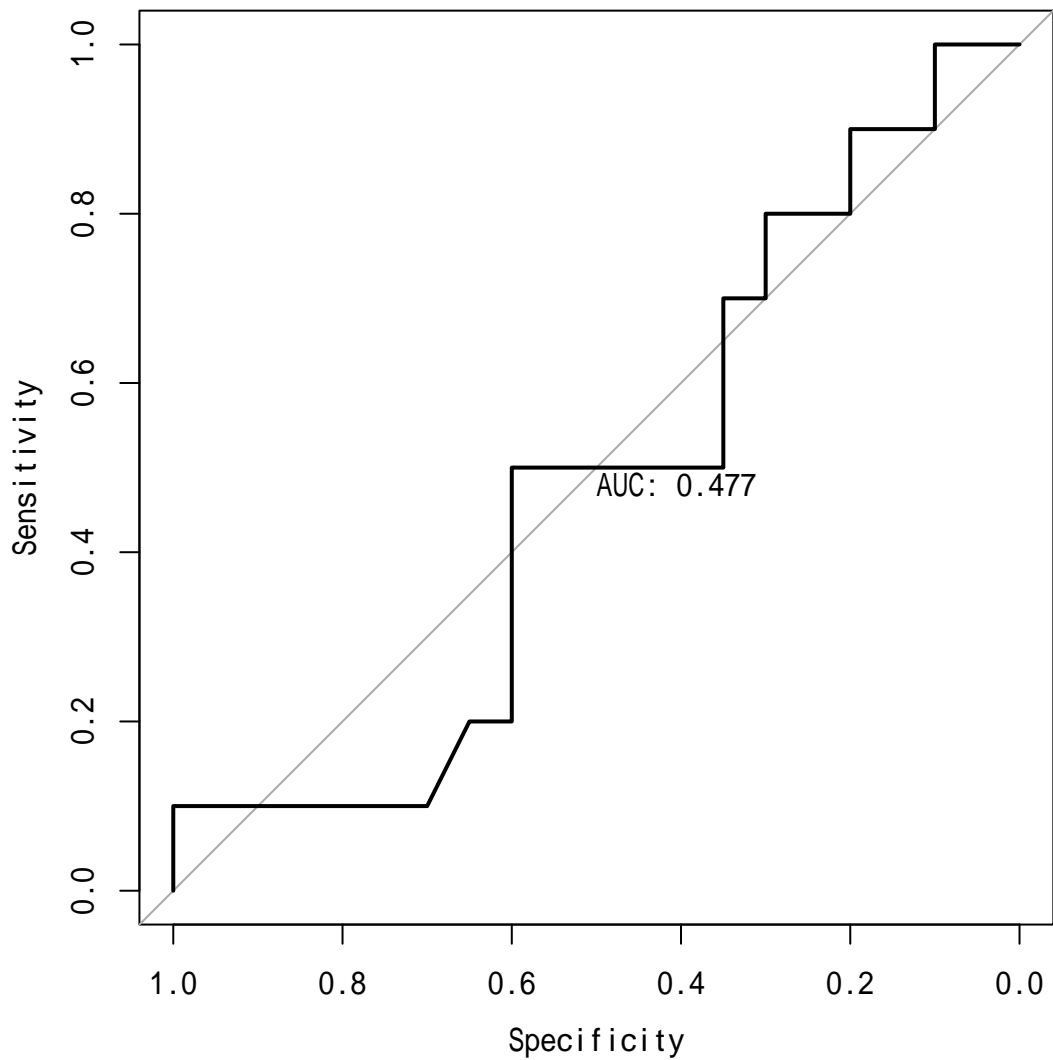

E3

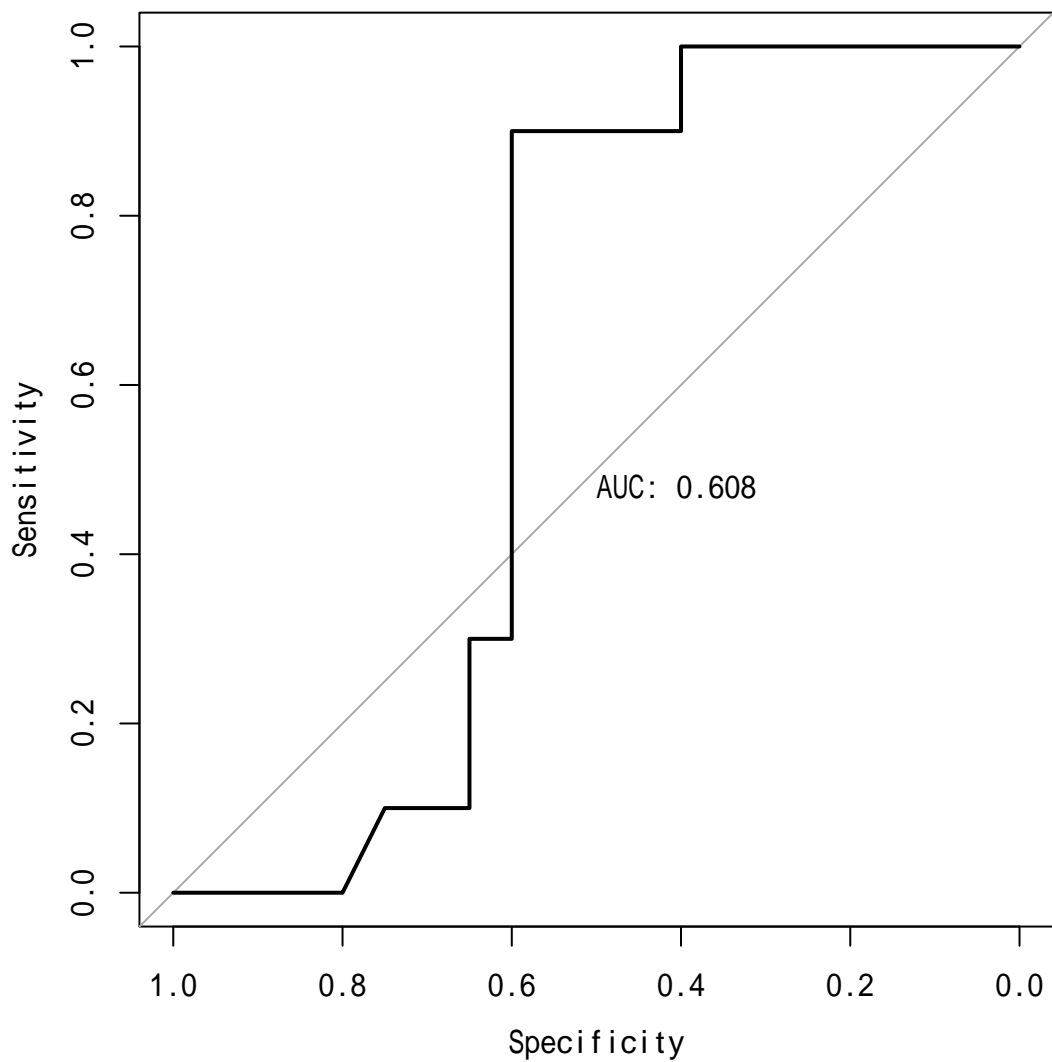

E12

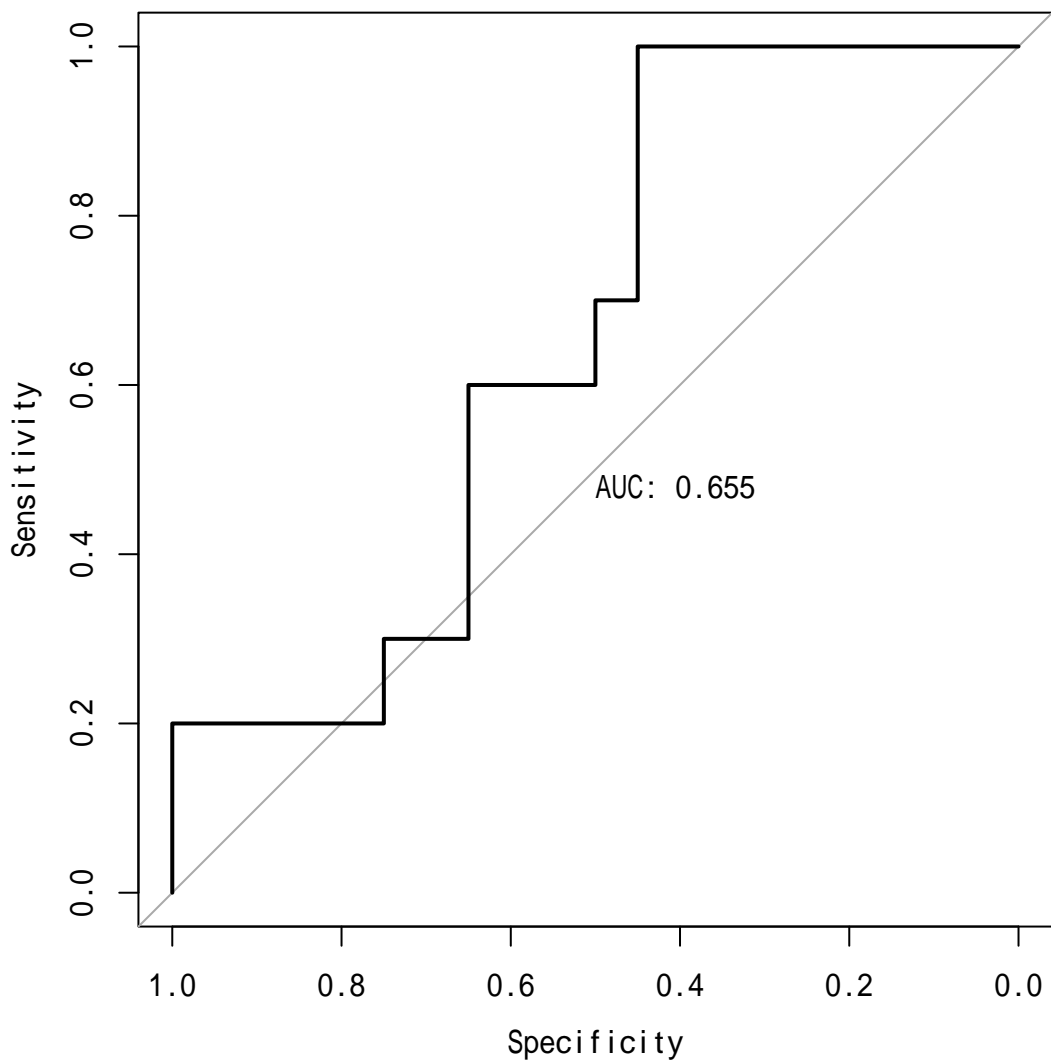

E11

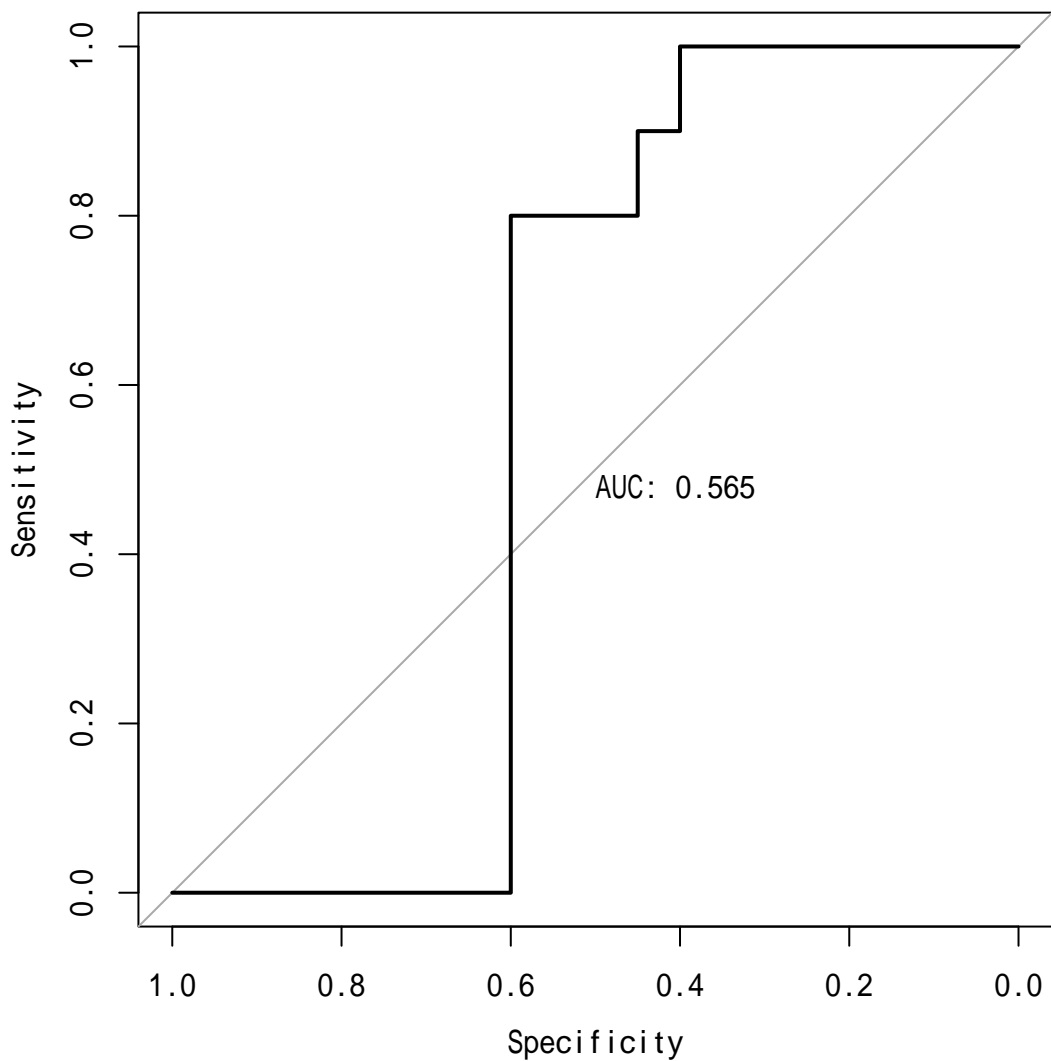

F2

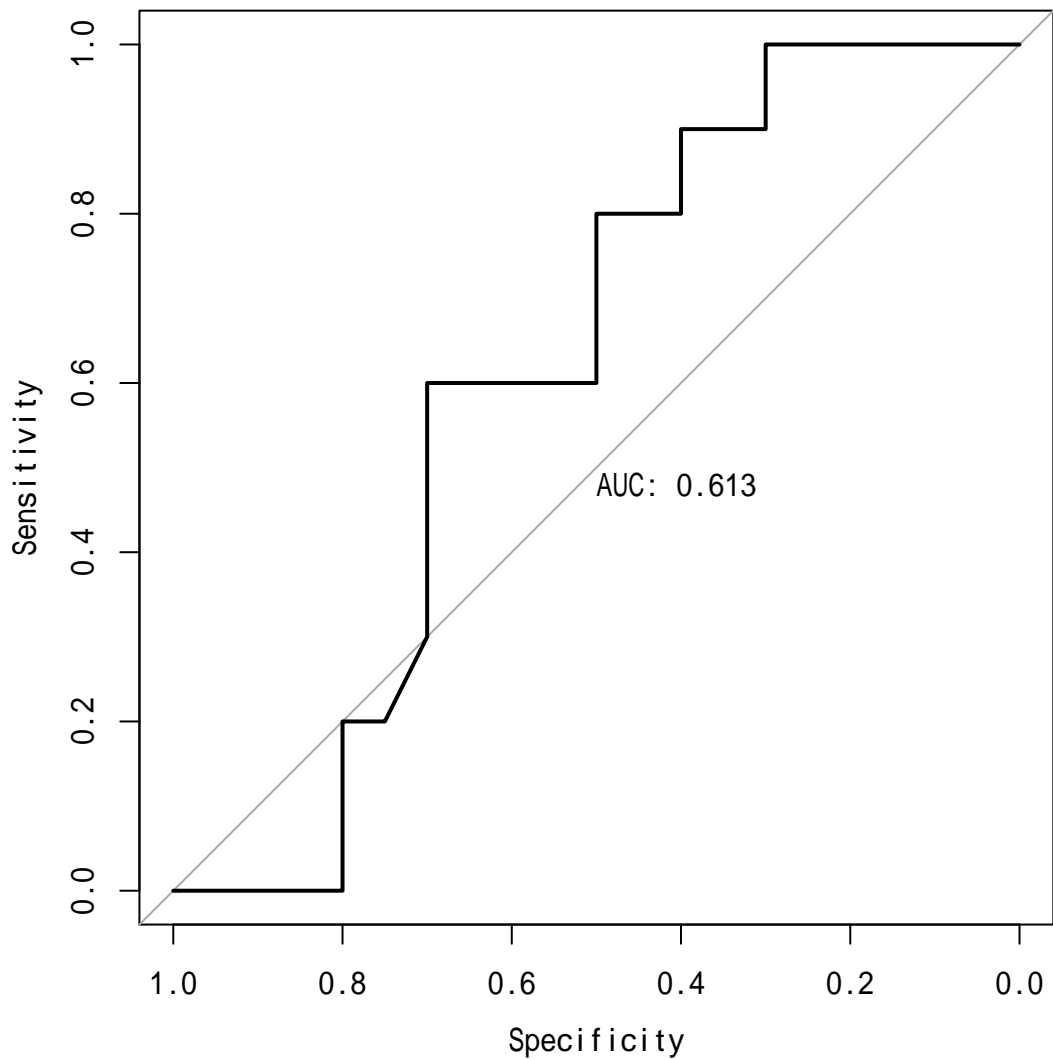

E6

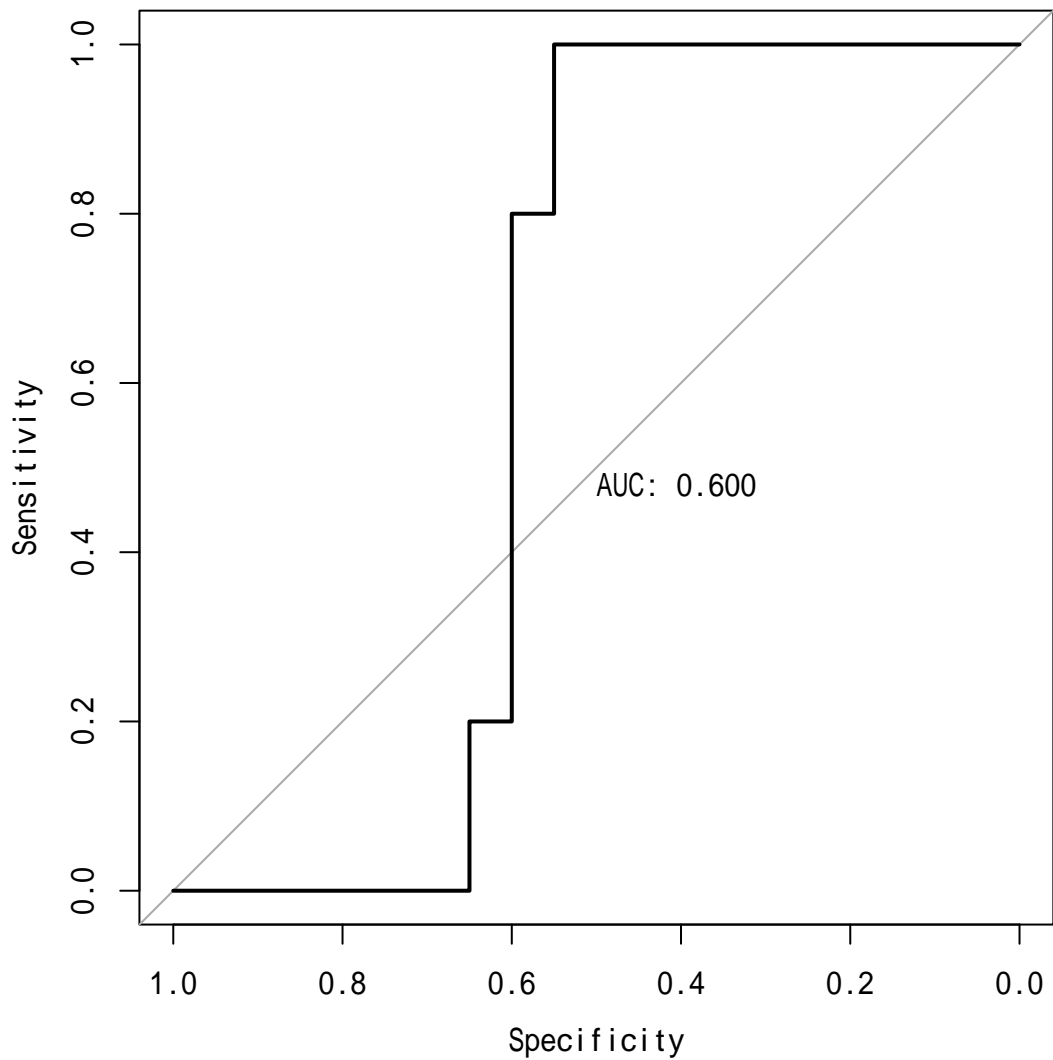

JJ2

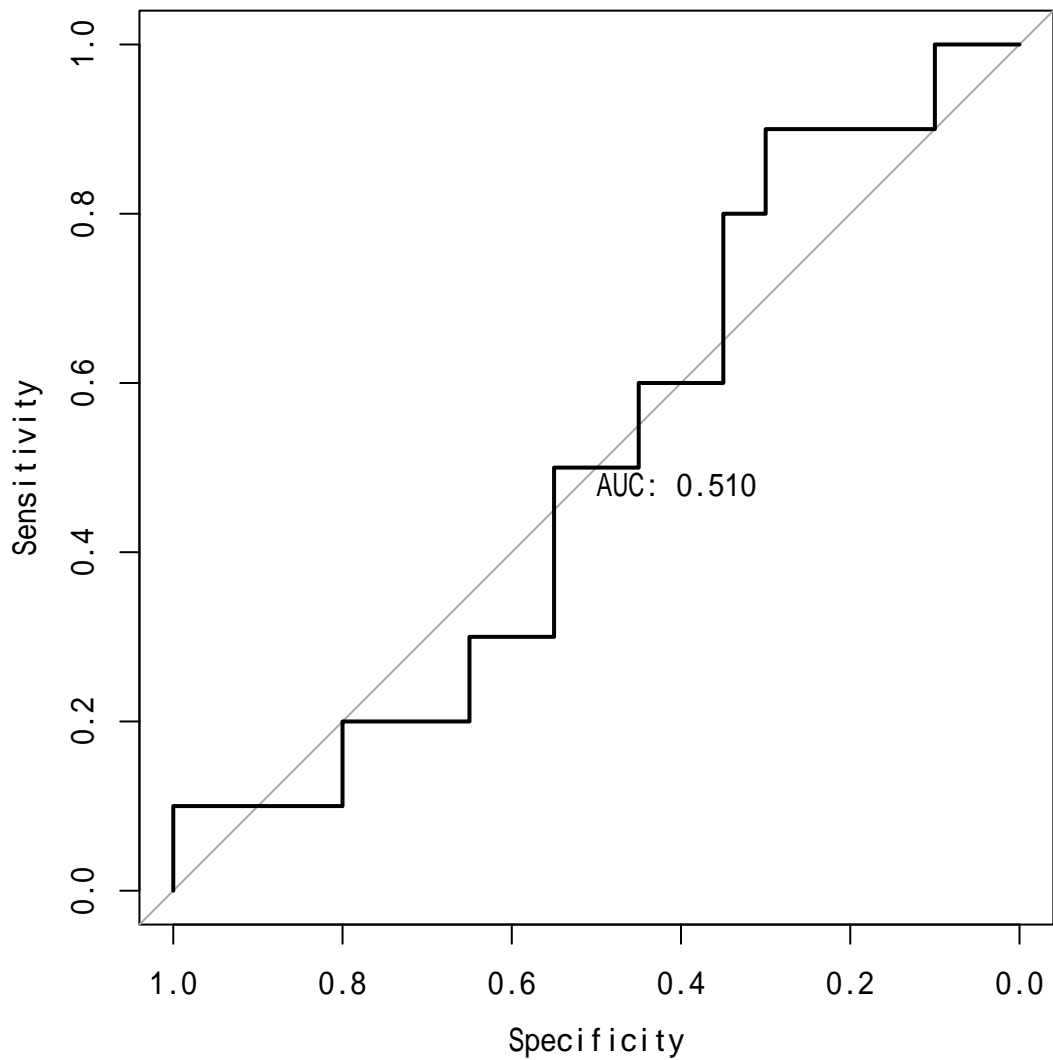

N21

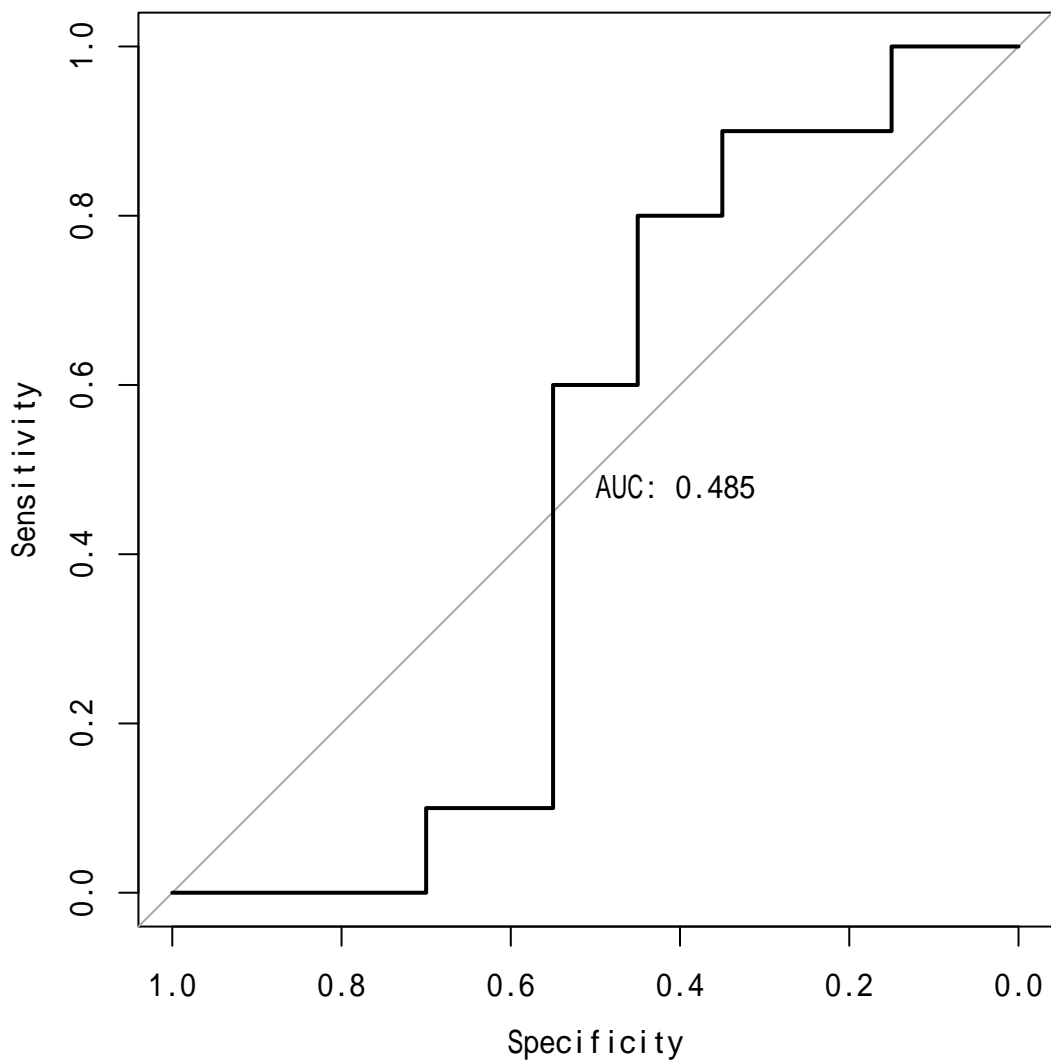

N20

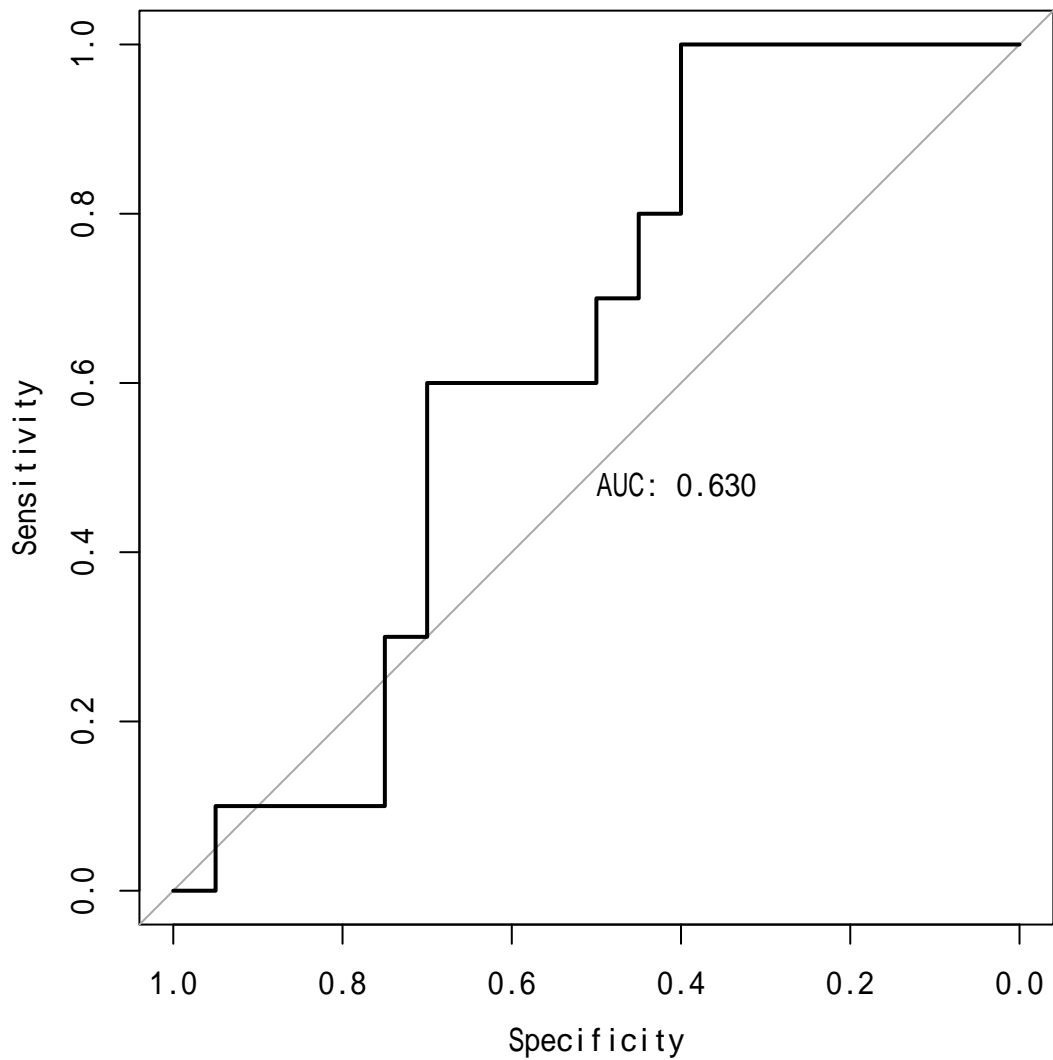

F4

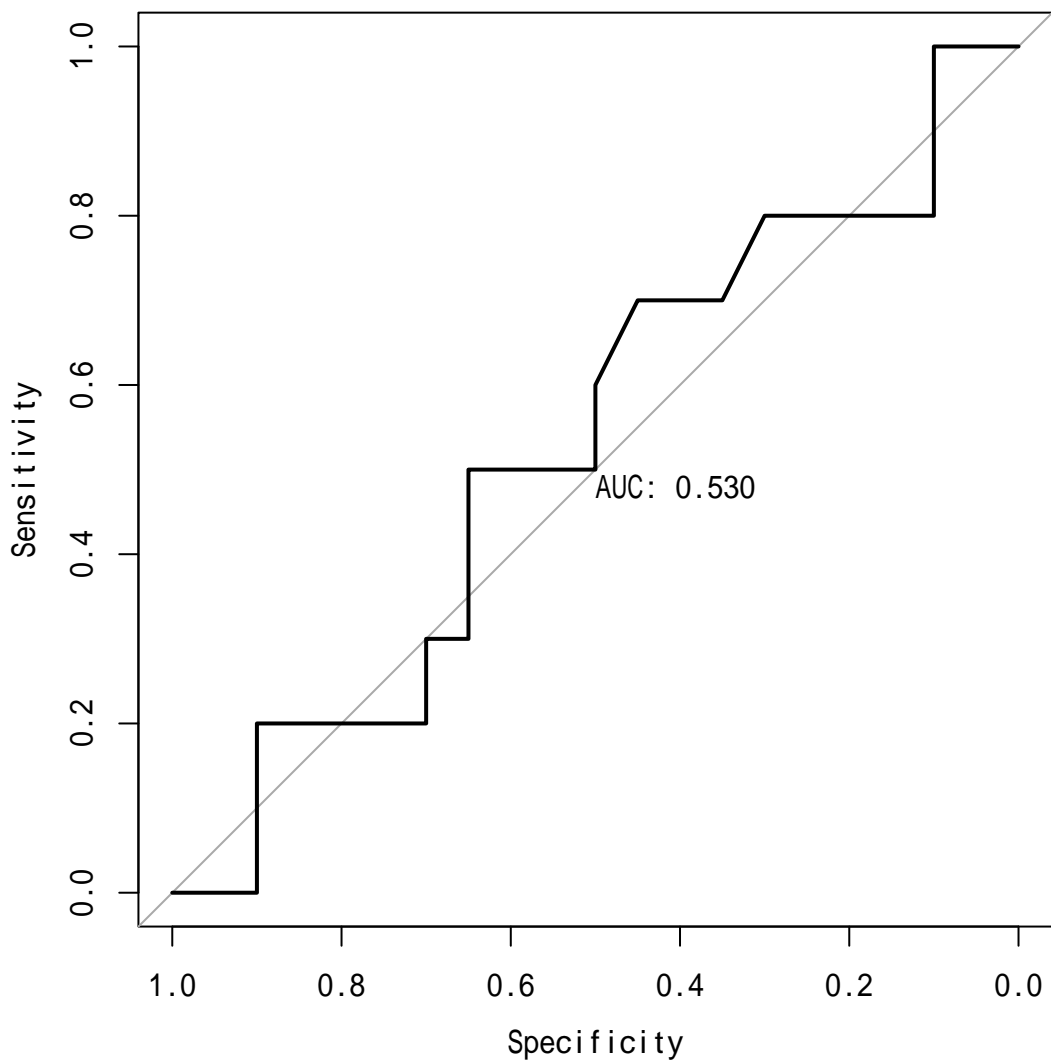

G1

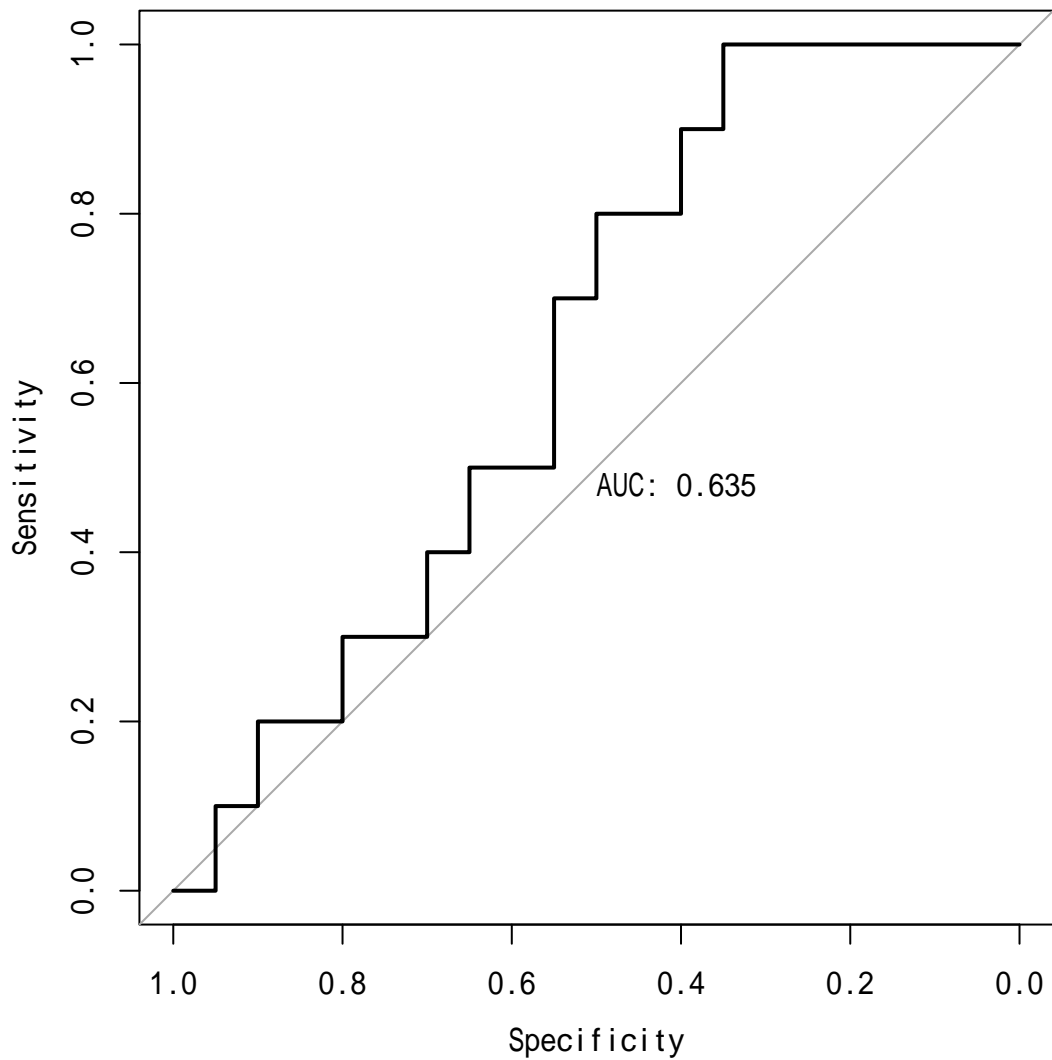

N25

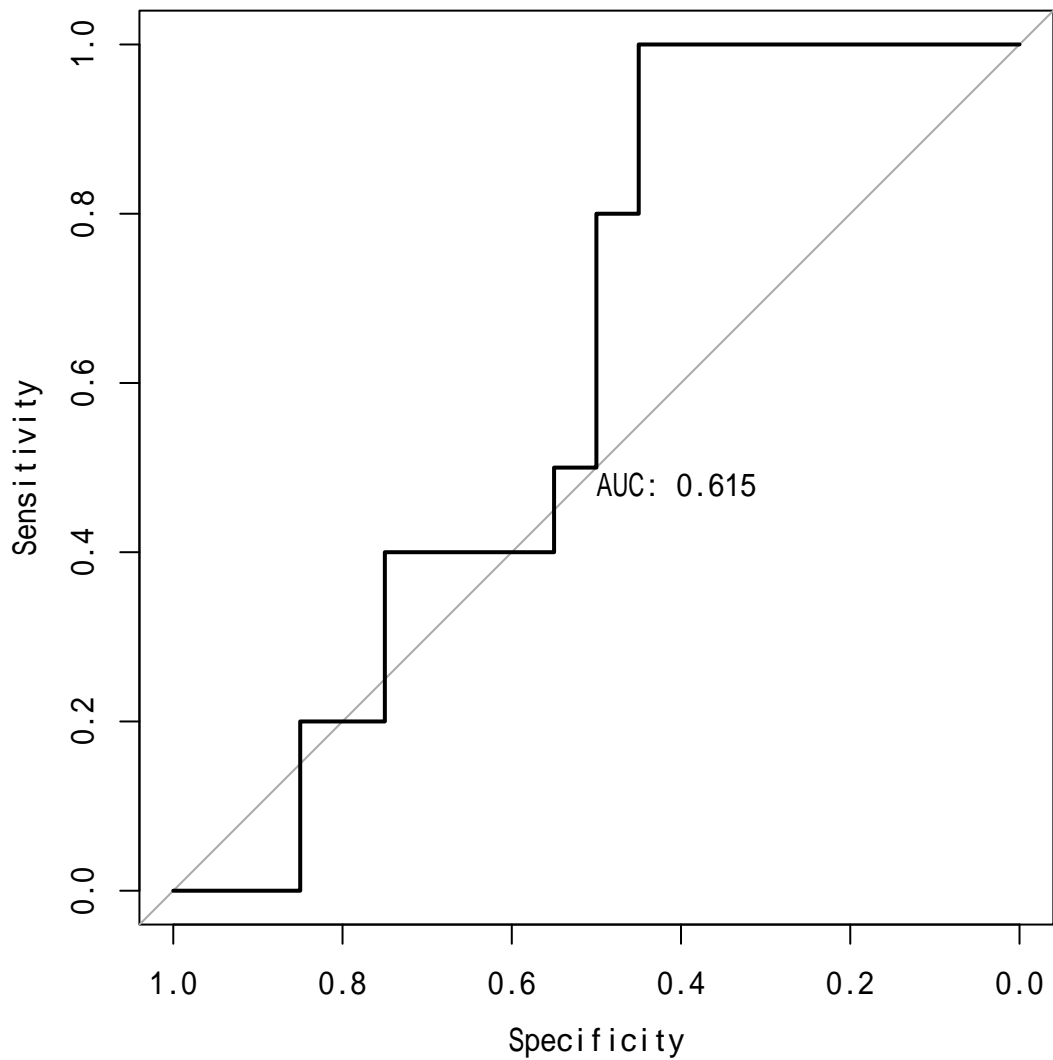

G8

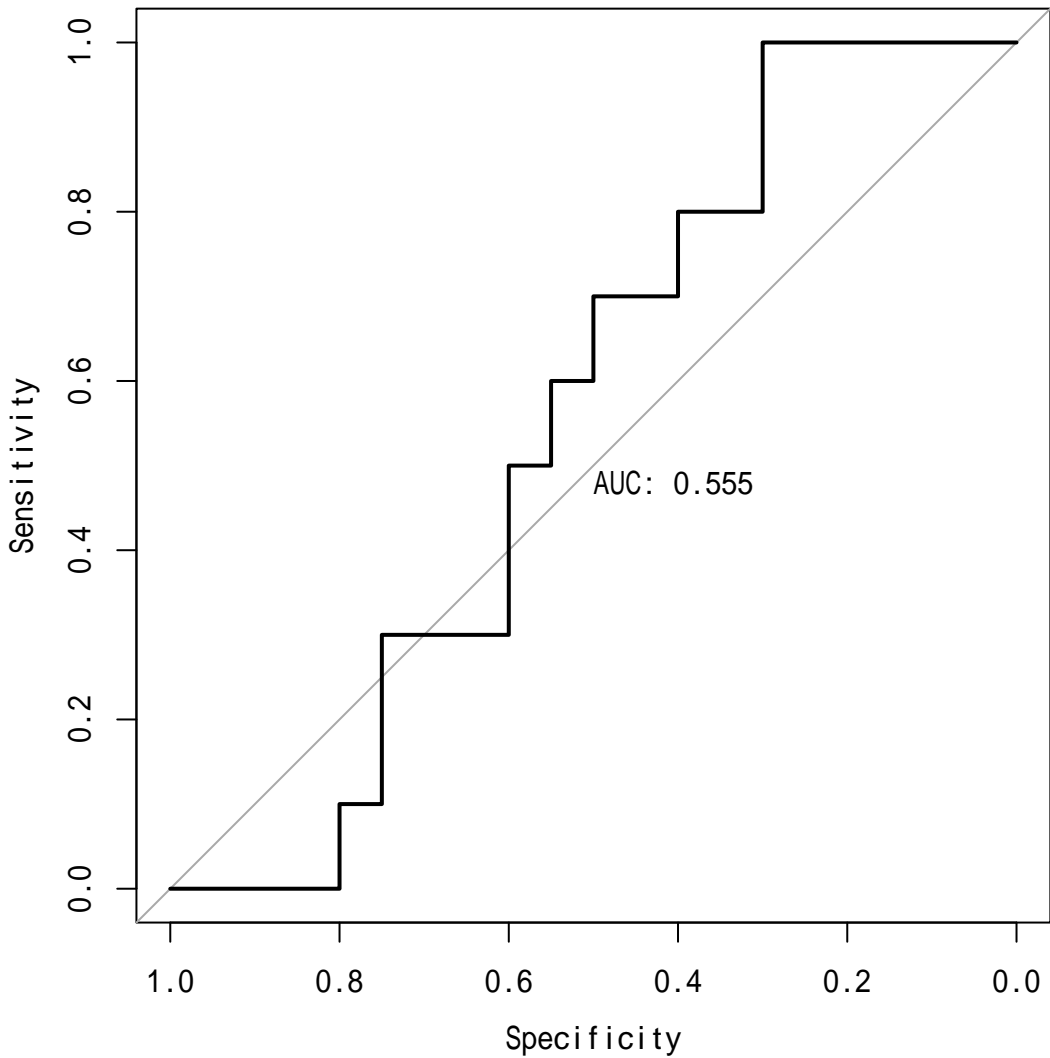

G7

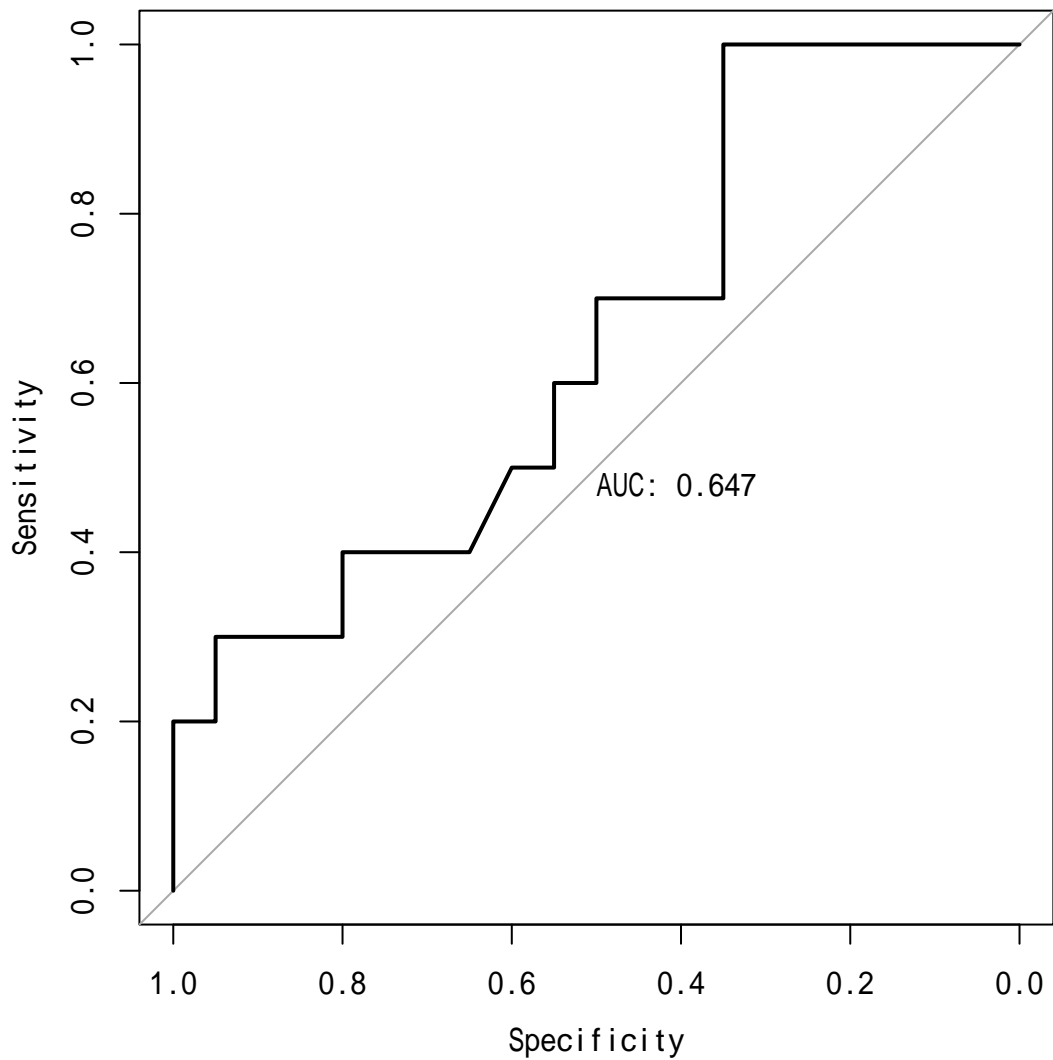

G6

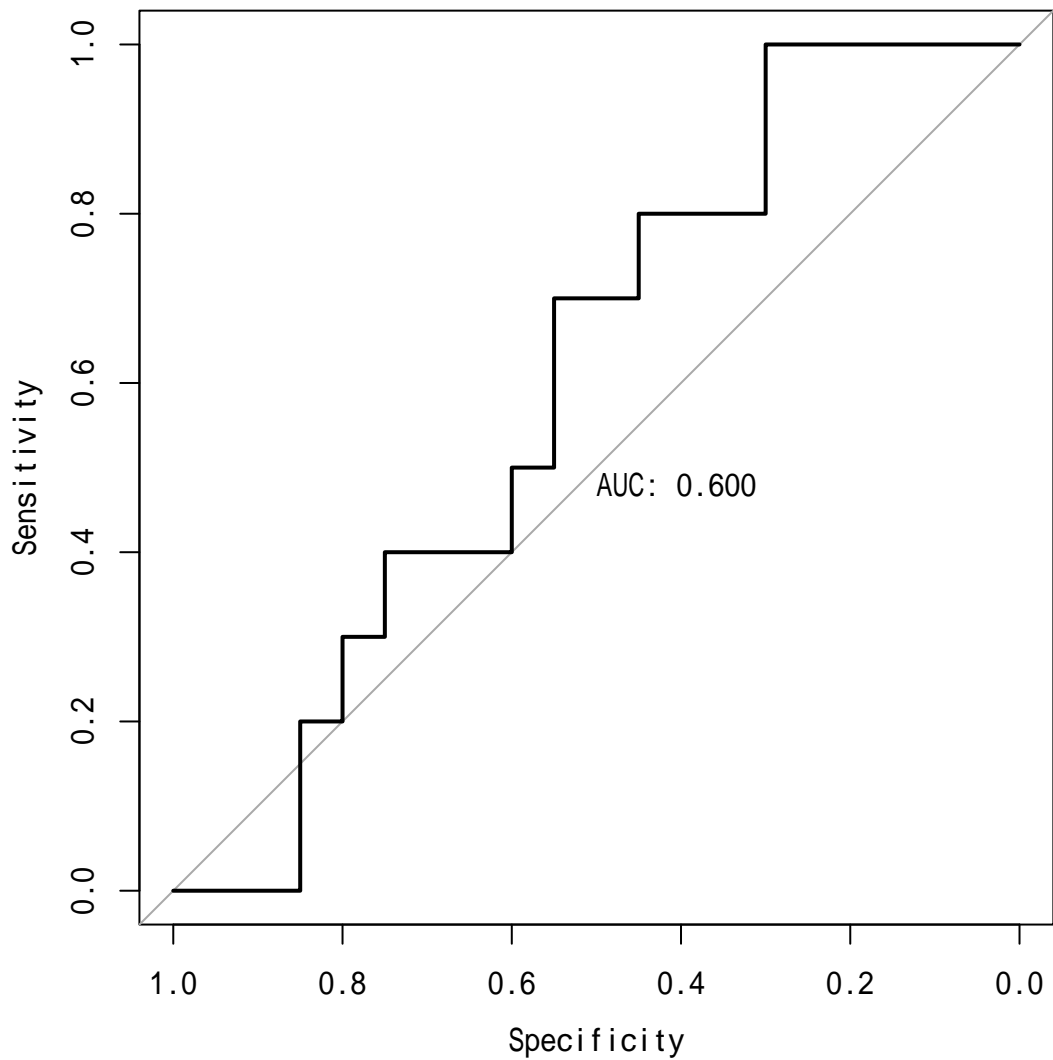

G5

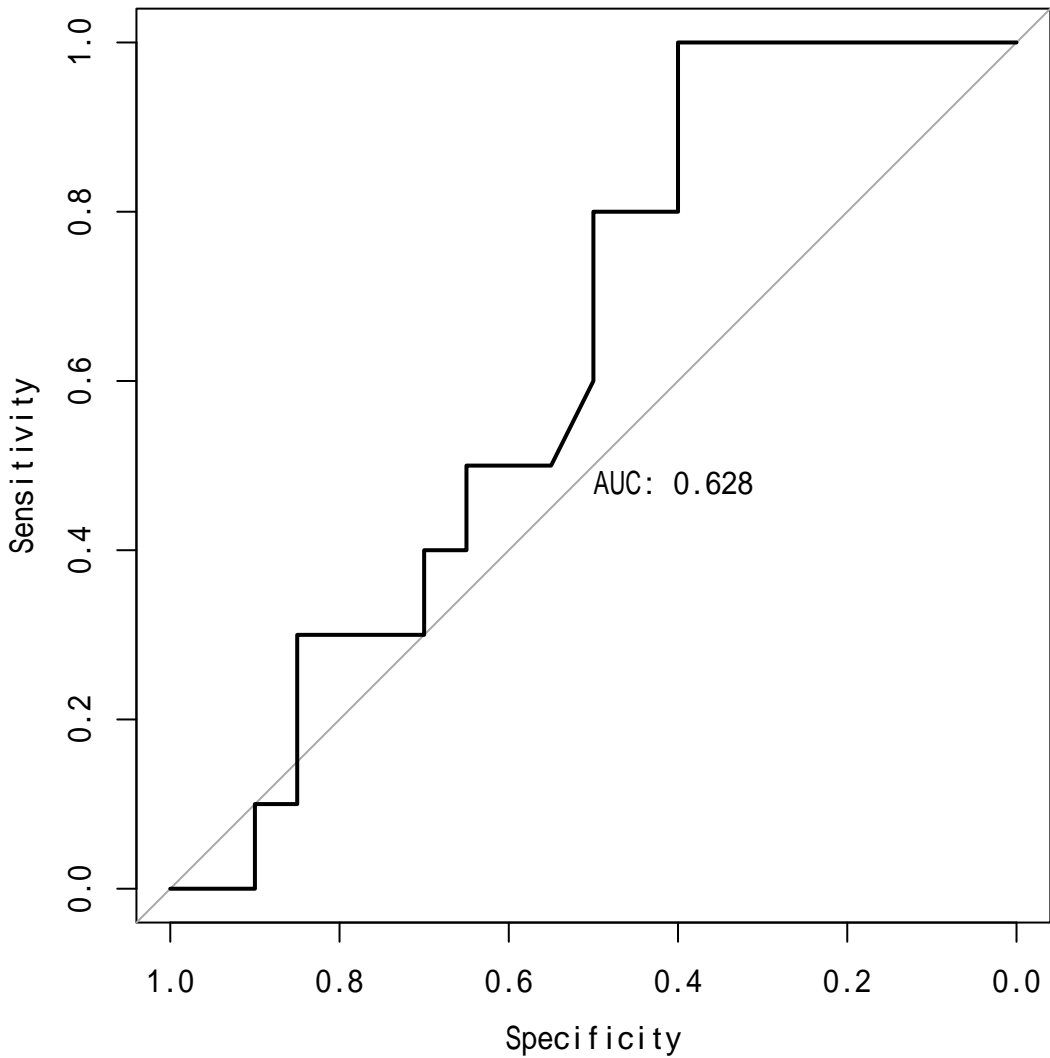

G10

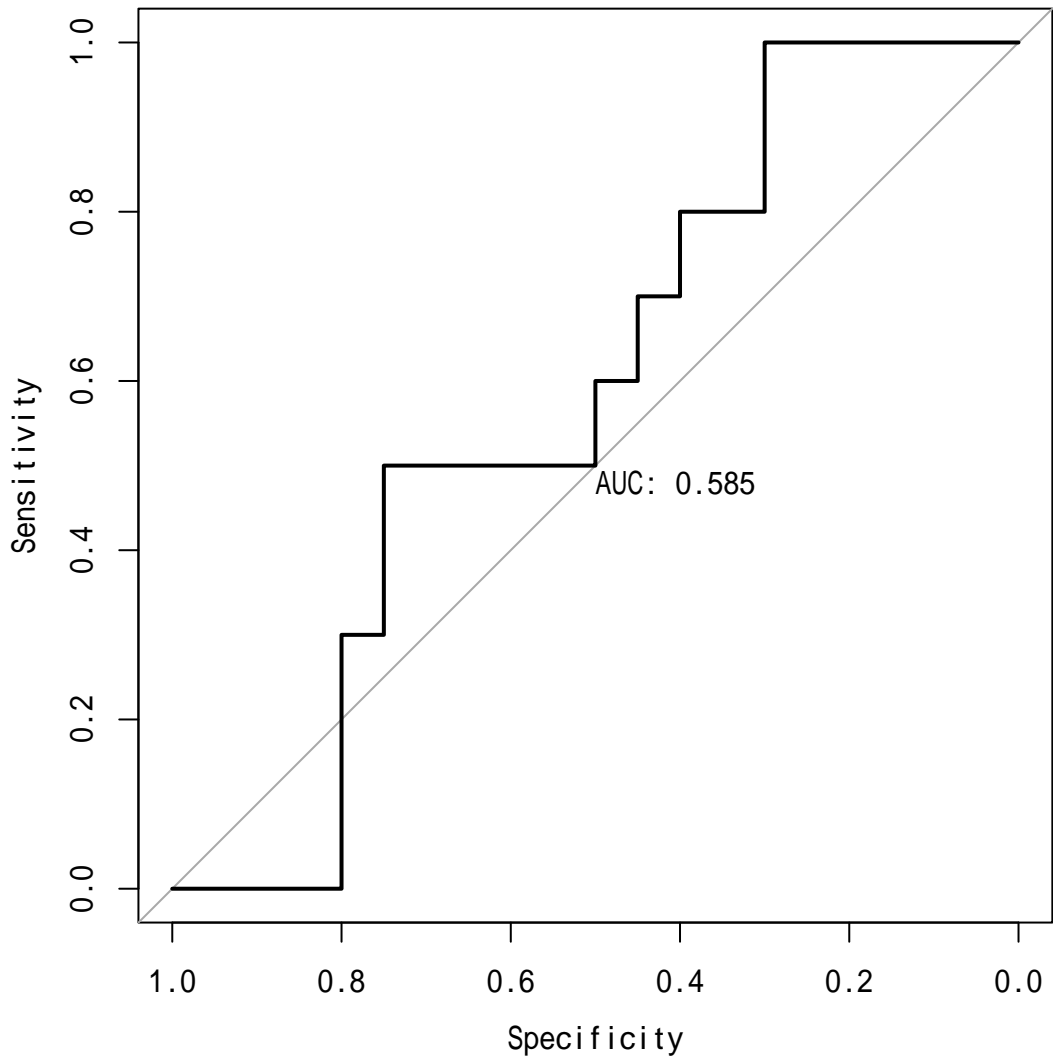

G9

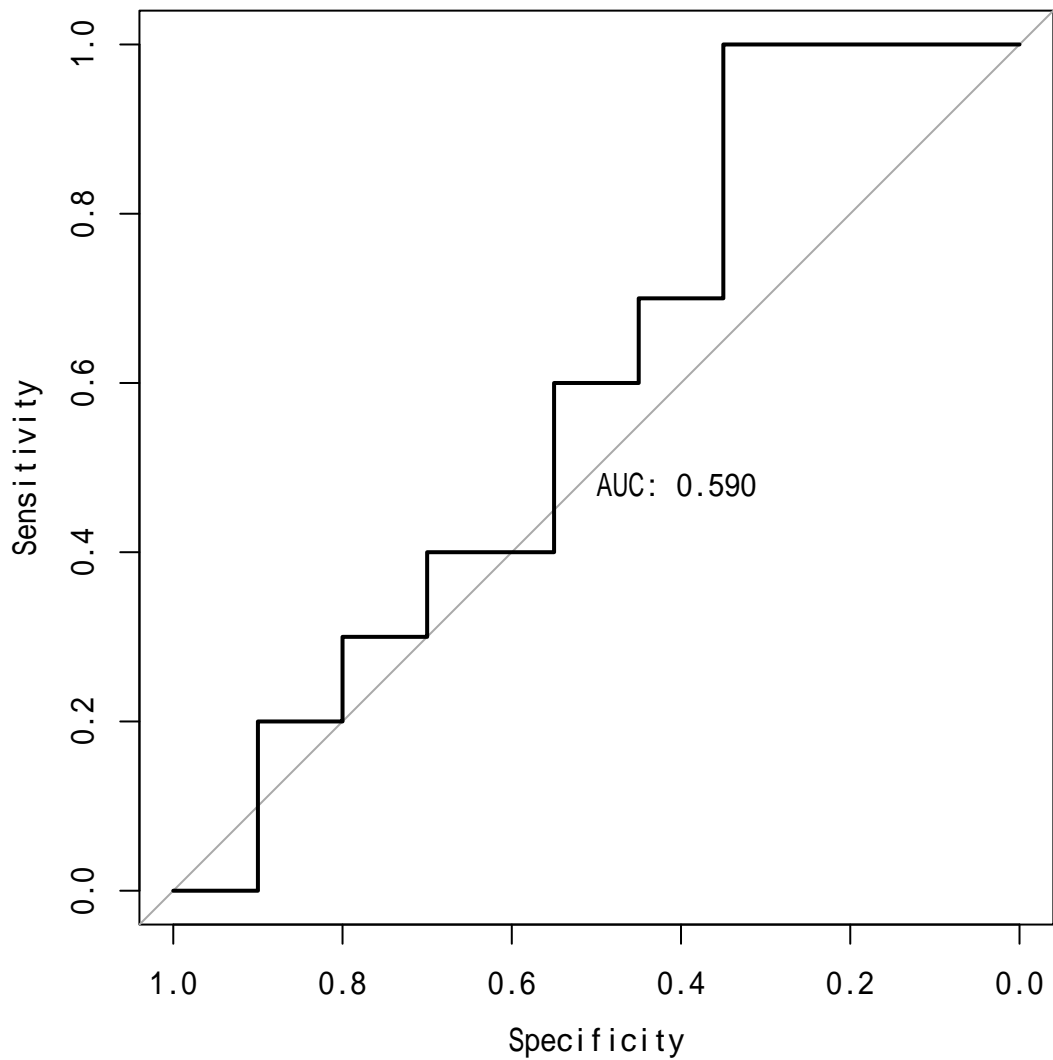

L9

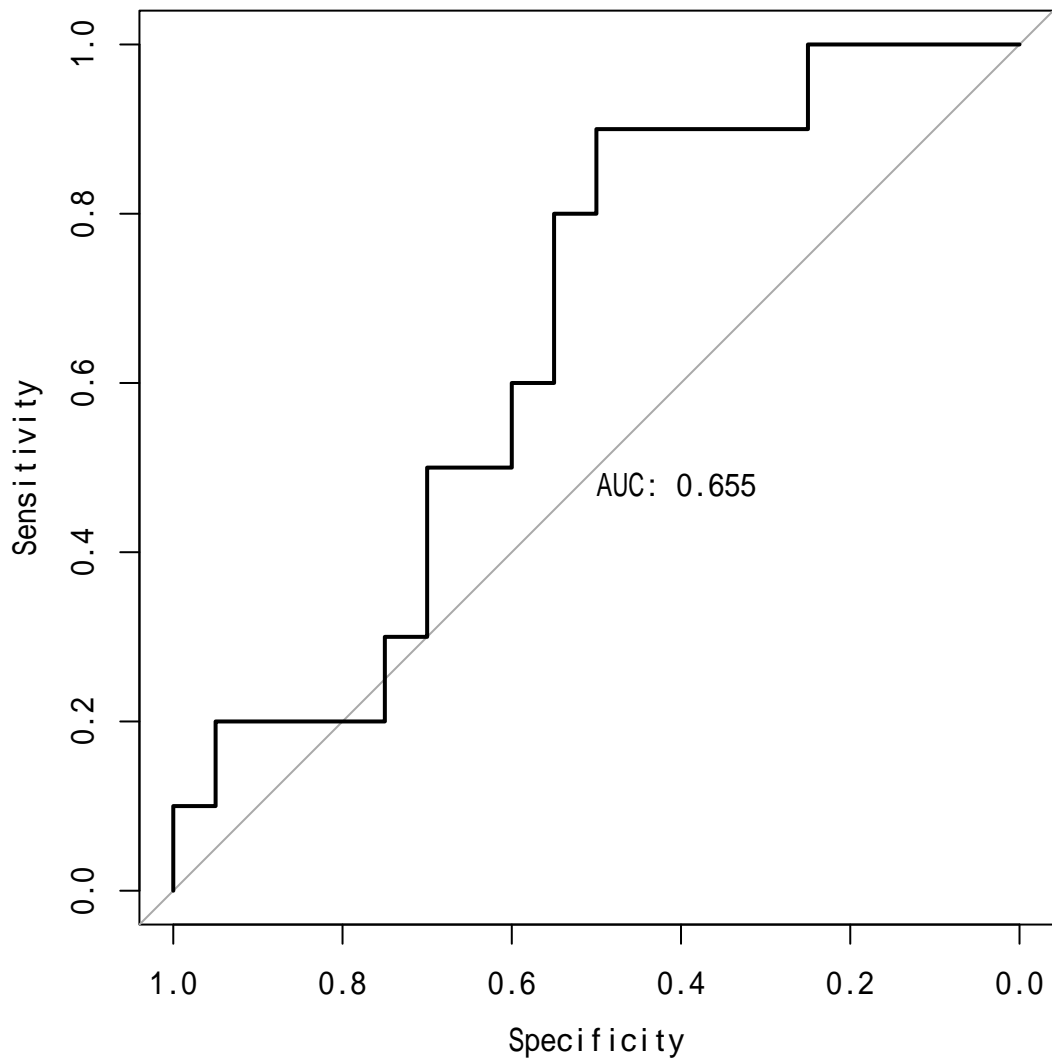

N24

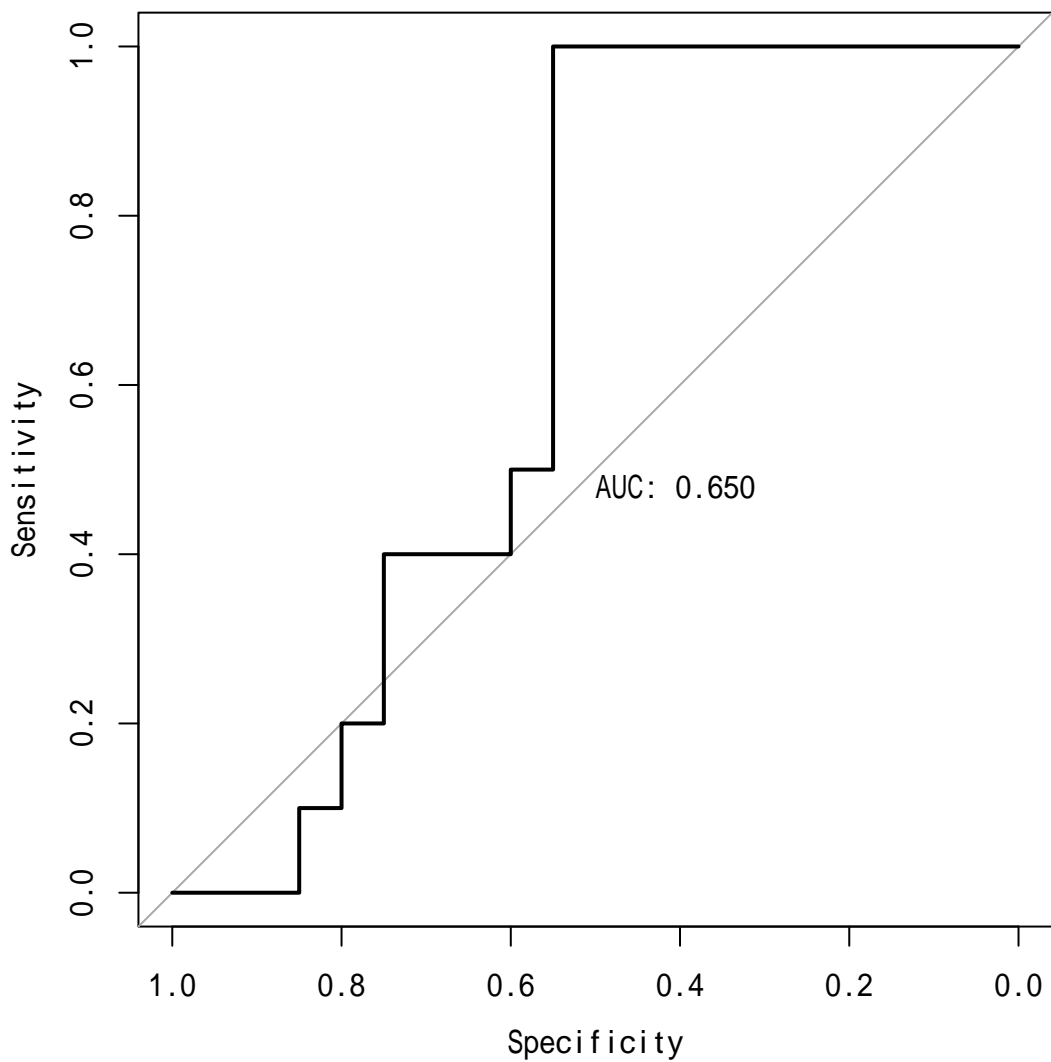

G3

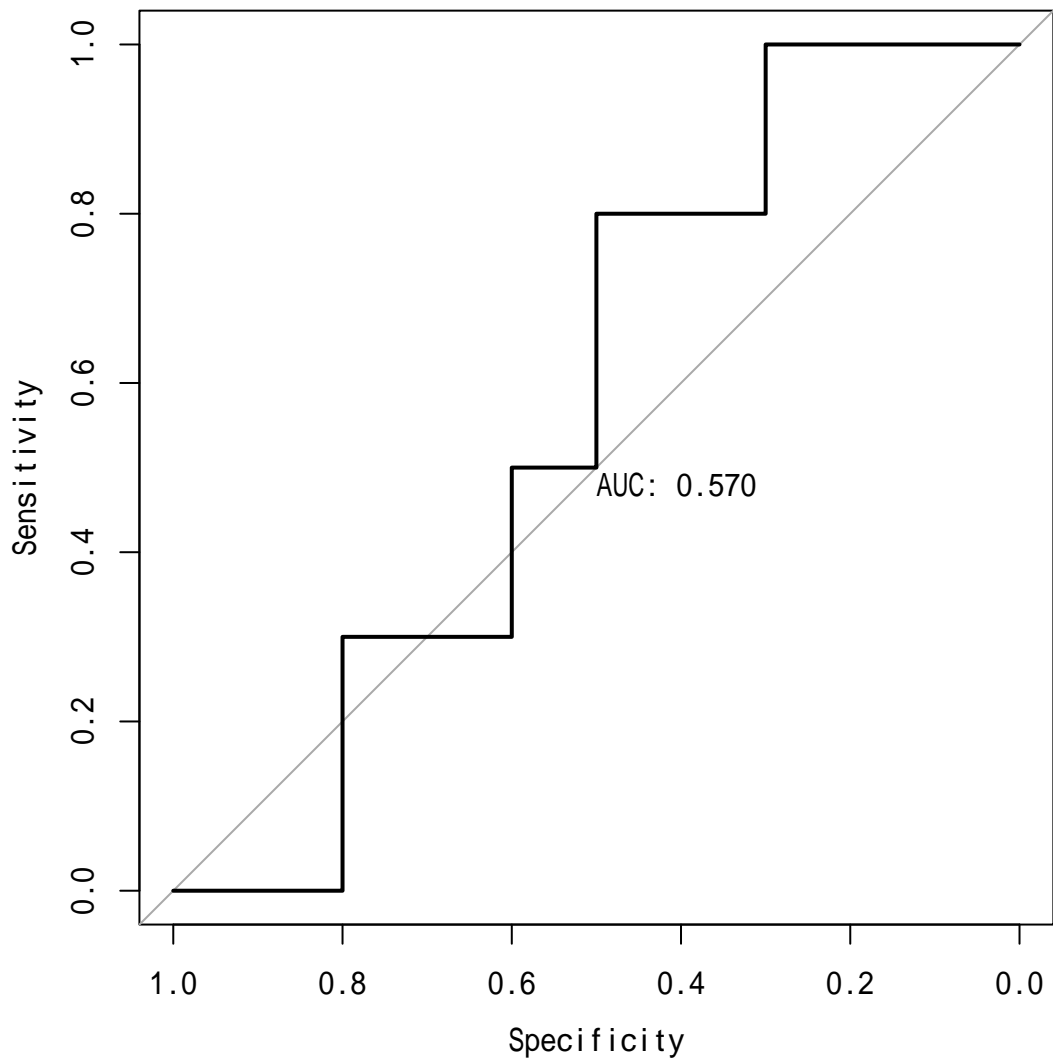

N23

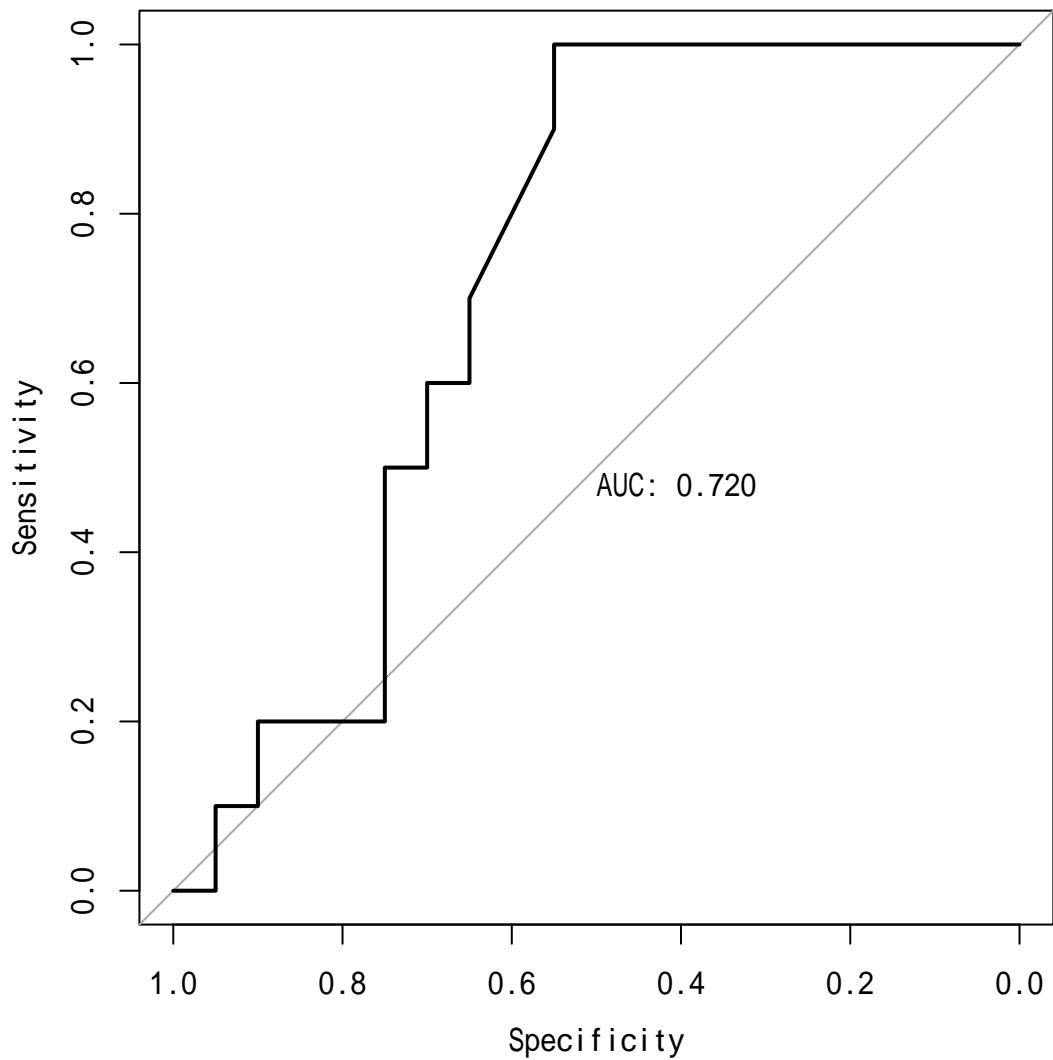

N22

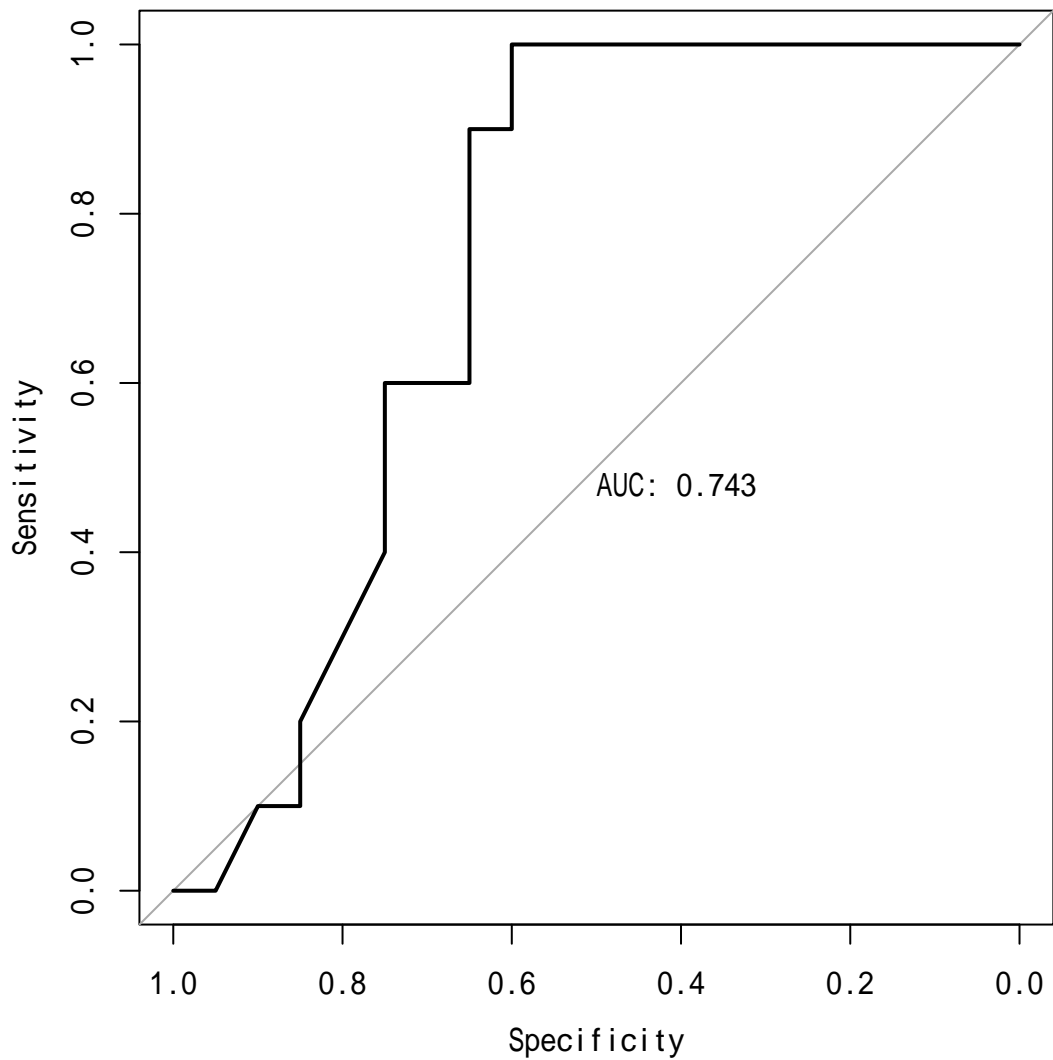

G2

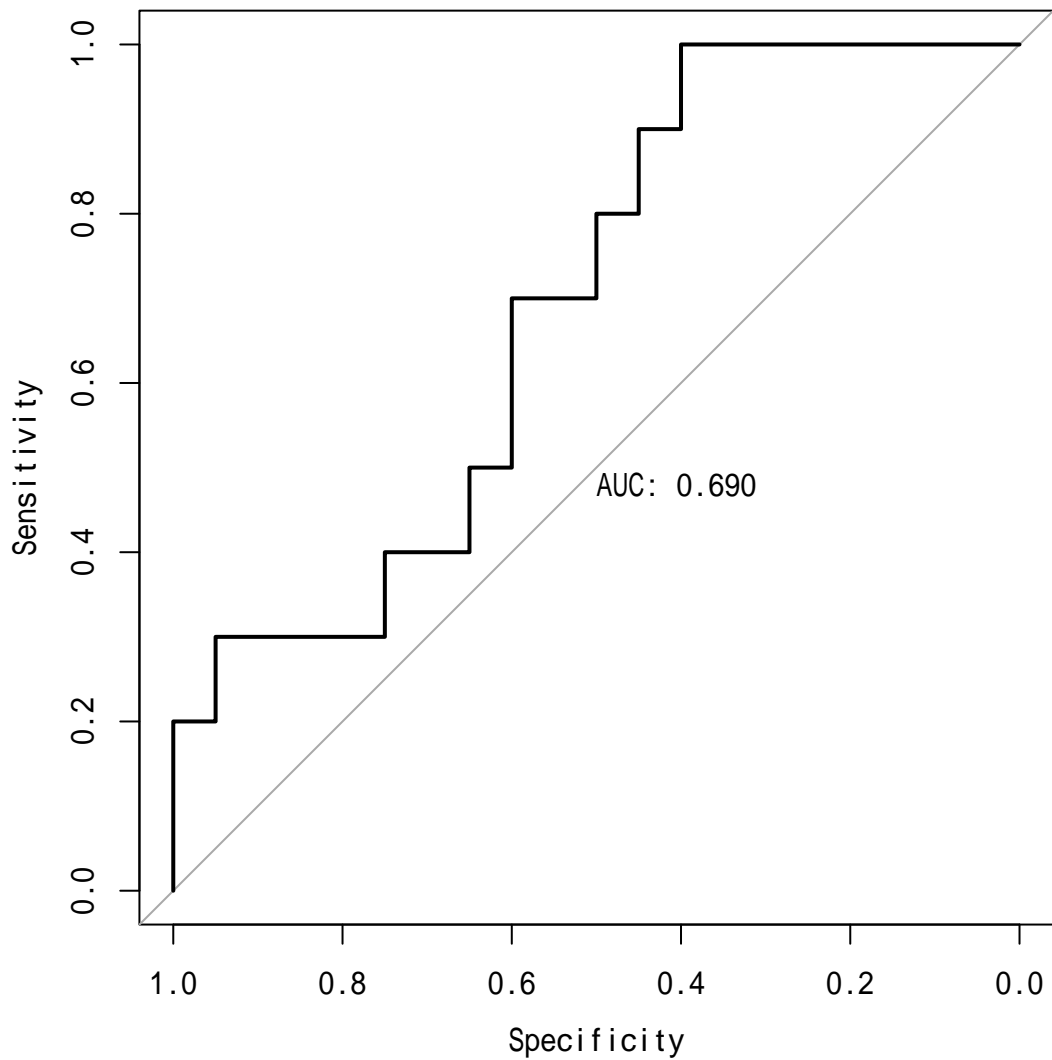

G4

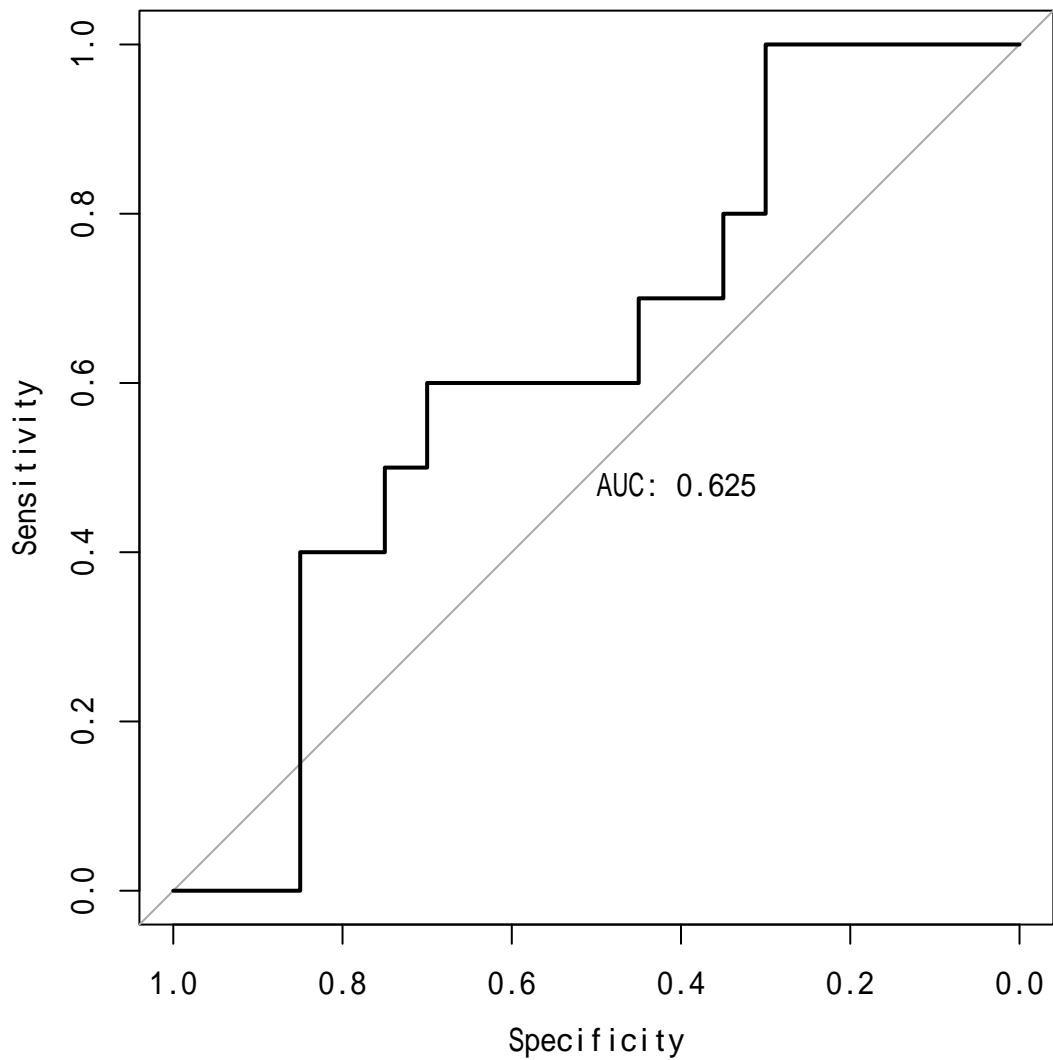

C1

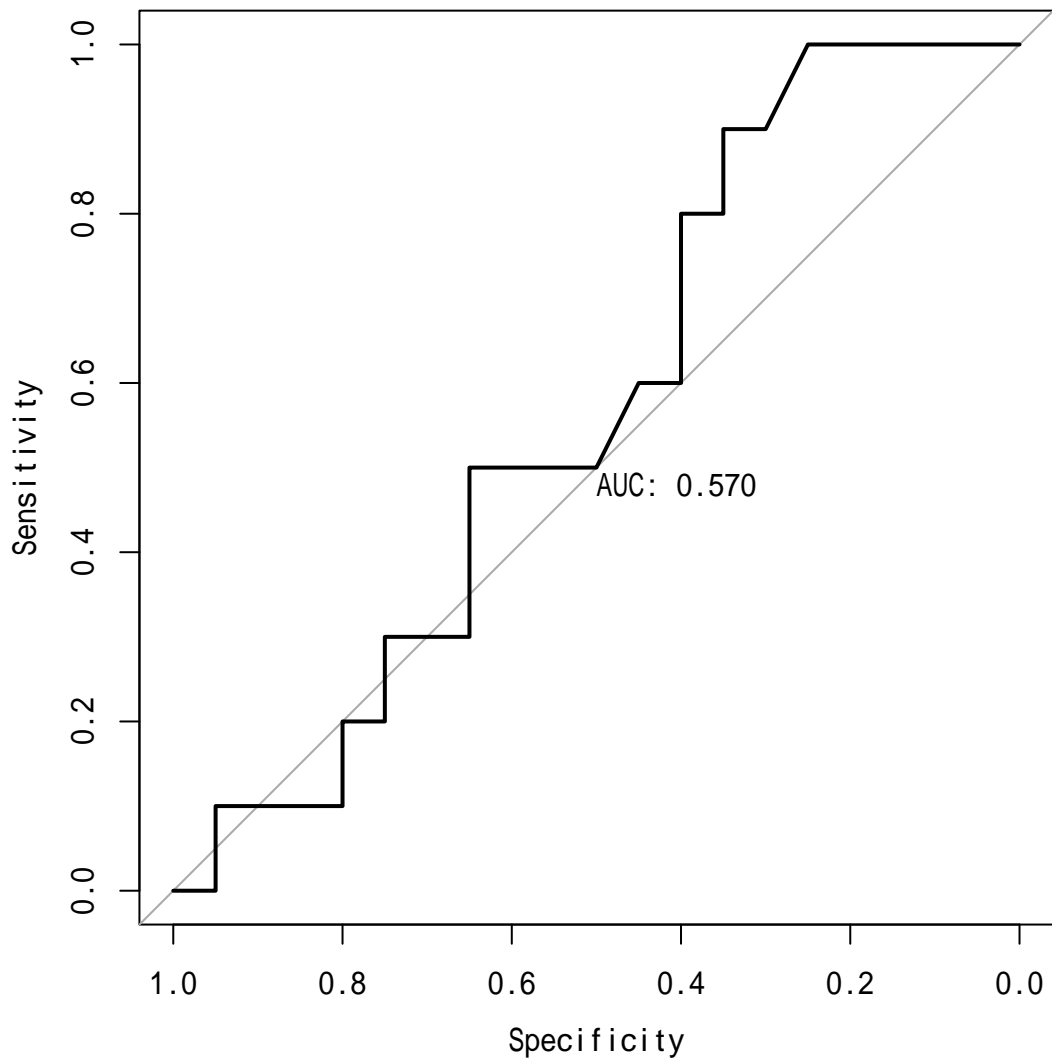

C2

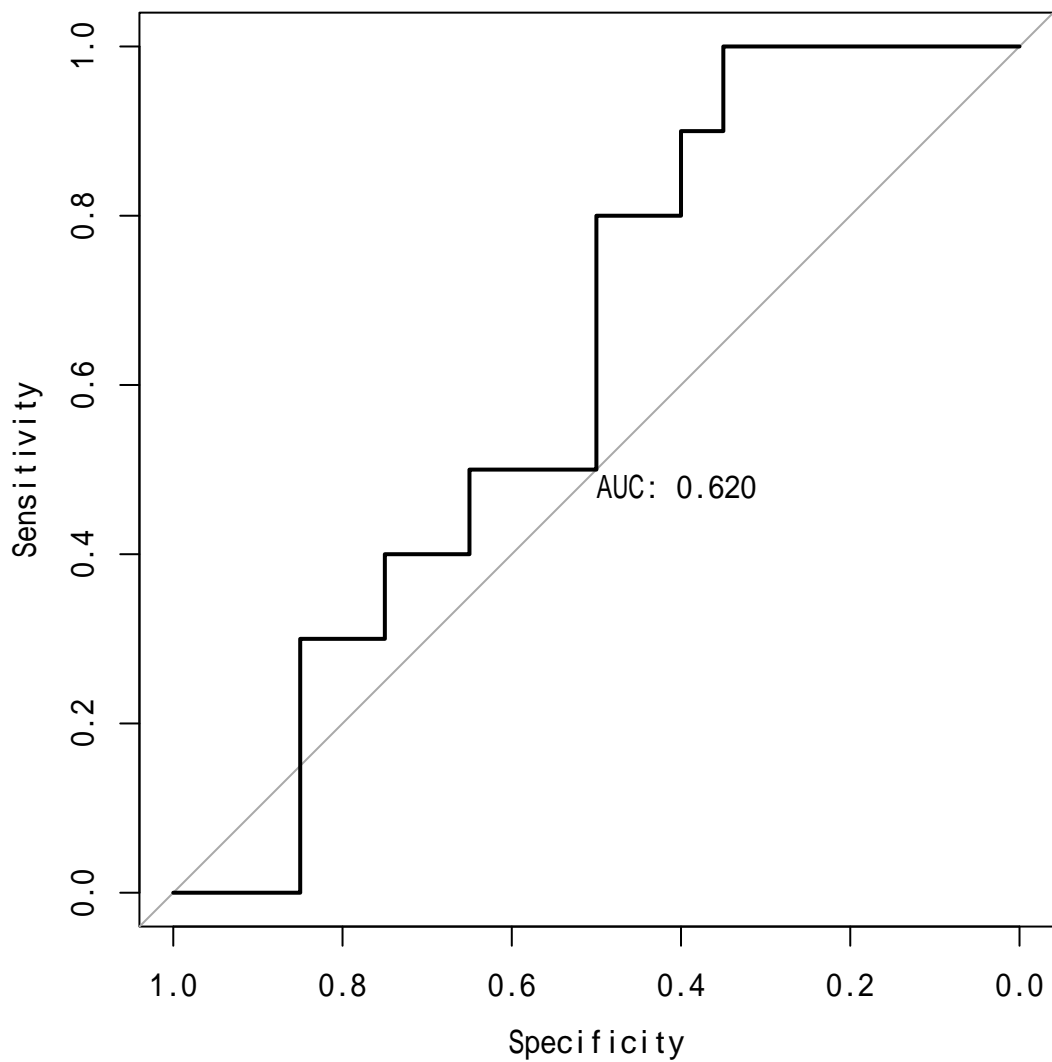

N13

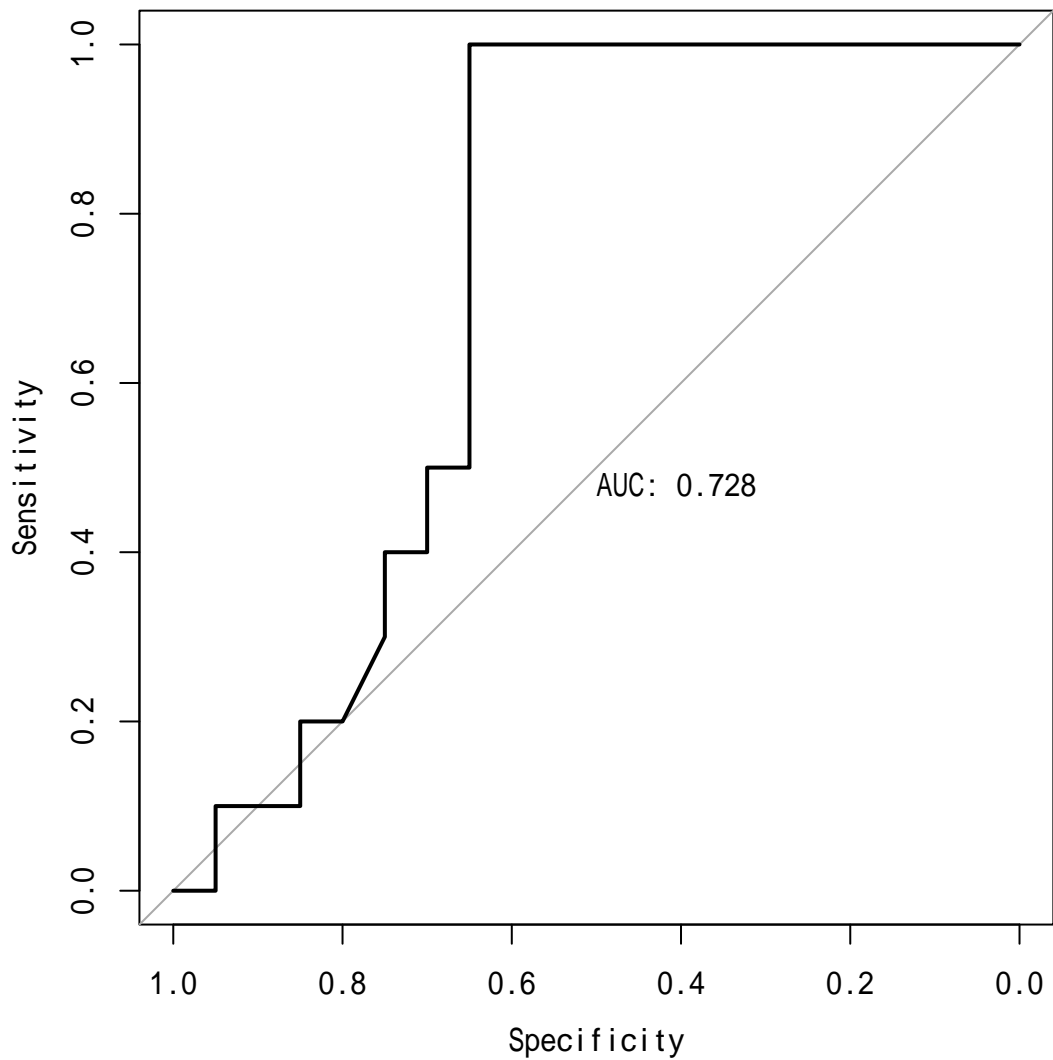

N17

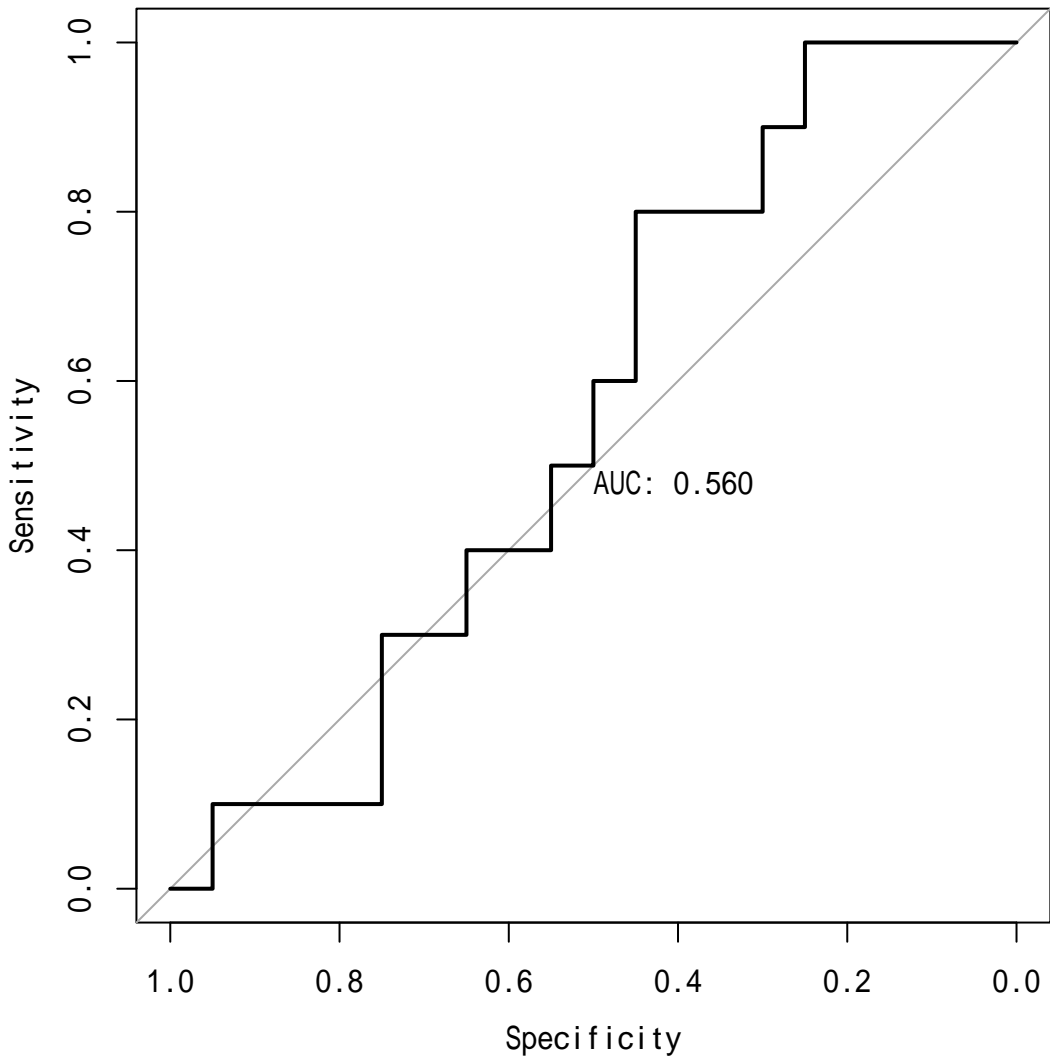

C3

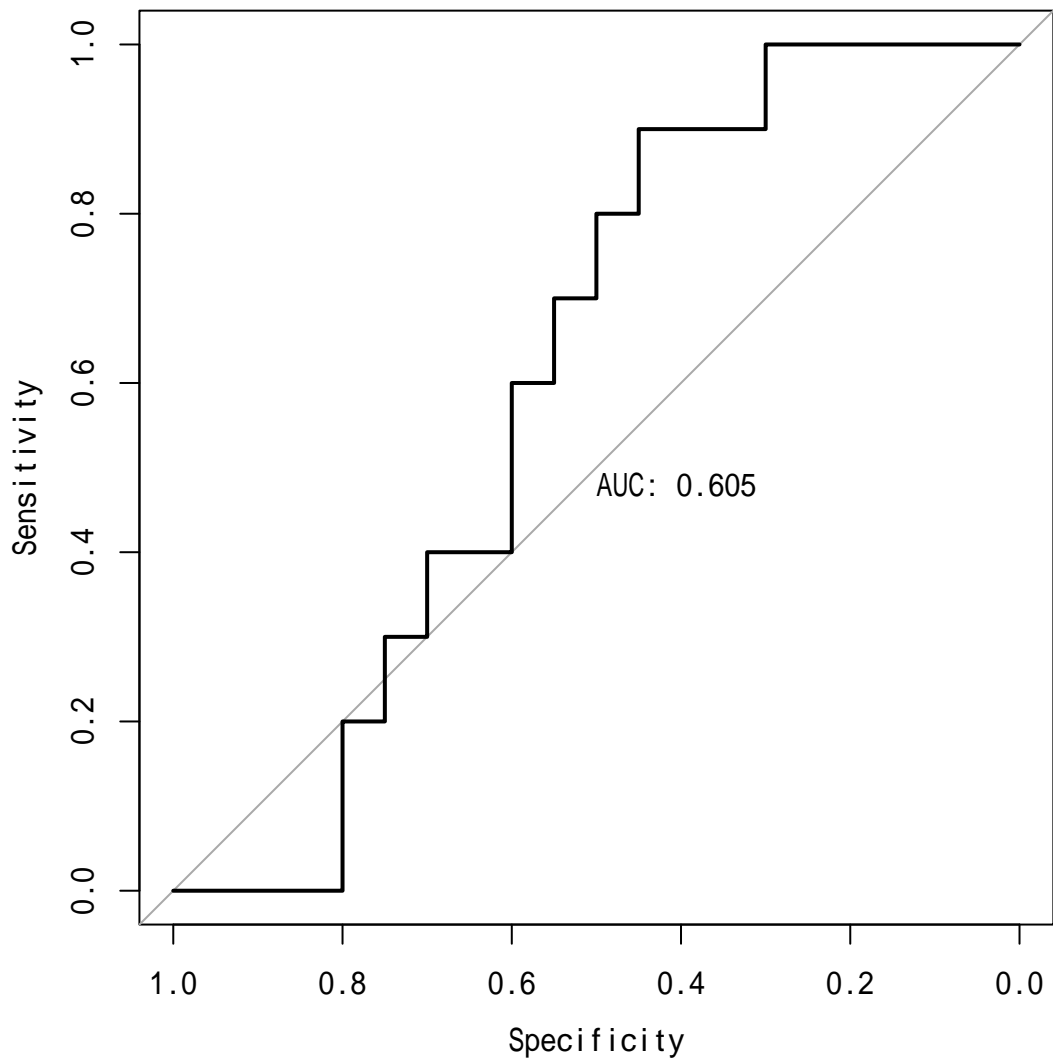

C4

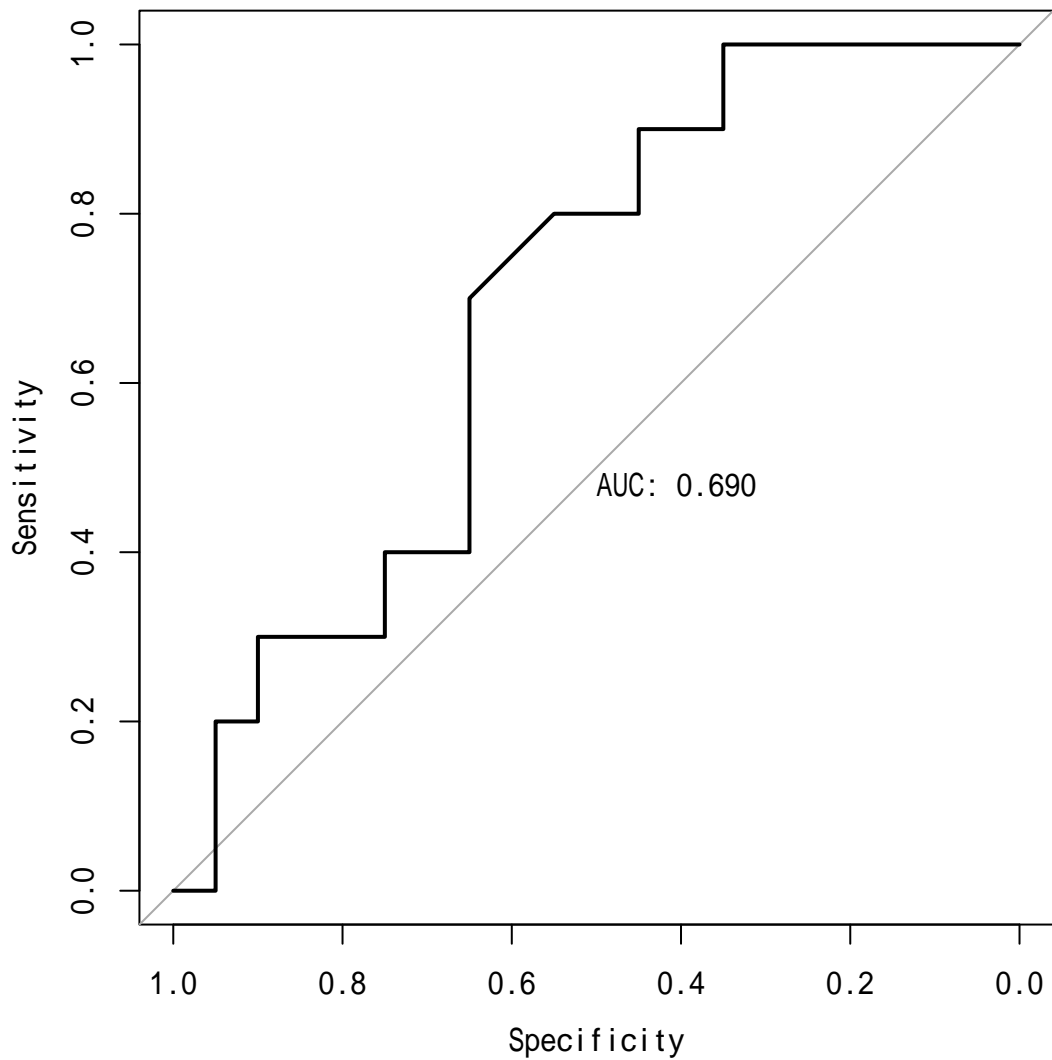

N10

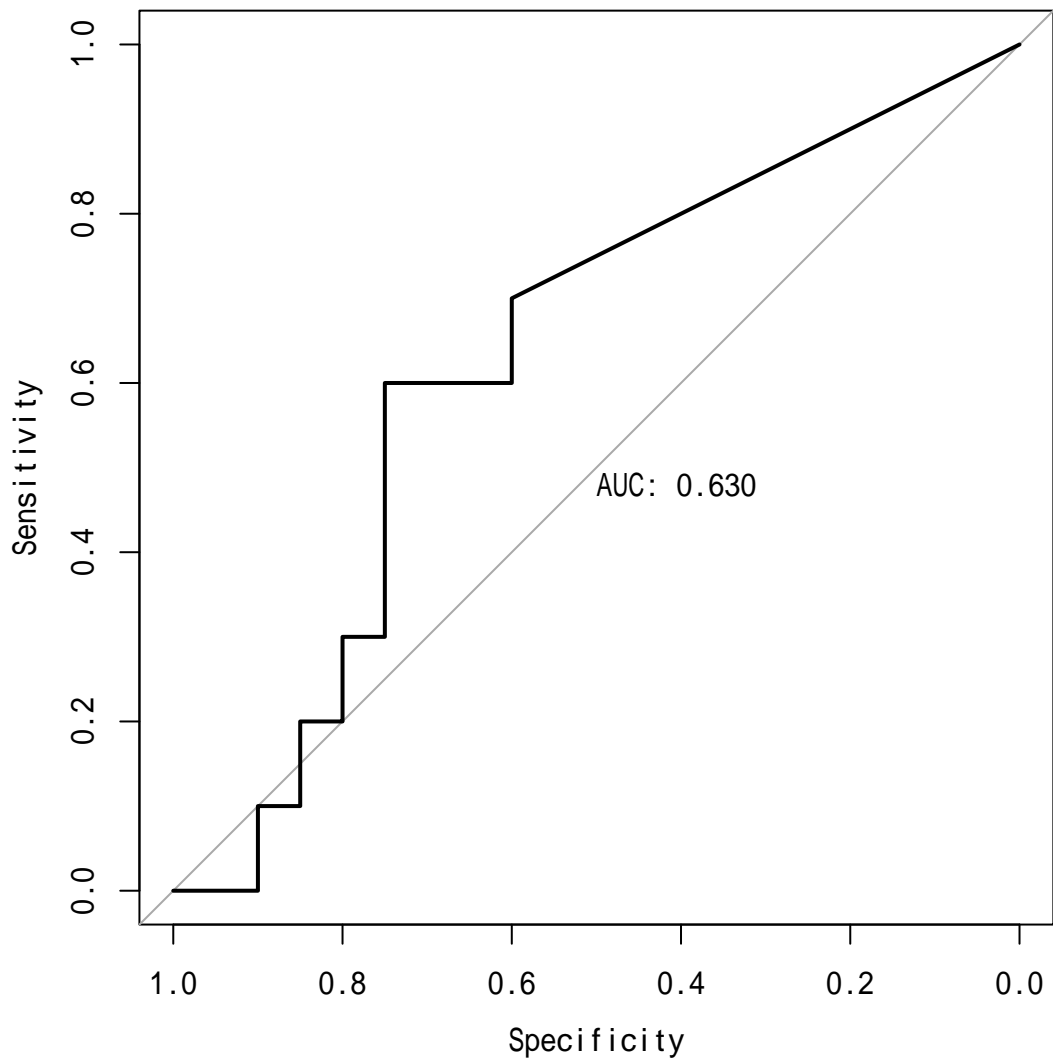

N16

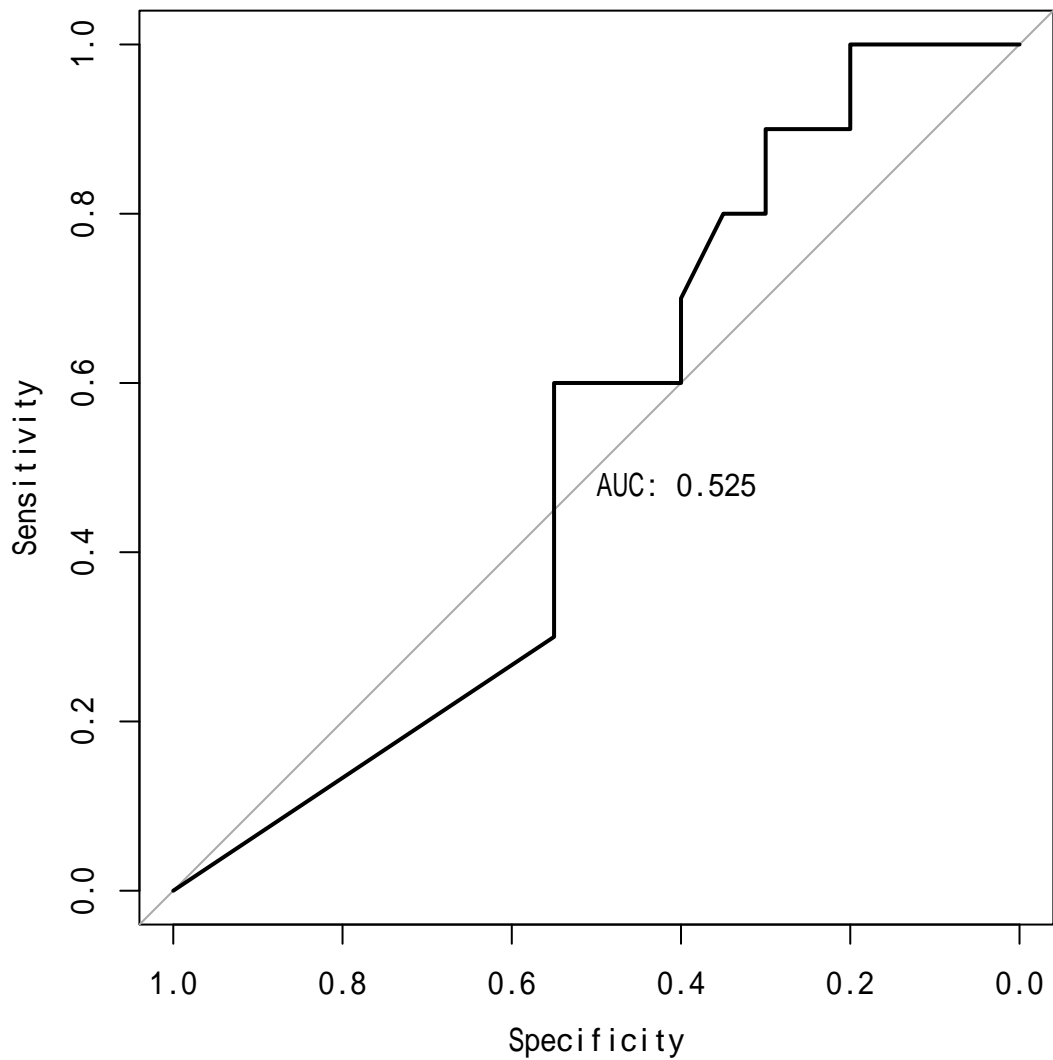

L1

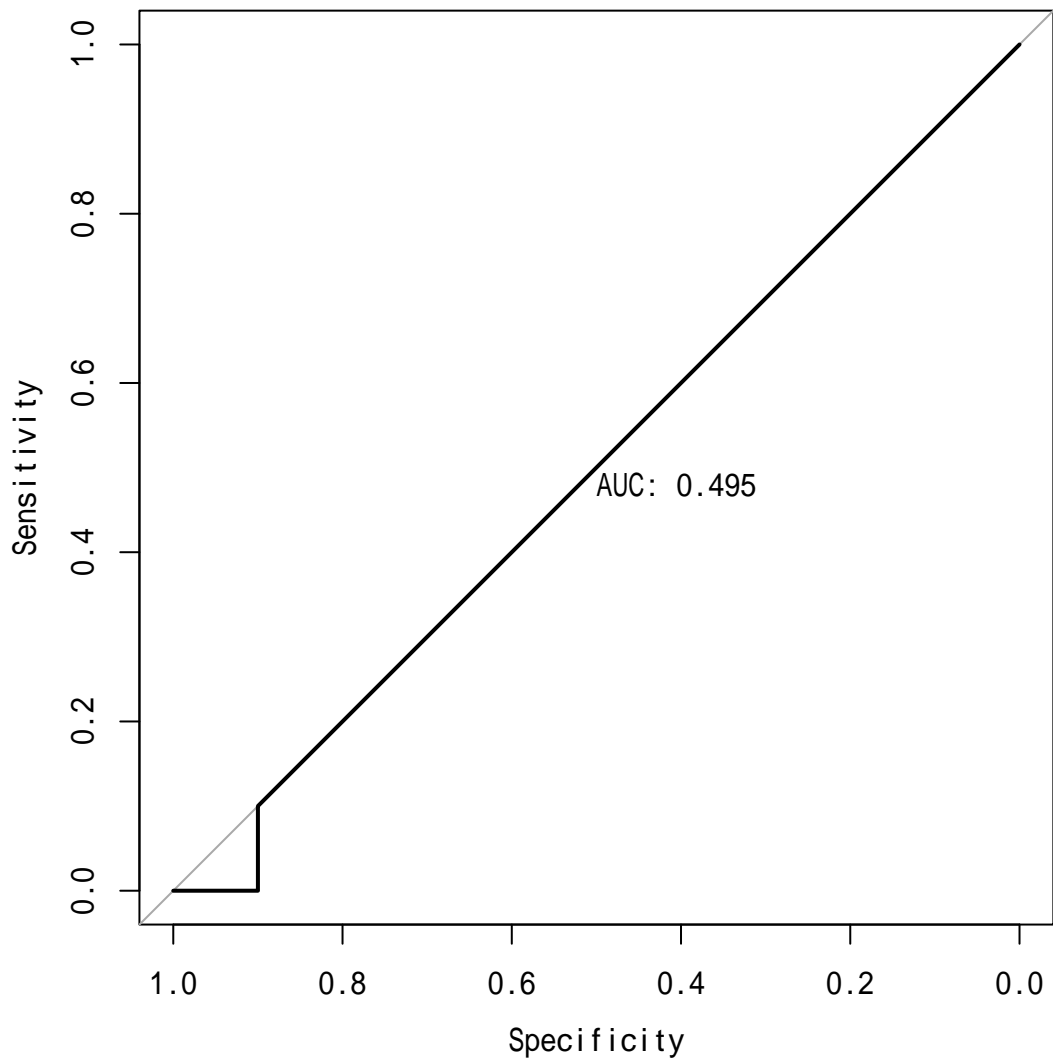

N18

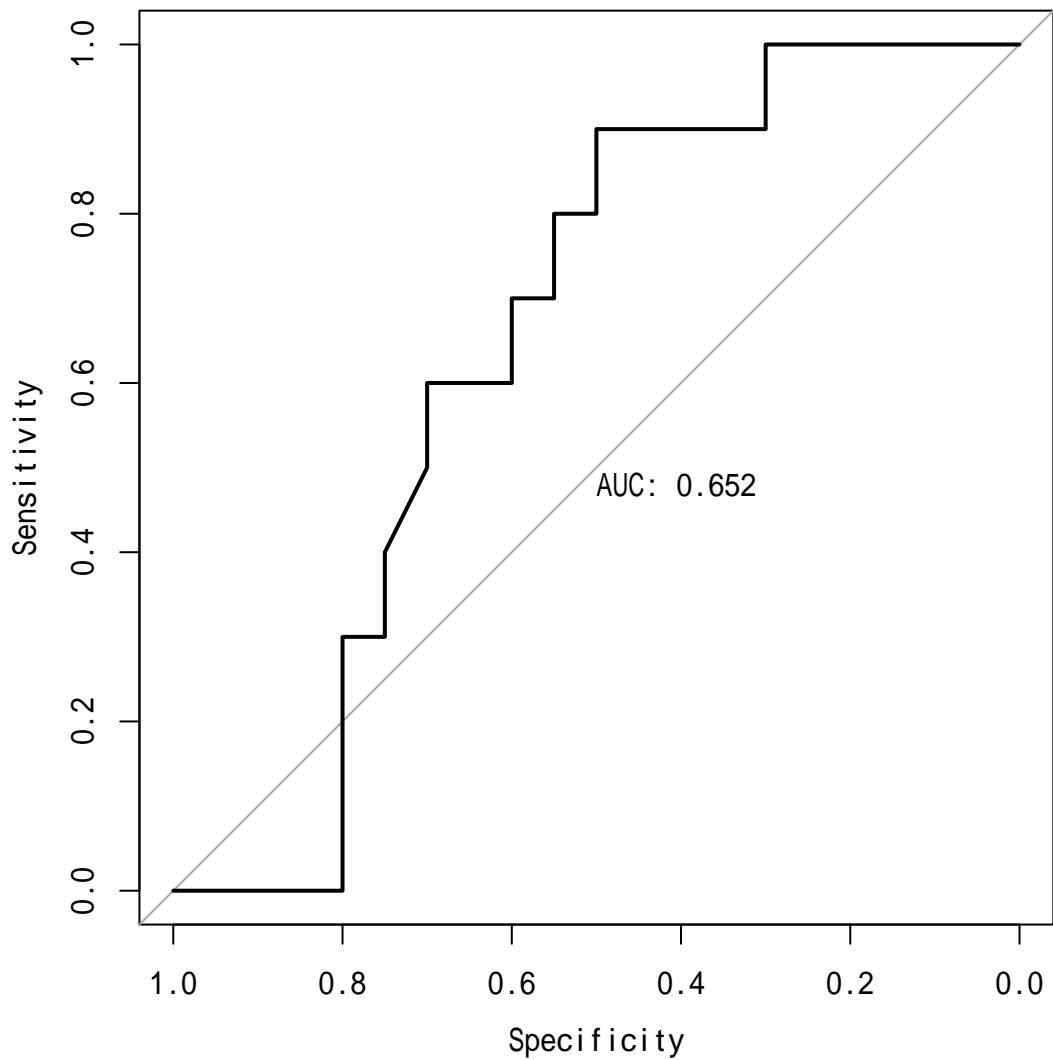

F1

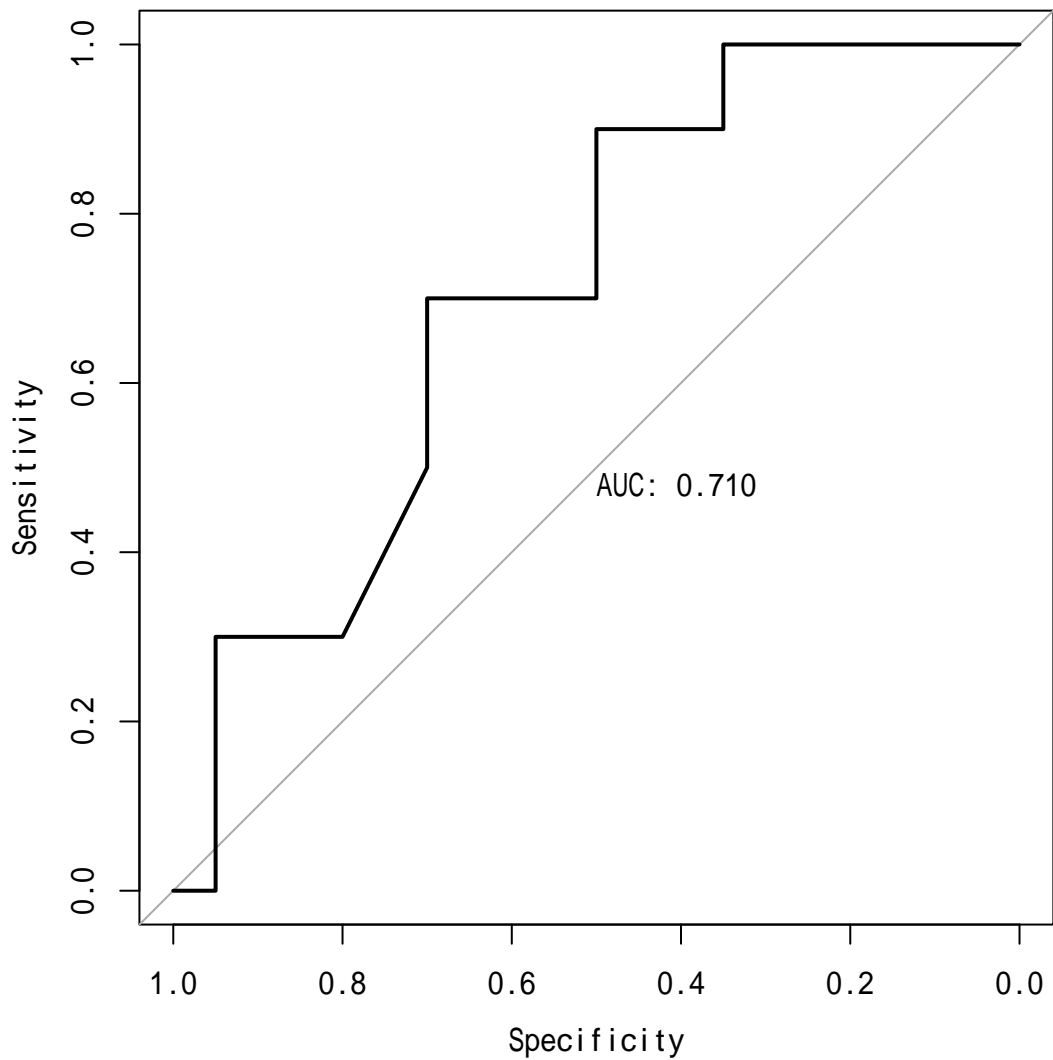

F7

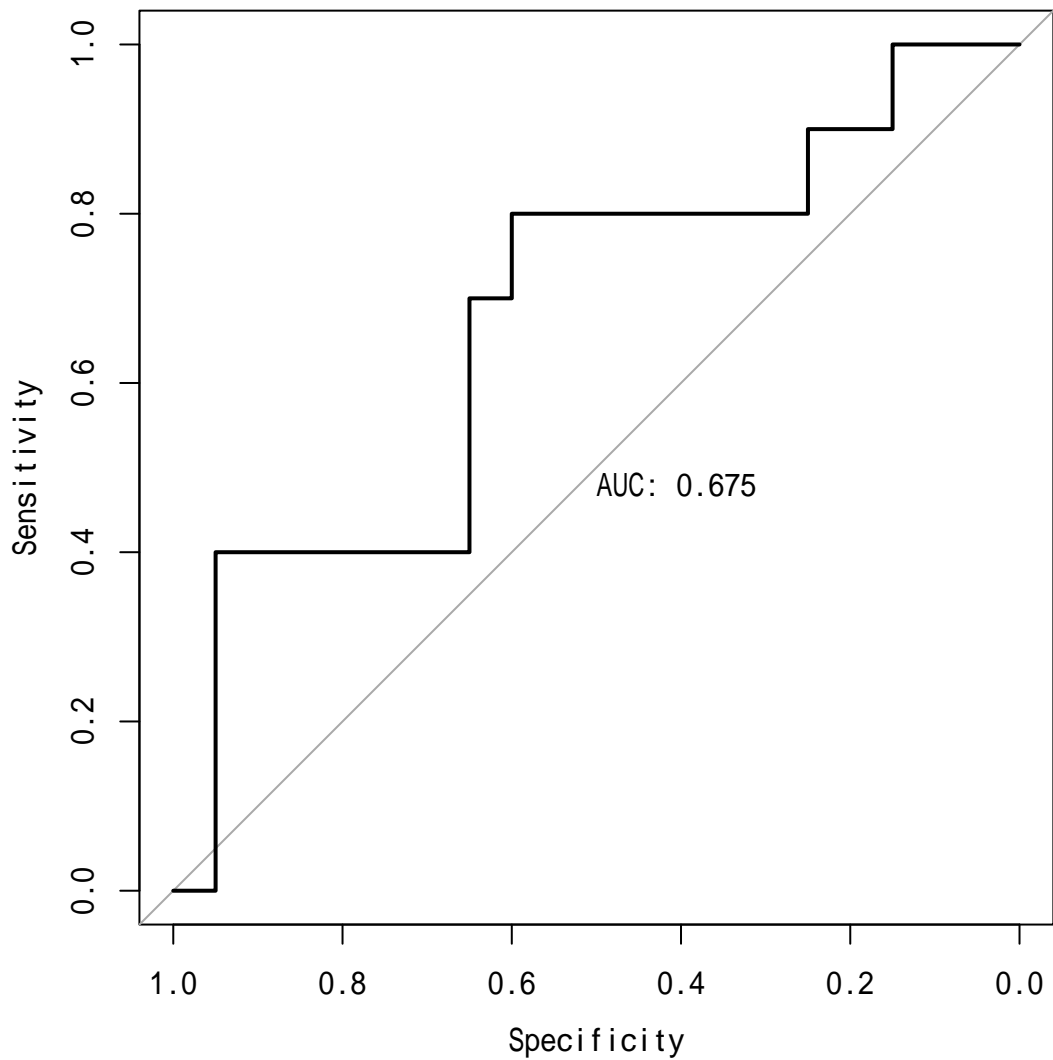

N29

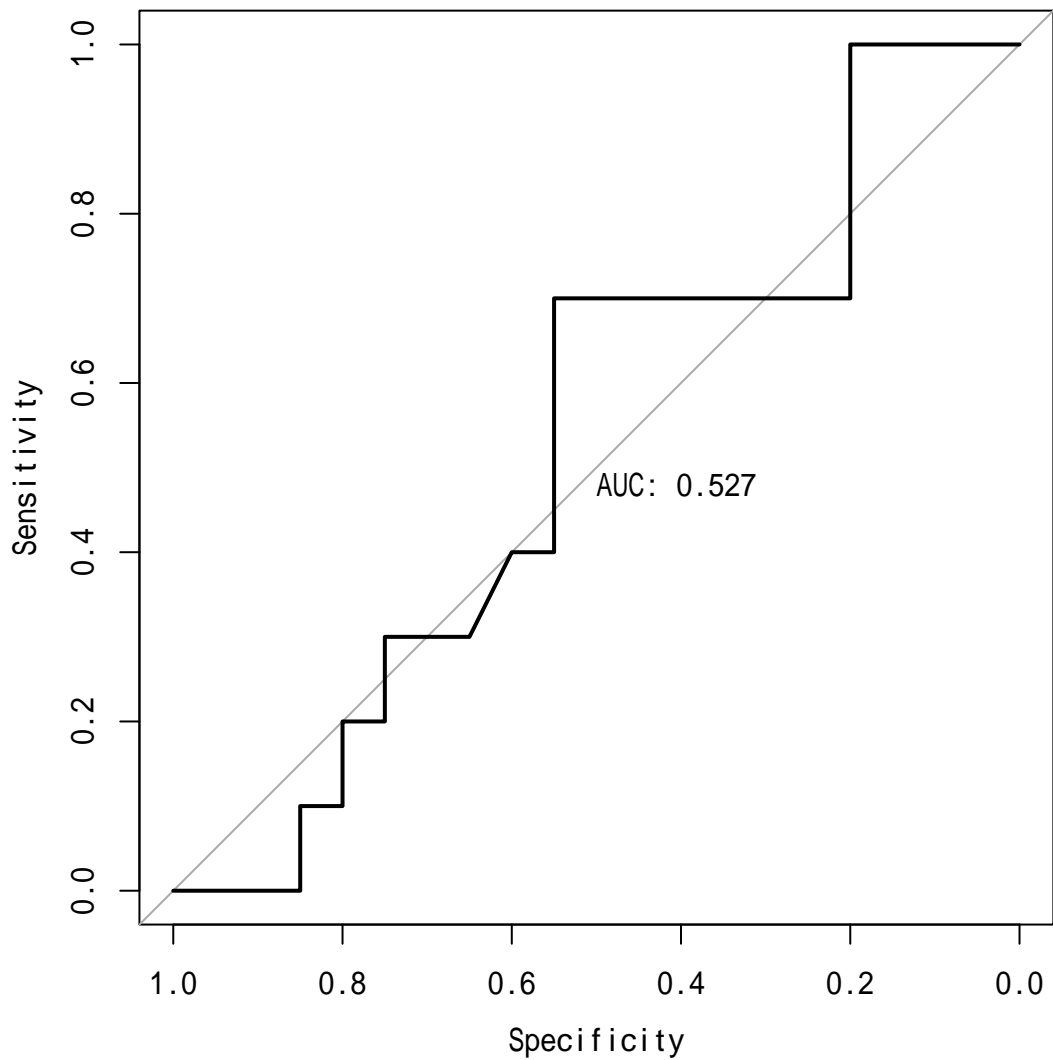

N6

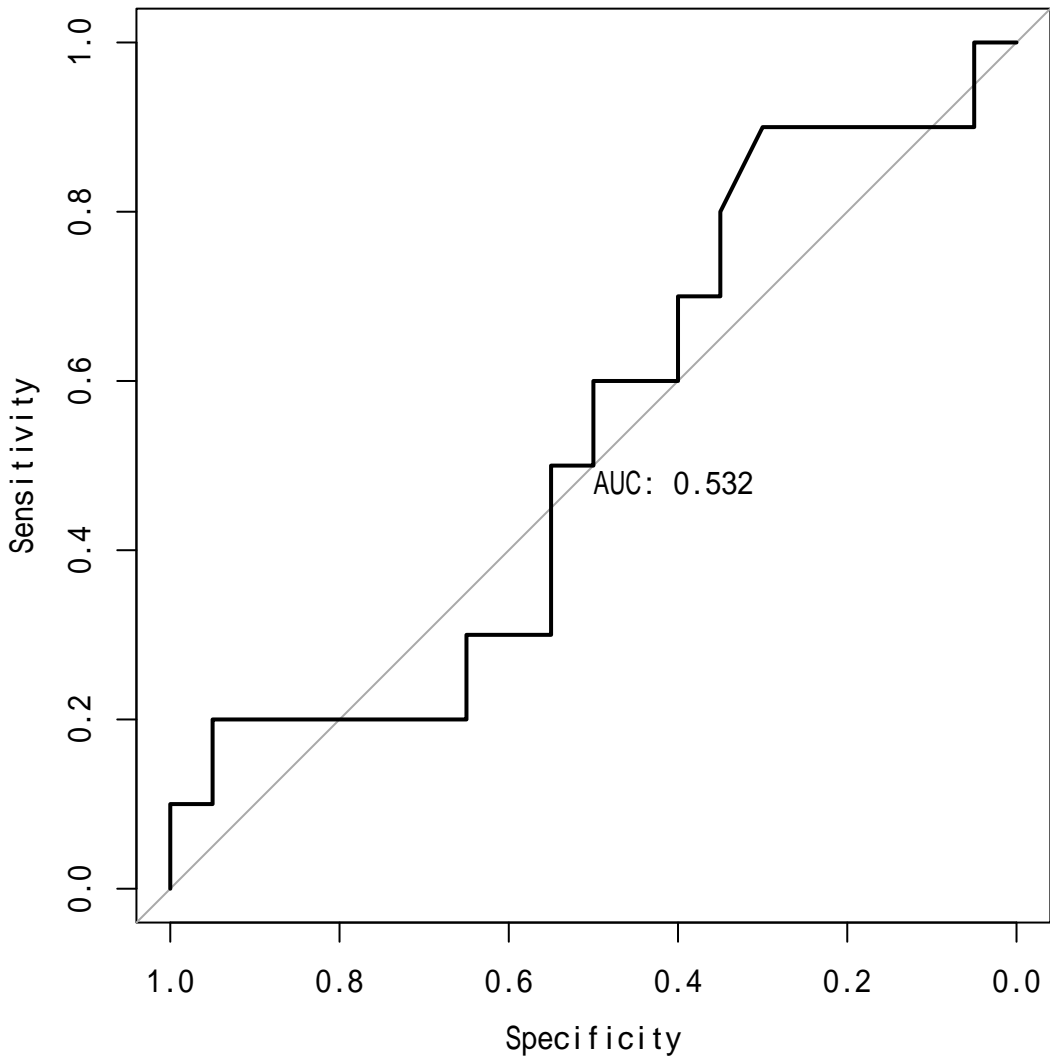

B2

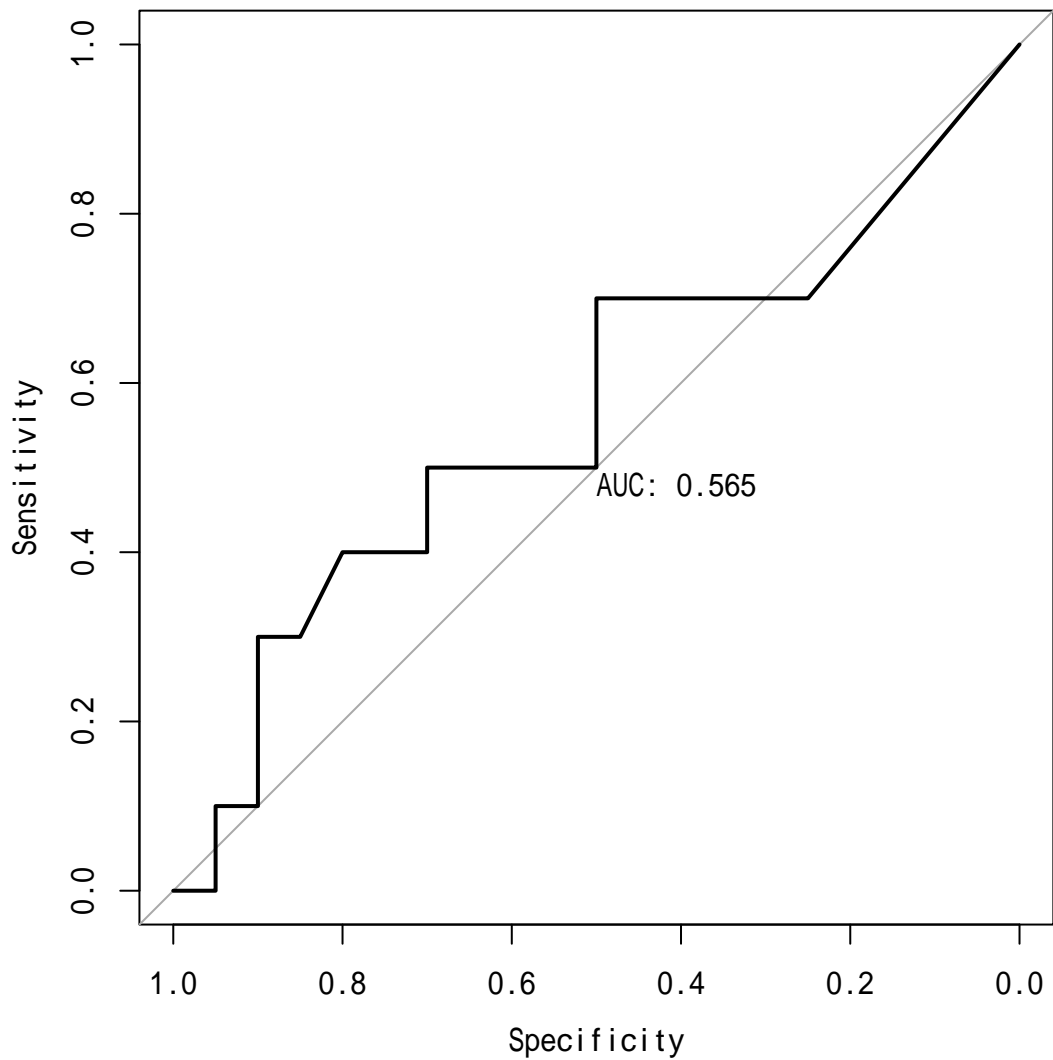

F5

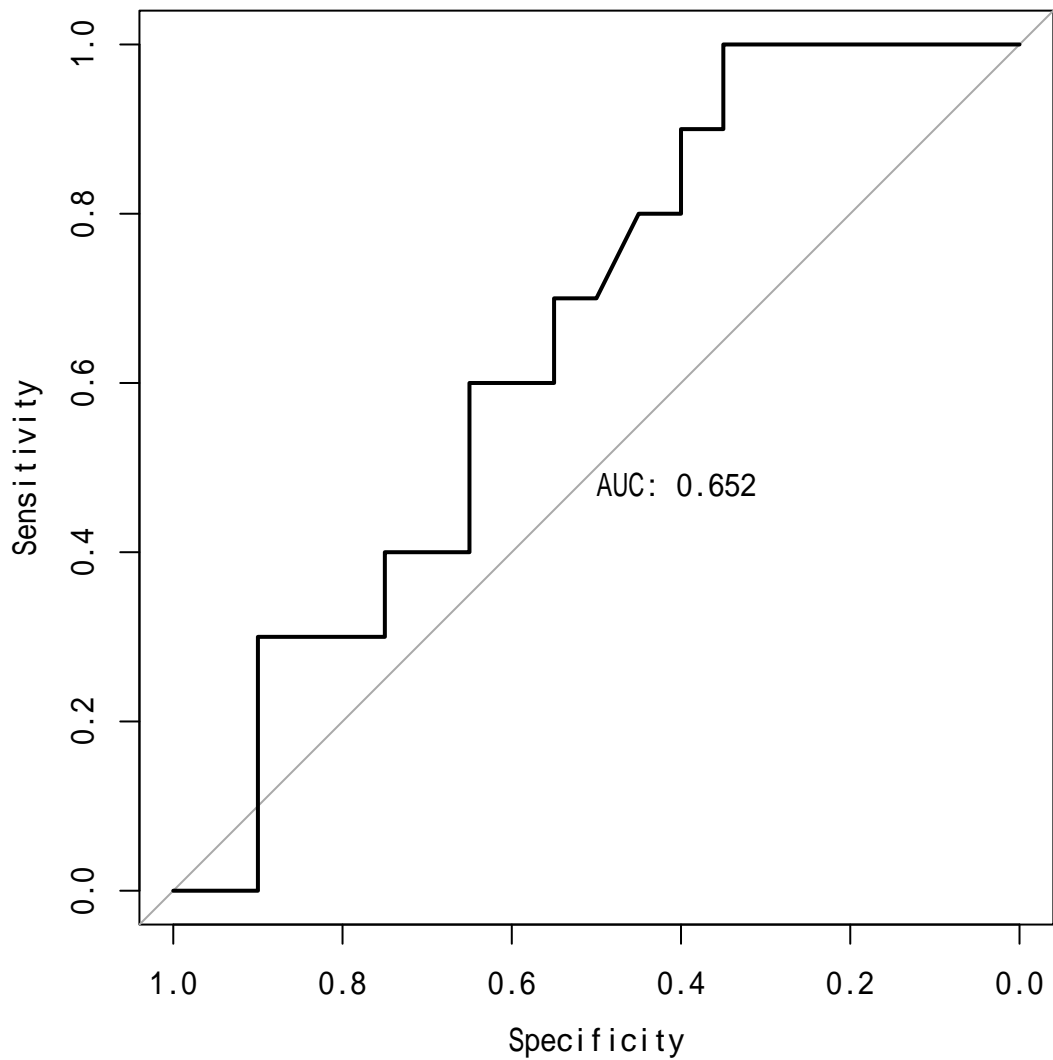

N5

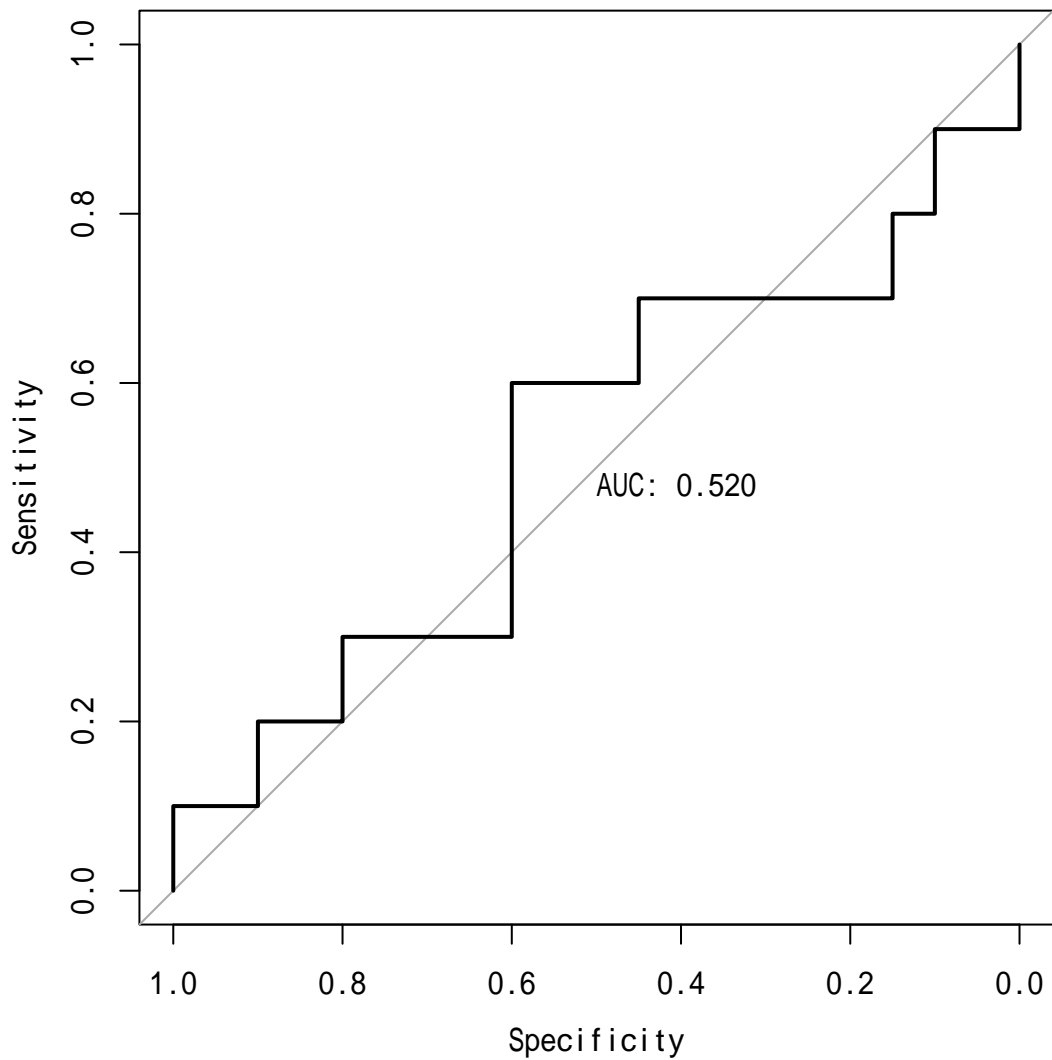

B1

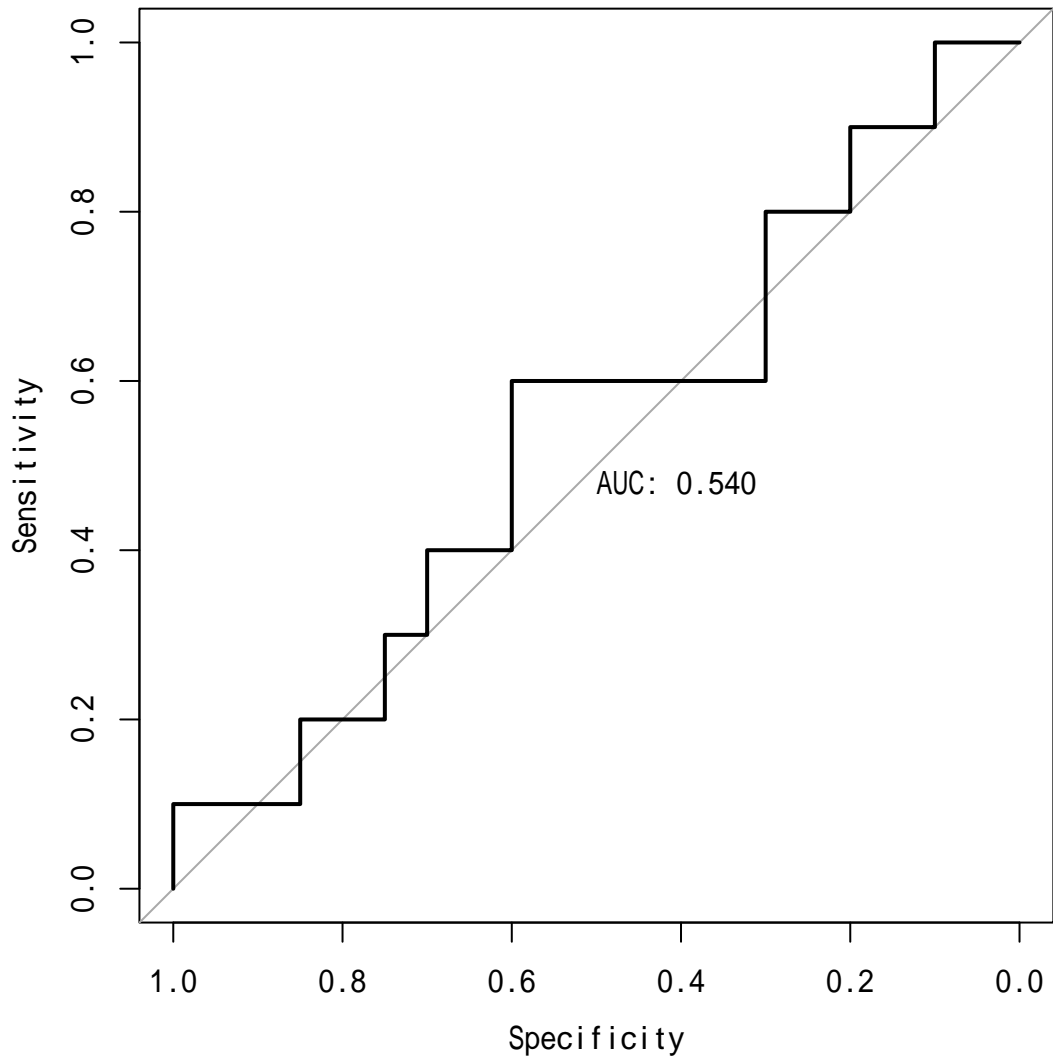

N1

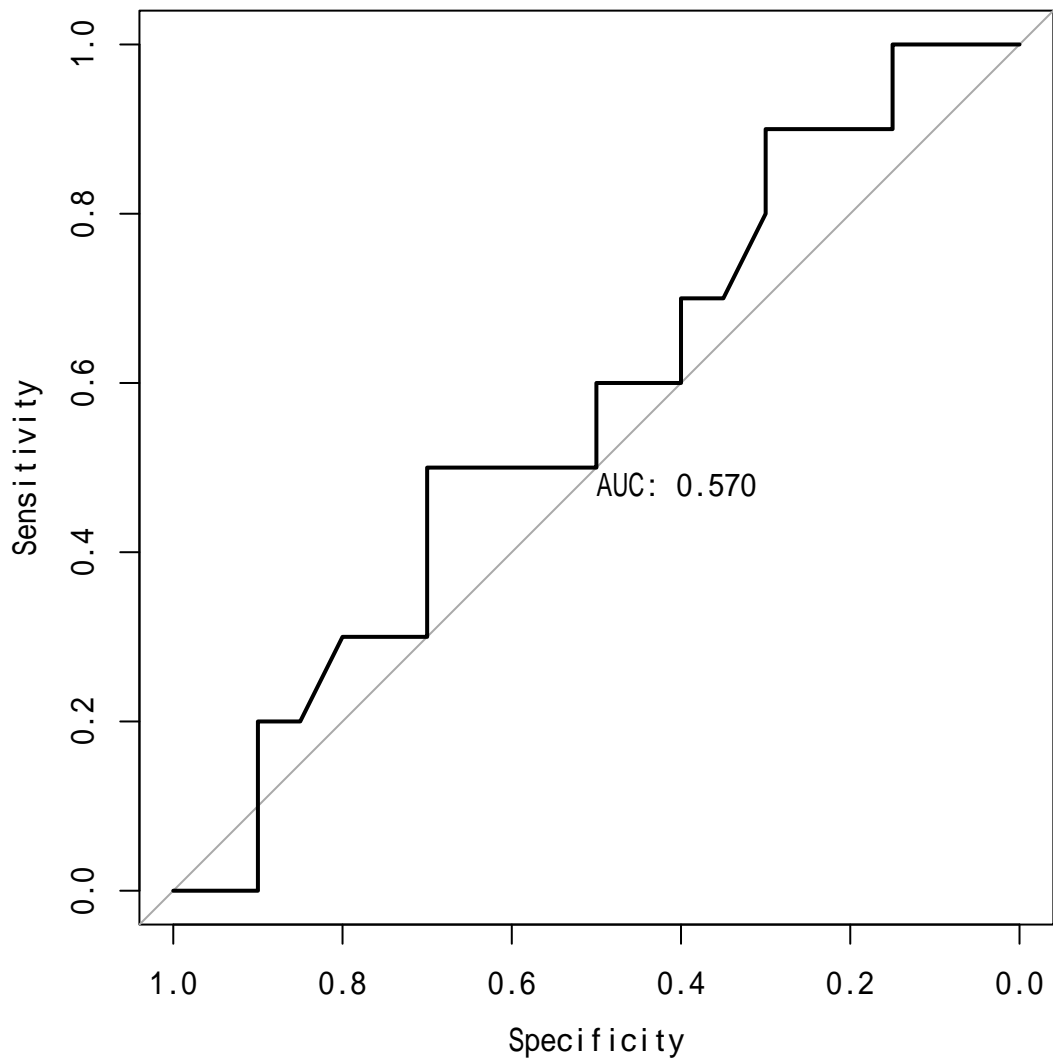

N28

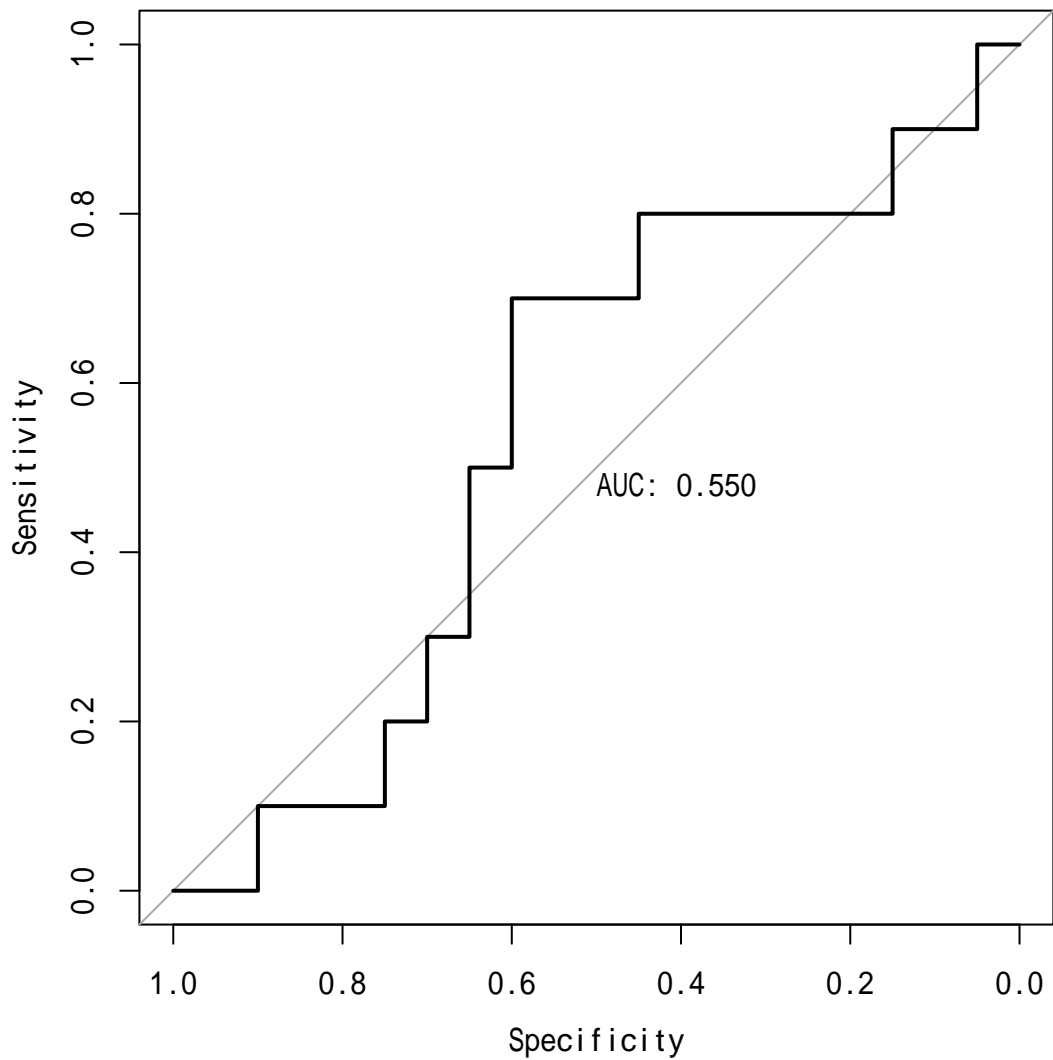

N12

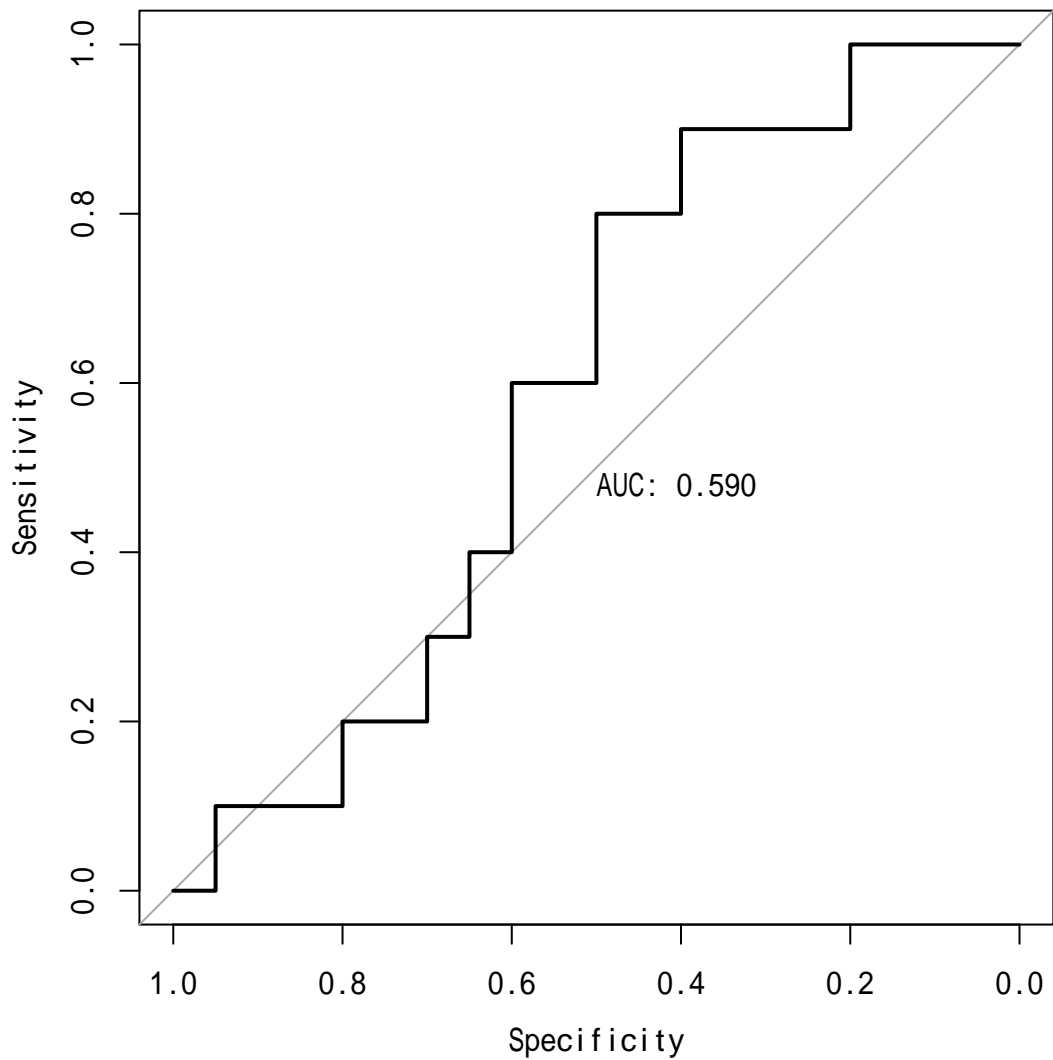

Ratio

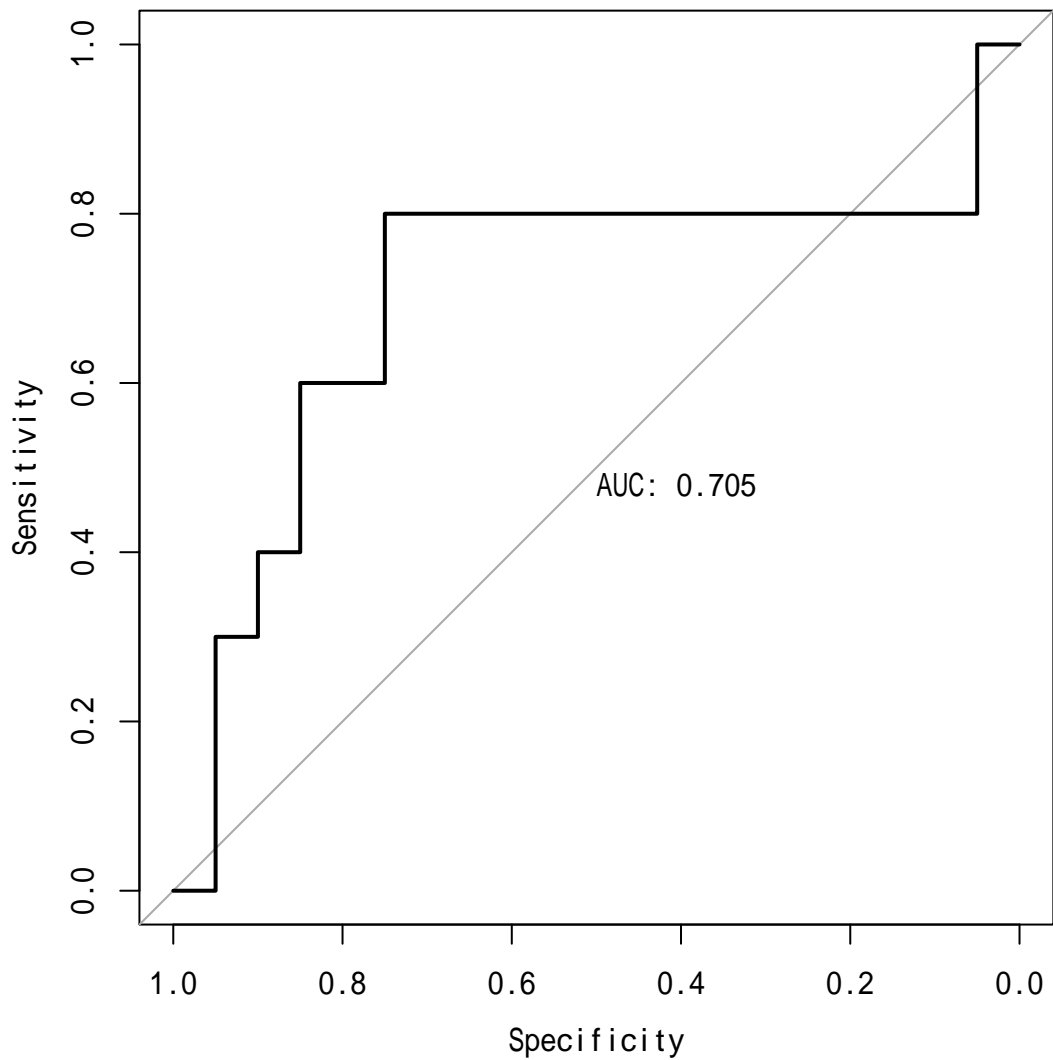

Supplement: Supplementary file 1 [file metabolites-12-00587-s001.zip › Supplementary Figure S5.pdf]

# Model

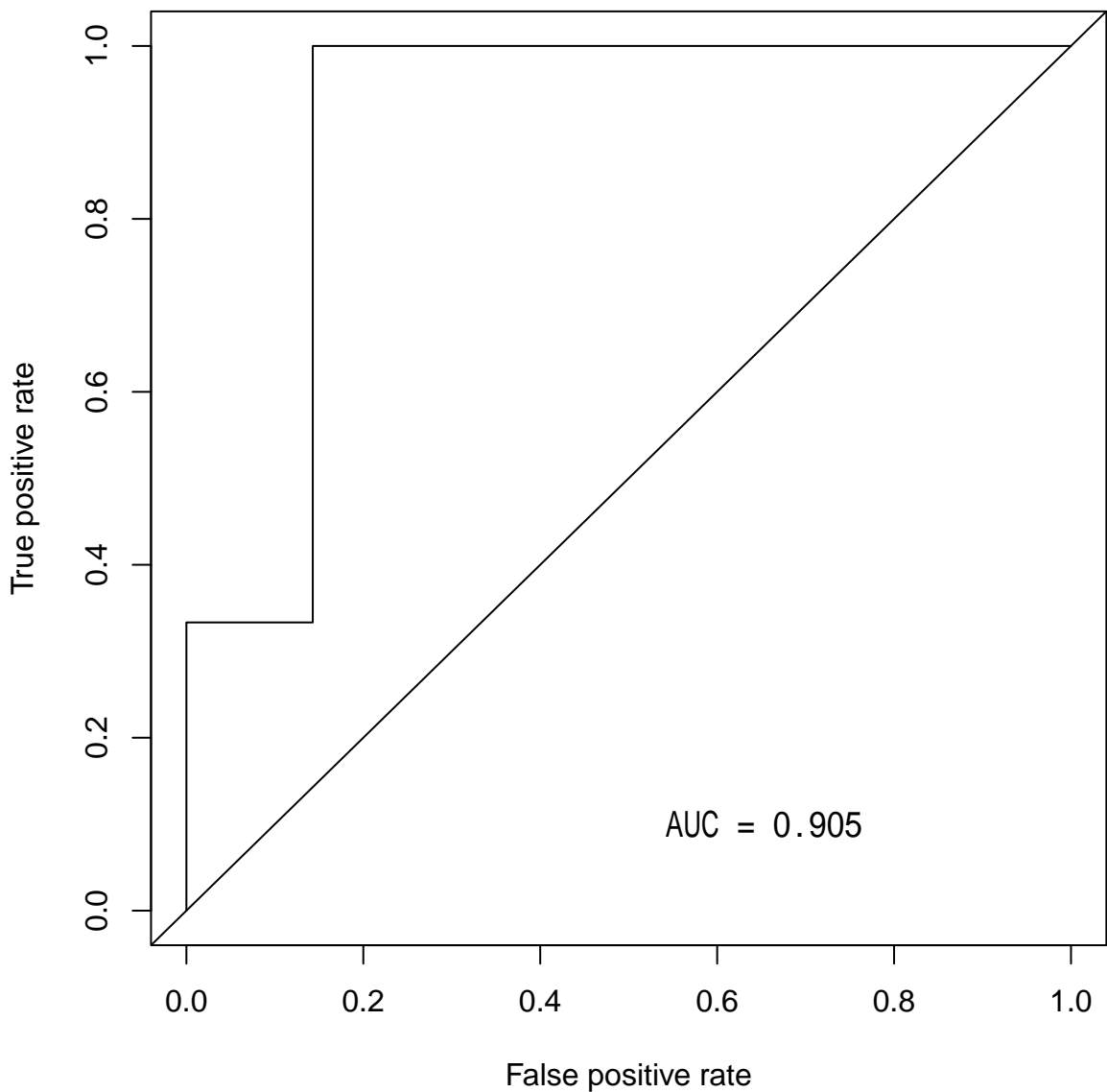

Supplement: Supplementary file 1 [file metabolites-12-00587-s001.zip › Supplementary Figure S6.pdf]

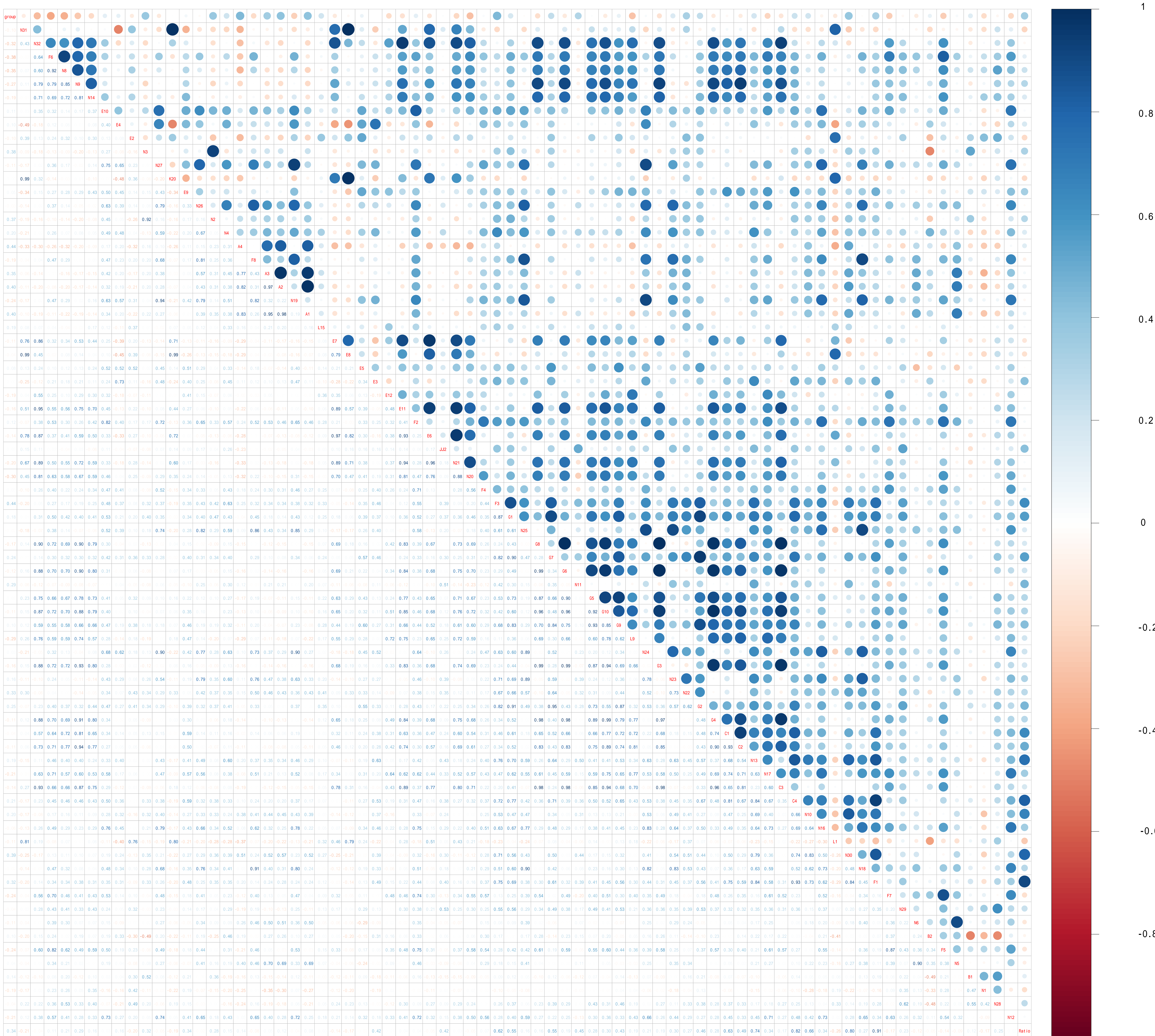

Supplement: Supplementary file 1 [file metabolites-12-00587-s001.zip › Supplementary Figure S7.pdf]
